# Tuning the Reactivity of Radical through a Triplet Diradical Cu(II) Intermediate in Radical Oxidative Cross-Coupling

**DOI:** 10.1038/srep15934

**Published:** 2015-11-03

**Authors:** Liangliang Zhou, Hong Yi, Lei Zhu, Xiaotian Qi, Hanpeng Jiang, Chao Liu, Yuqi Feng, Yu Lan, Aiwen Lei

**Affiliations:** 1 College of Chemistry and Molecular Sciences, the Institute for Advanced Studies (IAS), Wuhan University, Wuhan, Hubei 430072, P. R. China; 2 State Key Laboratory for Oxo Synthesis and Selective Oxidation, Lanzhou Institute of Chemical Physics, Chinese Academy of Sciences, Lanzhou 730000, P. R. China; 3 School of Chemistry and Chemical Engineering, Chongqing University, Chongqing 400030, P.R. China; 4 College of Chemistry and Molecular Sciences, Wuhan University, Wuhan, Hubei 430072, P. R. China

## Abstract

Highly selective radical/radical cross-coupling is paid more attention in bond formations. However, due to their intrinsic active properties, radical species are apt to achieve homo-coupling instead of cross-coupling, which makes the selective cross-coupling as a great challenge and almost untouched. Herein a notable strategy to accomplish direct radical/radical oxidative cross-coupling has been demonstrated, that is metal tuning a transient radical to a persistent radical intermediate followed by coupling with another transient radical. Here, a transient nitrogen-centered radical is tuned to a persistent radical complex by copper catalyst, followed by coupling with a transient allylic carbon-centered radical. Firstly, nitrogen-centered radical generated from *N*-methoxybenzamide stabilized by copper catalyst was successfully observed by EPR. Then DFT calculations revealed that a triplet diradical Cu(II) complex formed from the chelation *N*-methoxybenzamide nitrogen-centered radical to Cu(II) is a persistent radical species. Moreover, conceivable nitrogen-centered radical Cu(II) complex was observed by high-resolution electrospray ionization mass spectrometry (ESI-MS). Ultimately, various allylic amides derivatives were obtained in good yields by adopting this strategy, which might inspire a novel and promising landscape in radical chemistry.

The direct functionalization of C–H bonds represents a powerful step-economic tool in organic synthesis^1^,^2^,^3^,^4^,^5^,^6^,^7^. Right now, most of the research objects are mainly focused on sp and sp^2^ C-H bonds in this realm^8^,^9^,^10^,^11^,^12^. Relative to the unsaturated C-H bonds, few examples on the functionalization of sp^3^ C-H bonds, which are abundant in nature, have been reported, partially due to their weak acidity and strong bond dissociation energy (BDE)^13^. The current strategy is mainly concentrated on functionalization of benzyl^14^, α C-H bonds to heteroatoms^15^,^16^ or *tert*-butyl groups due to the impossibility of β-hydride elimination^17^. However, these researches are far from satisfaction and also can’t cater to the urgent demands for the development of more diverse and powerful synthetic strategies. Herein, we would like to ponder over a novel method for the direct functionalization of general unreactive sp^3^ C-H bonds^18^.

From the aspect of electron transfer, there are three modes for sp^3^ C-H activation processes involved metals: i): deprotonation, zero electron process^19^; ii): oxidative addition, two electron process;^20^,^21^,^22^,^23^; iii): metalloradical activation, one electron process^24^,^25^,^26^,^27^. Due to the weak acidity of inert sp^3^ C-H bonds, direct deprotonation is very difficult. While, oxidation addition of C-H bonds is usually promoted by notable metals. Therefore, the metalloradical activation of simple sp^3^ C-H bonds can be regarded as a good choice. However, in the field of radical chemistry, the most challenging problem is the homo-coupling of radicals, since most of the radicals are extremely active species so that the rate of homo-coupling is too fast to control the cross-coupling selectivity (Fig. 1)^28^,^29^. However, if a persistent radical and a transient radical simultaneously exist in one reaction system, selective cross-coupling would probably occur according to the persistent radical effect^30^,^31^. As we know, radical species could be affected through some patterns by transition metals^32^,^33^, which can catalyze radical reaction^34^. Only if a metal additive has the ability to transform an unstable radical into a stable one in some way, realizing radical cross-coupling will become a relatively easy task. Based on our efforts, a transient nitrogen-centered radical could be transformed into a persistent radical intermediate by copper catalyst in the form of a triplet diradical Cu(II) complex, which has been successfully characterized through EPR, DFT and high-resolution ESI-MS analysis^35^,^36^. In the following, another transient radical was introduced to resolve the scientific challenge perfectly. Afterwards, we achieved a copper-mediated sp^3^ C-H/N-H radical/radical oxidative cross-coupling between *N*-alkoxyamides and allylic derivatives (Fig. 2)^37^,^38^,^39^,^40^,^41^,^42^,^43^,^44^,^45^,^46^,^47^,^48^,^49^.

## Results and Discussion

**Figure 3:**
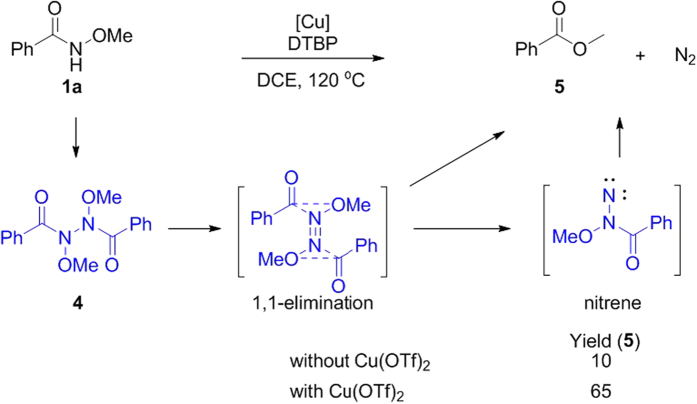
The oxidation of *N*-methoxybenzamide.

Firstly, we conducted the oxidation of *N*-methoxybenzamide with di-*tert*-butyl peroxide (DTBP) as the oxidant in 1,2-dichloroethane (DCE) at 120 °C. It was found that only 10% of methyl benzoate **5** was observed in the absence of copper(II) triflate (Cu(OTf)_2_), due to incomplete conversion of *N*-methoxybenzamide **1a**. However, methyl benzoate **5** was obtained in 65% yield when Cu(OTf)_2_ was introduced. Methyl benzoate **5** might be derived from the decomposition of *N*’-benzoyl-*N*,*N*’-dimethoxybenzohydrazide **4**, which underwent in a stepwise 1, l-elimination manner via intermediate nitrene with the concomitant generation of N_2_ at high temperature^50^,^51^. It indicates that copper plays an important role in the process. However, the role of copper is unclear (Fig. 3).

**Figure 4:**
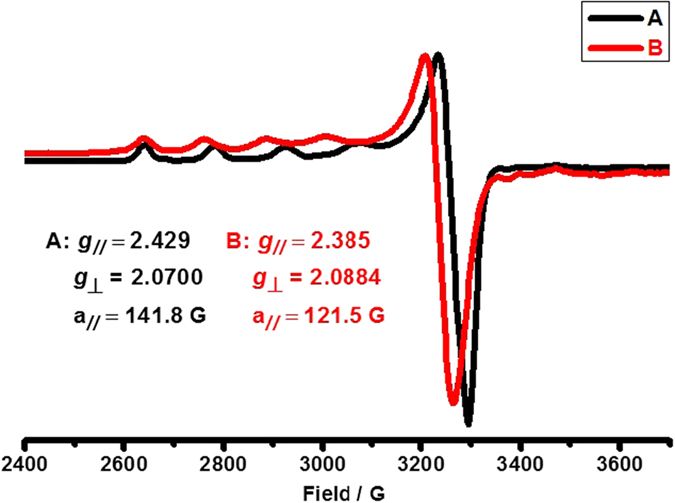
The electron paramagnetic resonance (EPR) spectra (X band, 9.4 GHz, 150 K) of (**A**): reaction mixture of Cu(OTf)_2_ and **1a** in DCE at 120 °C; (**B**): reaction mixture of Cu(OTf)_2_, ^*t*^BuOO^*t*^Bu and **1a** in DCE at 120 °C.

To study the role of Cu(OTf)_2_ in the oxidation process of *N*-methoxybenzamide, EPR analysis was carried out with **1a** and Cu(OTf)_2_ in the absence or presence of DTBP (see supplementary information for detailed reaction procedures). After mixing **1a** with Cu(OTf)_2_ in DCE for 50 min at 120 °C, EPR experiment was conducted and an EPR signal assigned to copper was observed at 150 K (Fig. 4A), *g*_//_ and *g*_⊥_ factor were respectively 2.429 and 2.0700, with coupling constant a_//_ = 141.8 G. Meanwhile, a different EPR signal of copper was detected with introduction of DTBP (Fig. 4B), *g*_//_ and *g*_⊥_ factor were respectively 2.385 and 2.0084, with coupling constant a_//_ = 121.5 G. It suggests that Cu(OTf)_2_ was coordinated by *N*-methoxybenzamide and the coordination environment of copper salt has been changed during the oxidation process.

**Figure 5:**
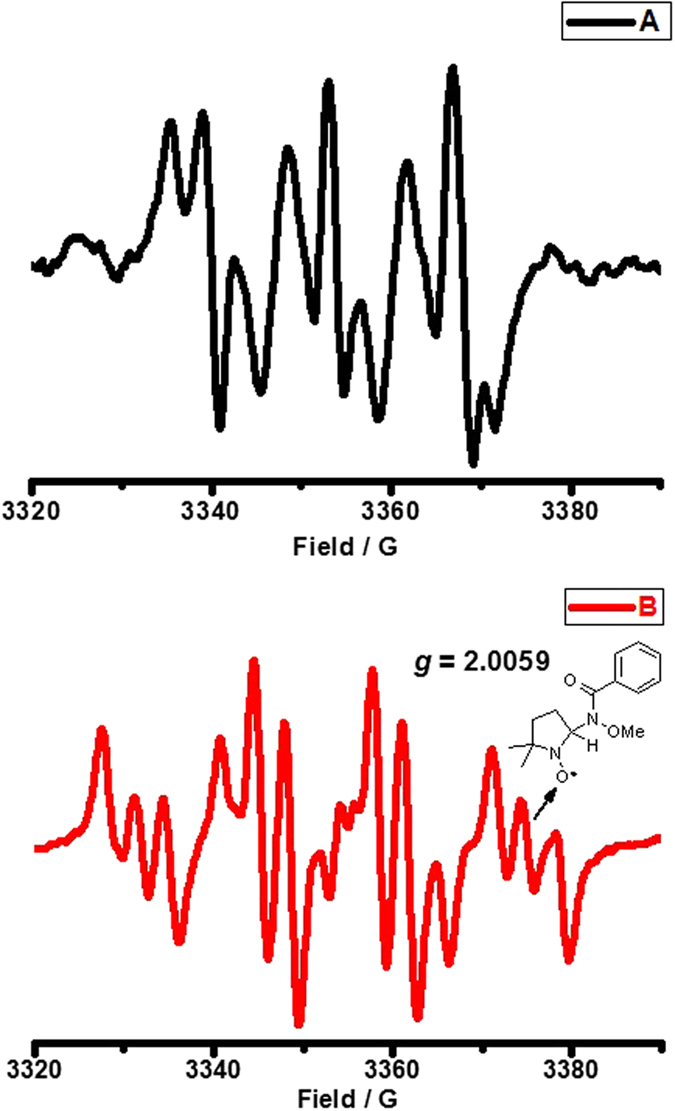
The electron paramagnetic resonance (EPR) spectra (X band, 9.4 GHz, rt) of (**A**): reaction mixture of **1a** and ^*t*^BuOO^*t*^Bu in DCE at 120 °C with the addition of DMPO; (**B**): reaction mixture of Cu(OTf)_2_, **1a** and ^*t*^BuOO^*t*^Bu in DCE at 120 °C with the addition of DMPO. DMPO = 5,5-dimethyl-1-pyrroline N-oxide

Furthermore, EPR analysis was carried out to probe the possible organic radical intermediates in the absence or presence of copper (see supplementary information for details). Firstly, a mixture of **1a** and DTBP in DCE was heated at 120 °C. Upon completion of the reaction, 5,5-dimethyl-1-pyrroline N-oxide (DMPO) was added and the reaction system was allowed to be observed by EPR at room temperature. As a result, an EPR signal from the decomposition of DTBP was detected instead of nitrogen-centered radical. (Fig. 5A). After introducing Cu(OTf)_2_ into the reaction system (Notably, other metal salts were also tested, see Figs S1 and S2 in supplementary information for details), the desired nitrogen-centered radical was successfully detected (Fig. 5B)^52^,^53^, where the *g* factor was 2.0059. The above results might demonstrate the existence of nitrogen-centered radical and stabilization effect of Cu(OTf)_2_ to nitrogen-centered radical.


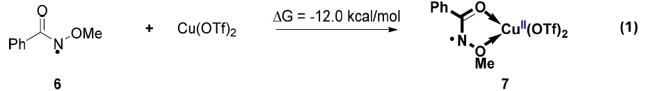



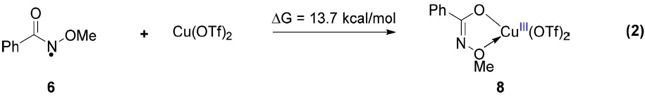


**Figure 6:**
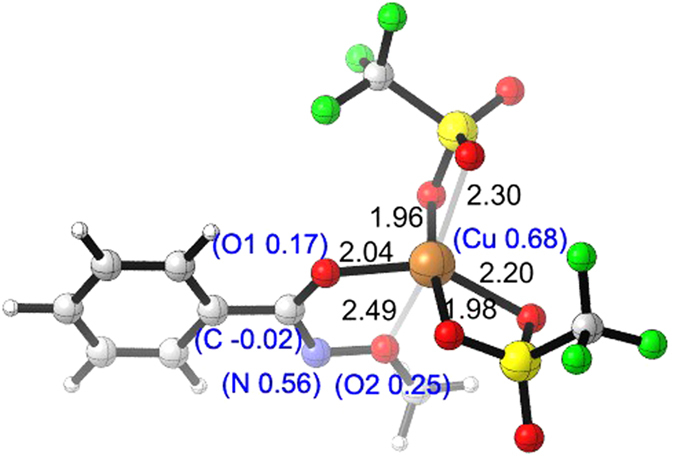
The geometry information of triplet diradical Cu(II) complex 7.

To further clarify the detailed interaction between nitrogen-centered radical and Cu(OTf)_2_, density functional theory (DFT) calculation employing method B3-LYP was carried out^54^,^55^. In the presence of Cu(OTf)_2_, nitrogen-centered radical **6** could be stabilized by 12.0 kcal/mol through the generation of a triplet diradical Cu(II) complex **7** (eq 1). However, the formation of the corresponding singlet Cu(III) complex **8** was 13.7 kcal/mol endothermic (eq 2). And we also conducted the calculation of Cu(II) complex coordinated by nitrogen-centered radical, which is still less stable than Cu(II) complex **7** (See Fig. S4, **6a** in supplementary information for details). Subsequent distribution of spin density shown in Fig. 6 also indicated that the oxidation state of Cu is +2 due to the located large spin. Another unpaired electron was shared by nitrogen and two oxygen atoms, which accounted for the stability of **7**.

**Figure 7:**
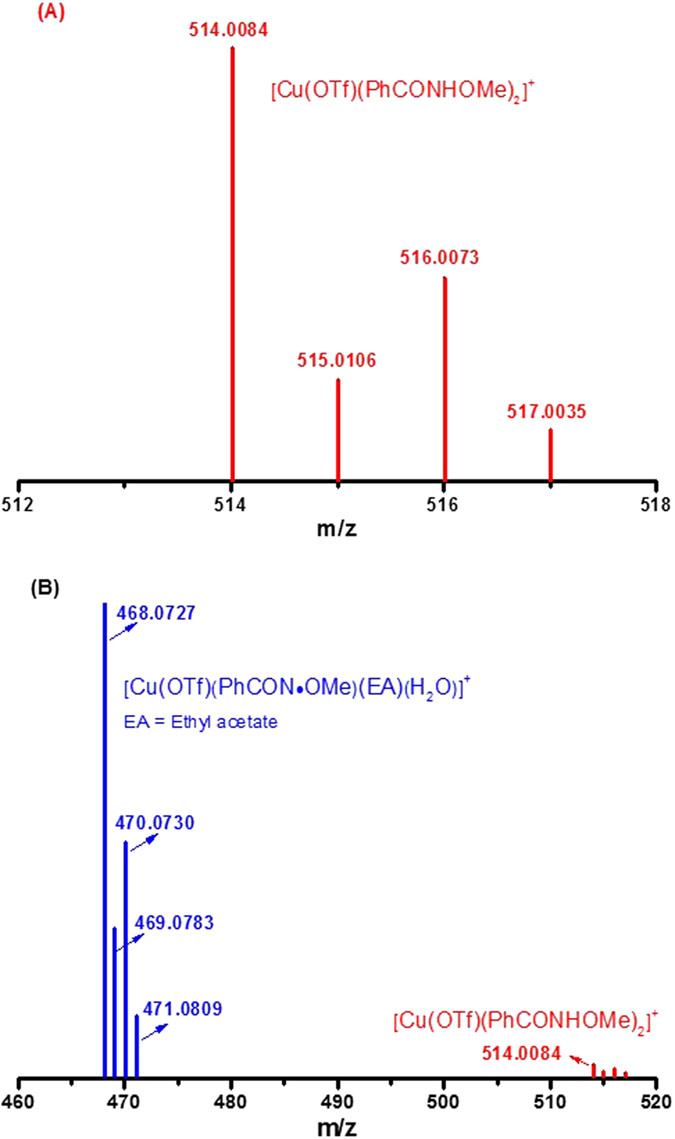
High-resolution ESI-MS analysis (rt) of the reaction (**A**): reaction mixture of Cu(OTf)_2_ and **1a** in ethyl acetate at 120 °C for 1 h; (**B**): reaction mixture of Cu(OTf)_2_, **1a** and ^*t*^BuOO^*t*^Bu in ethyl acetate at 120 °C for 1 h.

In order to get sufficient evidence to elaborate the interaction between copper and nitrogen-centered radical, high-resolution electrospray ionization mass spectrometry (ESI-MS) analysis was performed. As shown in Fig. 7, at the beginning, the complex between Cu(OTf)_2_ and **1a** was formed, a set of peaks at main m/z 514.0084 that corresponded to cationic complex [Cu(OTf)(PhCONHOMe)_2_]^+^ were found (Fig. 7A). When ESI-MS analysis of the reaction about Cu(OTf)_2_, **1a** and DTBP was carried out, it was found that the set of peaks at main m/z 514.0084 decayed dramatically while another set of peaks at main m/z 468.0727 appeared, which might correspond to cationic-radical complex [Cu(OTf)(PhCON˙OMe)(EA)(H_2_O)]^+^ (Fig. 7B (Blue), EA = Ethyl acetate). These results were highly consistent with EPR experiments (Fig. 4A,B).

Up to now, the interaction between Cu(OTf)_2_ and nitrogen-centered radical have been demonstrated, which may exist in the stable form of a triplet diradical Cu(II) complex. Here, the different results for generation of methyl benzoate in the Fig. 3 can be illustrated. A persistent copper-nitrogen-centered radical from chelation between nitrogen-centered radical and copper more readily coupled with another transient nitrogen-centered radical to produce intermediate **4** with the existence of copper (Fig. 3). Based on our initial assumption, if a transient radical is introduced into the system of *N*-methoxybenzamide **1a** and Cu(OTf)_2_, the highly selective cross-coupled product could be obtained. Cyclohexene was chosen as a precursor of transient carbon-centered radical to verify our proposal. Since the cyclohexene could be easily oxidized into allylic carbon-centered radical by DTBP^56^, and the corresponding allylic amine derivatives extensively exist in natural products and pharmaceuticals^57^,^58^, achieving direct synthesis of allylic amines from simple allylic hydrocarbons and amines should be charming and significant.

To testify the feasibility of cross-coupling between stable nitrogen-centered radical complex and transient carbon-centered radical, firstly, we got some viable results by DFT. Computational results suggested that the generation of nitrogen-centered radical **6** is favorable compared with that of **9** (See Fig. S3 in supplementary information for details). For the interaction between carbon-centered radical **9** and Cu(OTf)_2_, however, the formation of both triplet diradical Cu(II) complex and singlet Cu(III) complex are all endothermic (see Fig. S4 in supplementary information for details), which indicates that radical **9** cannot be stabilized by Cu(OTf)_2_ compared to radical **6**. Consequently, the copper stabilized nitrogen-centered radical complex **7** is supposed to be a persistent radical, enabling cross-coupling with newly generated transient radical **9** to afford the Cu(II) complex **10** with 34.3 kcal/mol exothermic (eq 3).


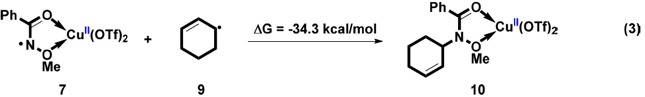


**Figure 8:**
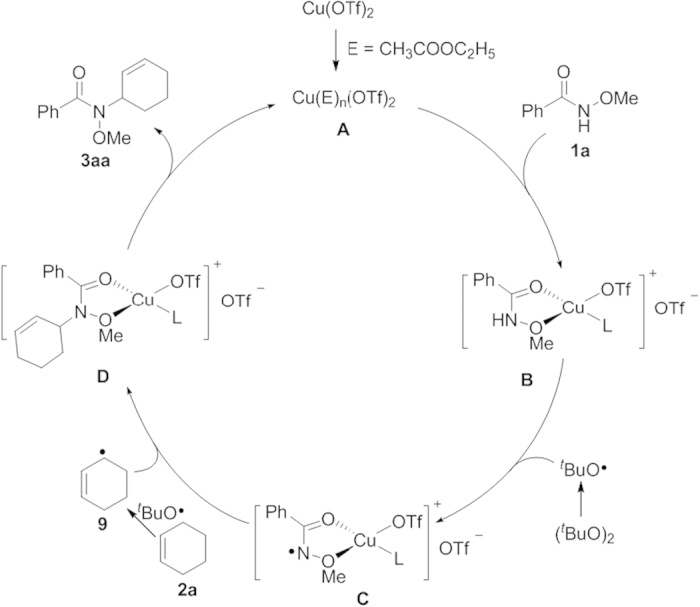
Design of Cu(II)-mediated C-H/N-H radical oxidative cross-coupling.

On above basis, a plausible pathway for direct radical/radical cross-coupling is designed in Fig. 8. Initially, coordination of copper(II) triflate to ethyl acetate followed by the ligand exchange of complex **A** by **1a** produced Cu(II) complex **B**, which was confirmed by high-resolution ESI-MS. Subsequently, hydrogen abstraction of complex **B** by ^*t*^BuO radical generated from homolytic cleavage of DTBP resulted in generation of Cu(II) intermediate **C** chelated by two oxygen atom of nitrogen-centered radical species. In the following, the complex **D** was given highly selectively through the cross-coupling of nitrogen-centered radical Cu(II) species **C** and carbon-centered radical **9** from **2a**. Finally, complex **D** released the desired product **3aa** and Cu(OTf)_2_ to furnish the whole catalytic cycle. The possible pathway of cyclohexene radical **9** homolytic substituent to Cu(III)-nitrogen complex was excluded^59^,^60^ due to instability of Cu(III) complex.

Afterwards, we tried to optimize the reaction conditions employing **1a** and **2a** as model substrates based on the above research (see Table S1 in supplementary information for details). It was noted that during the transform, homo-coupling of **2a** was also observed by GC-MS, which was further indicative of existence of allylic carbon-centered radical. After considerable efforts, we determined the optimized conditions to be: **1a** (0.5 mmol), **2a** (2 mL), Cu(OTf)_2_ (0.01 mmol, 2 mol%), DTBP (1.25 mmol, 2.5 equiv), ethyl acetate (0.5 mL), 120 °C, 9 h. Although **2a** was excess, it could be reused more than three times (see Table S2 for details in supplementary information).

With the optimized conditions in hand, we next sought to define the scope of other substrates First of all, various *N*-methoxybenzamide derivatives were employed to couple with cyclohexene (Table 1). *N*-methoxybenzamides with electron-donating groups on aryl rings such as Me (**3b**a), Et (**3ca**), ^*t*^Bu (**3da**) and OMe (**3ea**) could be cross-coupled efficiently with cyclohexene to deliver the corresponding products in good to excellent yields. *N*-Methoxy-3, 5-dimethylbenzamide also proceeded well, giving the coupling product in 77% yield (Table 1, **3fa**). However, substrates with electron-withdrawing groups on aryl rings are inferior to those with electron-donating substituents, since substrates with NO_2_ (**3ga**), COOMe (**3ha**) and CF_3_ (**3ia**) substituents afforded the desired products in slightly lower yields. On the other hand, para-halogen substituents such as F (**3ja**), Cl (**3ka**) and Br (**3la**) could be well tolerated. Meanwhile, *N*-methoxybenzamides with meta-substituents on aryl rings were also found to be good coupling partners, generating desired products in good yields (**3ma** – **3oa**). To our delight, aliphatic amide was also suitable substrate, giving the desired product (**3pa**). It is interesting to note that substrates with other aromatic groups such as naphthalene (**3qa**), furan (**3ra**) and thiophene (**3sa**) were also feasible. The yield for **3sa** is relatively low because of the incomplete conversion of the starting material.

We next examined the scope of allylic substrates that participated in this C−H amination reaction. As summarized in Table 2, a wide range of cyclopentene (**3ab**), (*Z*)-cyclooctene (**3ac** and **3ad**) and (*Z*)-cyclododecene (**3ae**) allylic substrates underwent C−H amination under the modulate conditions. It is very satisfactory that long-chain aliphatic allylic substrates could also couple with **1a**. For instance, oct-1-ene (**3af**) and dodec-1-ene (**3ag**) were all suitable substrates, giving the branch products in moderate yield. But, to be our surprise, when allylbenzene was applied, *N*-cinnamyl-*N*-methoxybenzamide (**3ah**) was isolated. Maybe, allybenzene was isomerized during the process because of conjugation from benzene. On the other hand, the primary allylic C-H was also tolerated. Prop-1-en-2-ylbenzene gave moderate yield (**3ai**).

In conclusion, a novel strategy to promote radical/radical oxidative cross-coupling have been demonstrated, where metal-additive stabilizes one of both transient radicals to accomplish direct C-H/N-H radical/radical oxidative cross-coupling for construction of allylic amine. Nitrogen-centered radical, compared to allylic carbon-centered radical, is preferably stabilized through a triplet diradical Cu(II) complex, which is characterized as a key persistent radical intermediate through EPR, DFT and ESI-MS, so as to furnish cross-coupling with a transient allylic carbon-centered radical for a direct formation of C-N bonds. This discovery may open up a brand-new concept and promising landscape for radical reaction. More extension of this strategy is currently ongoing in our laboratory.

## Methods

### General procedure for preparation of *N*-methoxybenzamide

Into a round-bottom flask with a stir bar are added methoxyammonium chloride (11 mmol, 1.1 equiv), Potassium carbonate (22 mmol, 2.2 equiv), water (10 mL) and ethyl acetate (20 mL) at 0 °C. Then benzoyl chloride (10 mmol) was added into the flask dropwise. After adding benzoyl chloride, the flask was allowed to put into room temperature. Upon completion of the reaction by TLC analysis, it was extracted with ethyl acetate. The organic layers were combined and dried over anhydrous Na_2_SO_4_. The pure product was obtained by flash column chromatography on silica gel (petroleum: ethyl acetate = 1:1).

### General procedure for oxidation of *N*-methoxybenzamide

In an oven-dried tube equipped with a stir bar, Cu(OTf)_2_ (0.01mmol, 2 mol%) (or as required with no addition) and *N*-methoxybenzamide **1a** (0.50 mmol) were combined and sealed. The tube was then charged with nitrogen and DCE (2.0 mL) was injected into the tube by syringe. Under the protection by nitrogen, DTBP (1.25 mmol, 2.5 equiv) was slowly injected into the reaction tube. The reaction was then heated to 120 °C for 9 h, and cooled down to room temperature. The yield was determined by GC with 1,1’-biphenyl as the internal standard.

### General procedure for EPR experiments

(I): In an oven-dried tube equipped with a stir bar, Cu(OTf)_2_ (0.0125mmol, 5 mol%) and *N*-methoxybenzamide **1a** (0.25 mmol) were combined and sealed. The tube was then charged with nitrogen and DCE (0.25 mL) was injected into the tube by syringe. Under the protection by nitrogen, DTBP (0.625 mmol, 2.5 equiv) (or as required with no addition) was slowly injected into the reaction tube. The reaction was then heated to 120 °C for 50 min. After that, 10 μL of the solution was taken out into a small tube. Then, this mixture was analyzed by EPR at 150 K.

(II) In an oven-dried tube equipped with a stir bar, Cu(OTf)_2_ (0.0125 mmol, 5 mol%) (or as required with no addition) and *N*-methoxybenzamide **1a** (0.25 mmol) were combined and sealed. The tube was then charged with nitrogen and DCE (0.25 mL) was injected into the tube by syringe. Under the protection by nitrogen, DTBP (0.625 mmol, 2.5 equiv) was slowly injected into the reaction tube. The reaction was then heated to 120 °C for 50 min. 10 μL DMPO (5,5-dimethyl-1-pyrroline N-oxide) was added into the system, After that, 10 μL of the solution was taken out into a small tube. Then, this mixture was analyzed by EPR at room temperature.

### General procedure for high-resolution ESI-MS experiments

In an oven-dried tube equipped with a stir bar, Cu(OTf)_2_ (0.01 mmol, 2 mol%) and *N*-methoxybenzamide **1a** (0.50 mmol) were combined and sealed. The tube was then charged with nitrogen and ethyl acetate (2.0 mL) was injected into the tube by syringe. Under the protection by nitrogen, DTBP (1.25 mmol, 2.5 equiv) (or as required with no addition) was slowly injected into the reaction tube. The reaction was then heated to 120 °C for 1 h. Twenty microliter of this reaction solution was directly taken out for high-resolution ESI-MS analysis at room temperature.

### General procedure for the oxidative radical/radical cross-coupling

In an oven-dried tube equipped with a stir bar, Cu(OTf)_2_ (0.01 mmol, 2 mol%) and *N*-methoxybenzamide **1a** (0.50 mmol) were combined and sealed. The tube was then charged with nitrogen, ethyl acetate (0.5 mL) and cyclohexene **2a** (2.0 mL) were successively injected into the tube by syringe. Under the protection by nitrogen, DTBP (1.25 mmol, 2.5 equiv) was slowly injected into the reaction tube. The reaction was then put into oil bath under 120 °C. After stirring for 9 h, the reaction was cooled down to room temperature and quenched with saturated Na_2_S_2_O_3_ solution. After extraction with ethyl acetate (3 x 10 mL), the organic layers were combined and dried over anhydrous Na_2_SO_4_. The pure product was obtained by flash column chromatography on silica gel (petroleum: ethyl acetate = 50:1 - 5:1).

## Additional Information

**How to cite this article**: Zhou, L. *et al.* Tuning the Reactivity of Radical through a Triplet Diradical Cu(II) Intermediate in Radical Oxidative Cross-Coupling. *Sci. Rep.* **5**, 15934; doi: 10.1038/srep15934 (2015).

## Supplementary Material

Supplementary Information

## Figures and Tables

**Figure 1:**
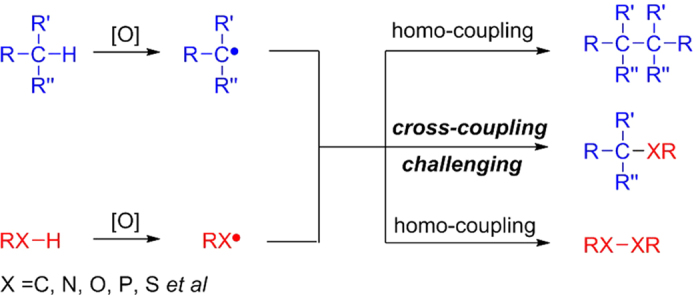
Design of radical/radical oxidative cross-coupling.

**Figure 2:**
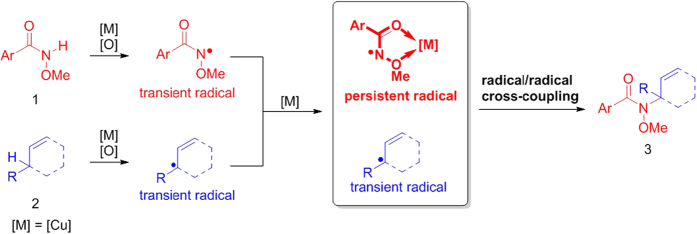
Tuning the reactivity of nitrogen-centered radical to accomplish N-H/C-H radical/radical oxidative cross-coupling under copper catalysis.

**Table 1:**
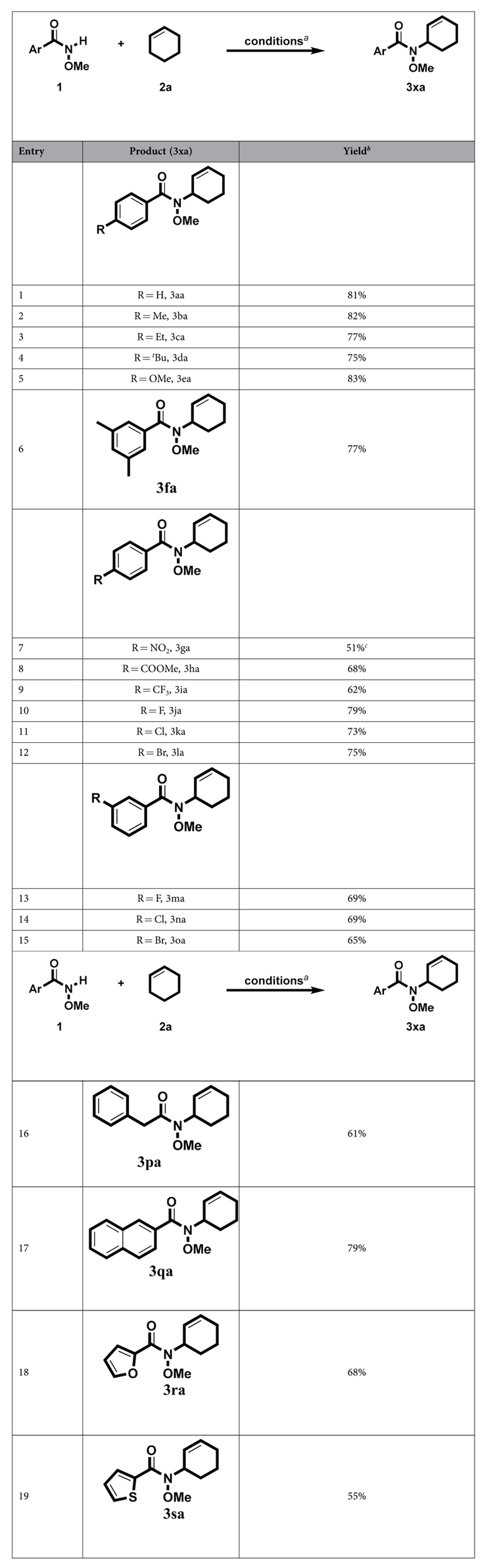
Scope of the N-methoxyarylamide.

^a^Unless otherwise noted, the reaction conditions were as follows: **1** (0.5 mmol), **2a** (2 mL), [Cu(OTf)_2_] (0.01 mmol), DTBP (1.25 mmol), 0.5 mL EtOAc, 120 °C, 9 h. ^b^Yields of isolated products. ^c^**2a** (3 mL). DTBP = Di-*tert*-butyl peroxide.

**Table 2:**
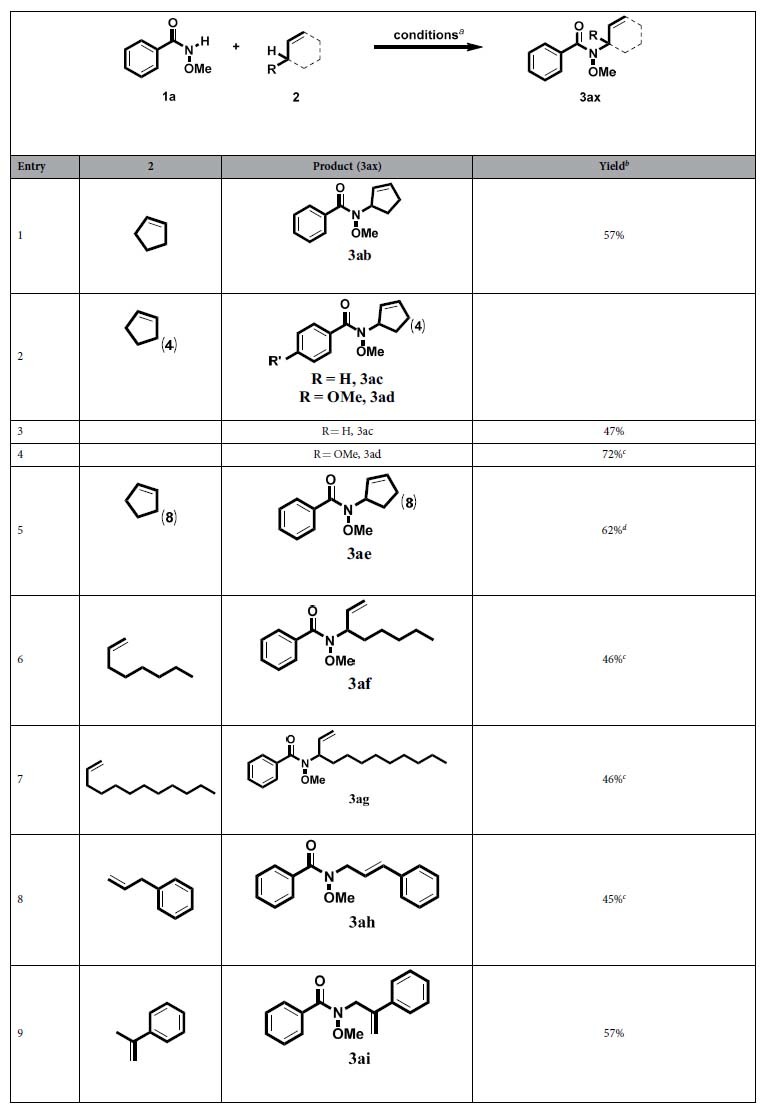
Scope of the allylic substrates.

^***a***^Unless otherwise noted, the reaction conditions were as follows: **1a** (0.5 mmol), **2** (3 mL), [Cu(OTf)_2_] (0.01 mmol), DTBP (1.25 mmol), EtOAc (0.5 mL), 120 °C, 9 h. ^*b*^Yields of isolated products. ^*c*^DTBP (2.0 mmol). DTBP = Di-*tert*-butyl peroxide. ^*d*^(*Z*)-cyclododecene (2 ml).
