# Supplementary material for: Tuning the Reactivity of Radical through a Triplet Diradical Cu(II) Intermediate in Radical Oxidative Cross-Coupling
Source: Sci Rep. 2015 Nov 3;5:15934. doi: 10.1038/srep15934 (PMC4630646; doi:10.1038/srep15934)
Supplement: Supplementary Information [file srep15934-s1.pdf]

Supplementary Information

Tuning the Reactivity of Radical through a Triplet Diradical Cu(II) Intermediate in Radical Oxidative Cross-Coupling

Liangliang Zhou[1], Hong Yi[1], Lei Zhu[3], Xiaotian Qi[3], Hanpeng Jiang[4], Chao Liu[1], Yuqi Feng [4], Yu Lan[3] & Aiwen Lei[1,2]

[1] College of Chemistry and Molecular Sciences, the Institute for Advanced Studies (IAS), Wuhan University, Wuhan, Hubei 430072, P. R. China., [2] State Key Laboratory for Oxo Synthesis and Selective Oxidation, Lanzhou Institute of Chemical Physics, Chinese Academy of Sciences Lanzhou, Gansu 730000, P. R. China. [3] School of Chemistry and Chemical Engineering Chongqing University, Chongqing 400030, P. R. China. [4] College of Chemistry and Molecular Sciences, Wuhan University, Wuhan, Hubei 430072, P. R. China. Correspondence and requests for materials should be addressed to A.W.L. (aiwenlei@whu.edu.cn).

General information……………………………………………………….………......…S2

General procedure of the EPR experiments.......………………………..………………...S3

DFT details for the generation of radicals, Cu(II) complex coordinated by nitrogen-centered radical and interaction between cyclohexene carbon-centered radical and copper…………………………………………………………………………………...S5

Condition optimization of the C(sp^3)-H/N-H radical oxidative cross-coupling…………..S6

Reusing of cyclohexene 2a……………………………………………………….……………S8

General procedure for the C(sp^3)-H/N-H radical oxidative cross-coupling.....…..........… S9

Detail descriptions for products……………………………………………....…....…..…S10

References……………………..……………………………………………......…......…S19

Copies of product ^{1}H NMR, ^{13}C NMR and ^{19}F NMR………………….……....……...…S20

Complete reference for Gaussian 09……………………………………………………S50

General information

All reactions were isolated from moisture and oxygen by a nitrogen atmosphere. All glassware was oven dried at 110 ^{o}C for hours and cooled down under vacuum. Unless otherwise noted, materials were obtained from commercial suppliers and used without further purification. *N*-Alkoxyamides were prepared following literature procedures.[1] Thin layer chromatography (TLC) employed glass 0.25 mm silica gel plates. Flash chromatography columns were packed with 200-300 mesh silica gel in petroleum (bp. 60-90 ^{o}C). Gas chromatographic analyses were performed on Varian GC 2000 gas chromatography instrument with a FID detector and naphthalene was added as internal standard. GC-MS spectra were recorded on a Varian GC-MS 3900-2100T. EPR spectra were recorded on a Bruker A-200 spectrometer. ^{1}H and ^{13}C NMR data were recorded with Bruker Advance III (400 MHz) spectrometers with tetramethylsilane as an internal standard. All chemical shifts (δ) are reported in ppm and coupling constants (*J*) in Hz. All chemical shifts are reported relative to tetramethylsilane and d-solvent peaks (77.00 ppm, chloroform), respectively. High resolution mass spectra (HRMS) were measured with a Waters Micromass GCT instrument, accurate masses are reported for the molecular ion ($[M]^+$). High-resolution electrospray ionization mass spectrometry (ESI-MS) analysis was carried out by MicroTOF-Q orthogonal-accelerated TOF mass spectrometer (Bruker Daltonics, Bremen, Germany) with an ESI source (Turbo Ionspray)

General procedure of the EPR experiments

(A) Detecting organic radical: to an oven-dried tube equipped a stir bar, metal additives (5 mol%, as required with no addition), *N*-methoxybenzamide (38.0 mg, 0.25 mmol) was added 1,2-dichloroethane (0.25 mL) under N_2 atmosphere, then DTBP (91 mg, 0.625 mmol) was injected via a microsyringe continuously. After that, the Schlenk tube was allowed to be heated to 120 ^{o}C for 50 min. 10 μL DMPO (5,5-dimethyl-1-pyrroline N-oxide) was added into the system, followed by 10 μL of the solution was taken out into a small tube. Then, this mixture was analyzed by EPR at room temperature. The EPR spectrums are shown in Fig. S1 and Fig. S2

(B) Detecting [Cu] radical: to an oven-dried tube equipped a stir bar, $Cu(OTf)_2$ (4.5 mg, 0.0125mmol), *N*-methoxybenzamide (38.0 mg, 0.25 mmol) was added 1,2 dichloroethane (0.25 mL) under N_2 atmosphere. After that, the Schlenk tube was allowed to be heated to 120 ^{o}C for 50 min, followed by 10 μL of the solution was taken out into a small tube. Then, this mixture was analyzed by EPR at 150 K

(C) Detecting [Cu] radical: to an oven-dried tube equipped a stir bar, $Cu(OTf)_2$ (4.5 mg, 0.0125mmol), *N*-methoxybenzamide (38.0 mg, 0.25 mmol) was added 1,2 dichloroethane (0.25 mL) under N_2 atmosphere, then DTBP (91 mg, 0.625 mmol) was injected via a microsyringe. After that, the Schlenk tube was allowed to be heated to 120 ^{o}C for 50 min, followed by 10 μL of the solution was taken out into a small tube. Then, this mixture was analyzed by EPR at 150 K

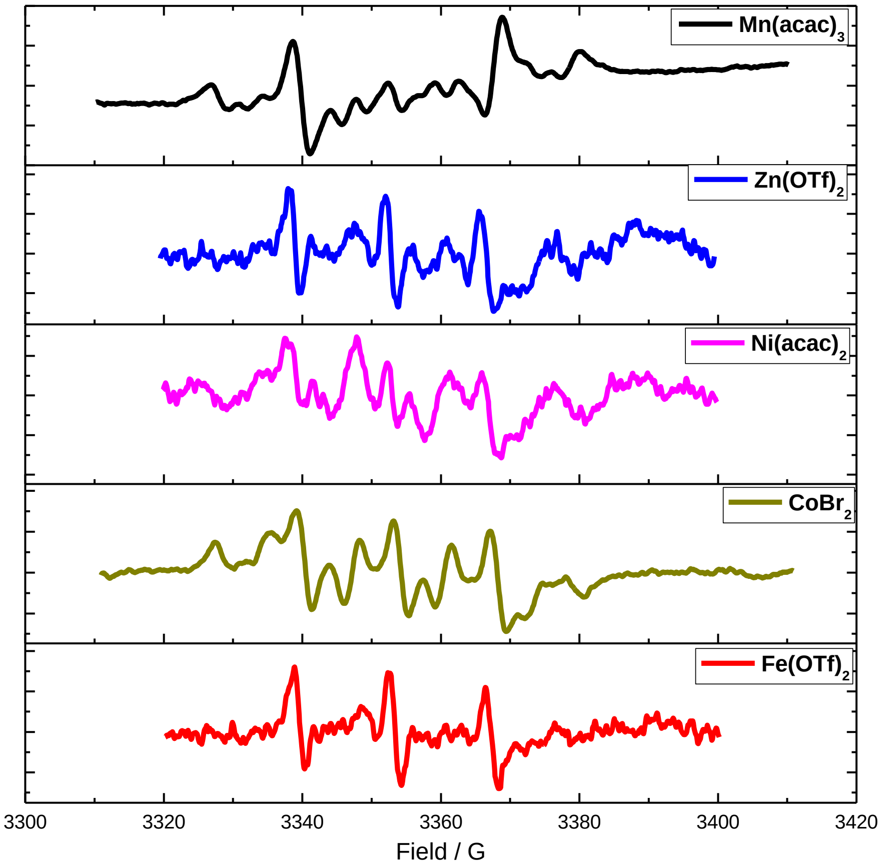

Figure S1. The electron paramagnetic resonance (EPR) spectra (X band, 9.4 GHz, rt) of reaction mixture of [M], 1a and ^{t}BuOO^{t}Bu in DCE at 120 °C with the addition of DMPO.

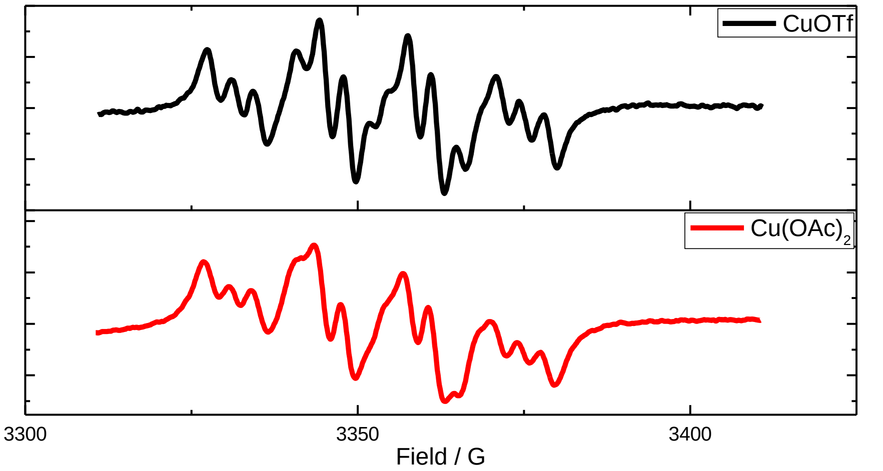

Figure S2. The electron paramagnetic resonance (EPR) spectra (X band, 9.4 GHz, rt) of reaction mixture of [Cu], **1a** and ^{t}BuOO^{t}Bu in DCE at 120 °C with the addition of DMPO.

DFT details for the generation of radicals, Cu(II) complex coordinated by nitrogen-centered radical and interaction between cyclohexene carbon radical and copper

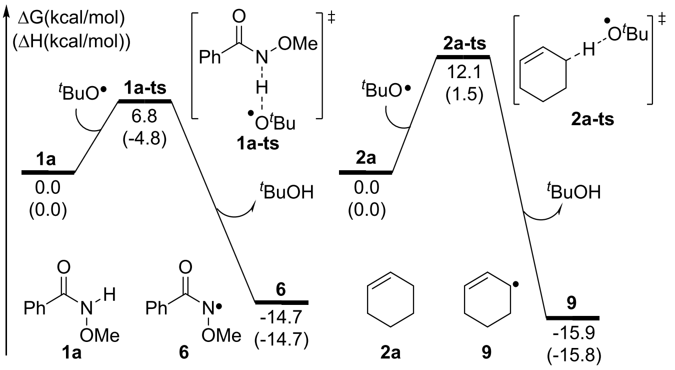

Figure S3. Free energy profile for the generation of nitrogen radical 6 and cyclohexene carbon-centered radical 9.

The nitrogen radical **6**, which is thermodynamically 14.7 kcal/mol more stable than *tert*-butyloxyl radical, could be formed via transition state **1a-ts** by the radical substitution between **1a** and ^{t}BuO radical with a barrier of 6.8 kcal/mol. On the other hand, cyclohexene carbon radical **9** could also be generated with 15.9 kcal/mol exothermic through transition state **2a-ts**. The corresponding activation free energy is 12.1 kcal/mol, which is 5.3 kcal/mol higher than that of **1a-ts**. Therefore, the generation of **6** is favorable compared with that of **9**.

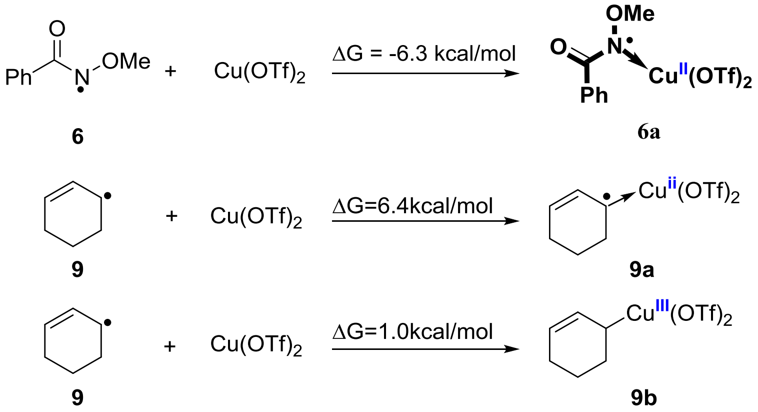

Figure S4. Grubbs free energy about Cu(II) complex coordinated by nitrogen-centered radical 6a and the interaction between carbon-centered radical 9 and $Cu(OTf)_2$.

Condition optimization of the C(sp^3)-H/N-H Radical Oxidative Cross-Coupling

Table S1. Optimization of the reaction conditions. [a]

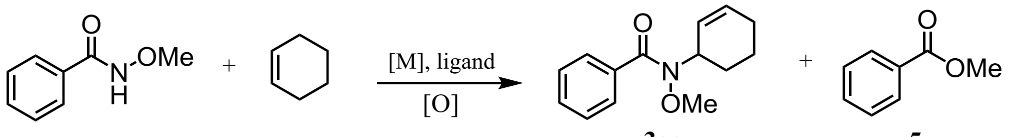

Entry	[M]	[L]	Conversion (**1a**) (%)[b]	Yield (**3aa**) (%)[b]	Yield (**5**) (%)[b]
1	Ni(acac)$_2$	TMEDA	18	7	3
2	Fe(acac)$_3$	TMEDA	95	14	3
3	Mn(acac)$_3$	TMEDA	21	7	4
4	Cu(acac)$_2$	TMEDA	90	51	7
5	CuCl	TMEDA	27	12	4
6	CuBr	TMEDA	98	50	18
7	CuI	TMEDA	96	46	17
8	CuTc	TMEDA	85	44	8
9	CuCl$_2$	TMEDA	82	47	4
10	CuBr$_2$	TMEDA	98	45	14
11	Cu(OAc)$_2$	TMEDA	97	35	36
12	Cu(OPiv)$_2$	TMEDA	40	19	3
13	Cu(OTf)$_2$	TMEDA	98	54	25
14	Cu(OTf)$_2$	--	98	67	8
15	CuOTf	--	98	48	26
16	--	--	23	6	2
17[c]	--	TMEDA	32	3	0
18[d]	Cu(OTf)$_2$	--	95	**71**	6
19[d,e]	Cu(OTf)$_2$	--	91	77	3
20[d,f]	Cu(OTf)$_2$	--	97	**89 (81)**	2
21[d,f]	Fe(OTf)$_2$	--	97	39	8
22[d,f]	Fe(OTf)$_3$	--	93	41	8
23[d,f]	LiOTf	--	65	16	3
24[d,f]	Zn(OTf)$_2$	--	69	18	3

[a]Reaction conditions: **1a** (0.5 mol), **2a** (0.5 mL), DTBP (1.25 mmol), [M] (2 mol%), Ligand (2 mol%) and at 120 °C for 9 hours. [b]The yield was determined by GC with biphenyl as internal standard. [c]2 mol% HOTf was added. [d]0.5 mL EtOAc was added. [e]**2a** (1 mL). [f]**2a** (2 mL). DTBP = *Di*-*tert*-butyl Peroxide

We started our research by applying *N*-methoxybenzamide **1a** and cyclohexene **2a** in a model reaction to test different reaction conditions (Table S1). For the optimization study, we focused our attention on the catalysis precursors. The metal played an important role on the selectivity and yield. When Cu(acac)$_2$ was used, the moderate yield was obtained (Table S1, entry 4). Ni,

Fe, Mn all gave poor yield (Table S1, entry 1-3). Next, the different Copper catalysis precursors were tried. The use of 2 mol% of $Cu(OTf)_2$ and TMEDA gave the more better result (Table S1, entry 13). Other copper catalysis was not able to facilitate the reaction (Table S1, entry 5-12). The selectivity was very poor when $Cu(OAc)_2$ was used, 25% of **5** was got (Table S1, entry 11). To be satisfactory, The applying of only $Cu(OTf)_2$ with on TMEDA increased the yield (Table S1, entry 14) .Maybe, the coordination of the $Cu(OTf)_2$ and TMEDA blocked the role of $Cu(OTf)_2$. But, CuOTf only gave the poor selectivity (Table S1, entry 15). When the $Cu(OTf)_2$ was not added, there was only trace **3aa** obtained (Table S1, entry 16). On the other hand, HOTf instead of $Cu(OTf)_2$ was used, but we only got traceless product. To our exciting, an increasing yield and selectivity was obtained with the **2a** increased (Table S1, entry 18-20). The best yield was got when **2a** was used for 2 mL (Table S1, entry 20). At last, the different metal cations were examined, but the yield was dramatically reduced (Table S1, entry 21-24).

Reusing of cyclohexene 2a

Table S2. Reusing of 2a. [a]

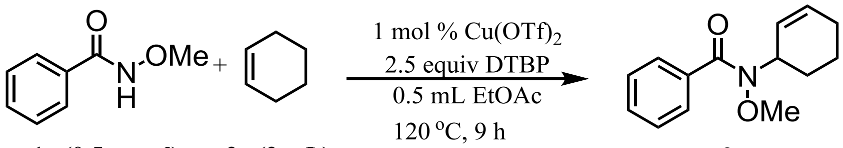

1a (0.5 mmol) **2a (2 mL)** **3aa**

The number of repeat time	$[Cu(OTf)_2]$/DTBP	Yield [%][b]
1	1 mol%/2.5 equiv	90
2	additional 1 mol%/2.5 equiv	64
3	additional 1 mol%/2.5 equiv	52
4	additional 1 mol%/2.5 equiv	45

[a] Reaction conditions: **1a** (0.5 mol), **2a** (0.5 mL), DTBP (1.25 mmol), $[Cu(OTf)_2]$ (1 mol%), at 120 °C for 9 hours.

[b] The yield was determined by GC with biphenyl as internal standard.

General procedure for the C(sp^3)-H/N-H radical oxidative cross-coupling

In an oven-dried tube equipped with a stir bar, $Cu(OTf)_2$ (3.6 mg, 0.01 mmol) and *N*-methoxybenzamide **1a** (75.5 mg, 0.50 mmol) were combined and sealed. The tube was then charged with nitrogen, ethyl acetate (0.5 mL) and cyclohexene **2a** (2.0 mL) were successively injected into the tube by syringe. Under the protection by nitrogen, DTBP (183 mg, 1.25 mmol) was slowly injected into the reaction tube. The reaction was then put into oil bath under 120 ^oC. After stirring for 9 h, the reaction was cooled down to room temperature and quenched with saturated $Na_2S_2O_3$ solution. After extraction with ethyl acetate (3 x 10 mL), the organic layers were combined and dried over anhydrous Na_2SO_4. The pure product was obtained by flash column chromatography on silica gel (petroleum: ethyl acetate = 50:1 - 5:1). The product was isolated as a colourless oil (93.0 mg, 81%)

Detail descriptions for products

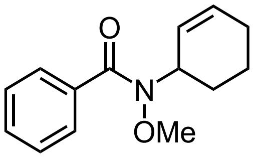

***N*-(Cyclohex-2-enyl)-*N*-Methoxybenzamide (3aa)**, a colorless oil, ^{1}H NMR (400 MHz, $CDCl_3$) δ = 7.65 – 7.59 (m, 2H), 7.49 – 7.34 (m, 3H), 6.00 – 5.88 (m, 1H), 5.71 (dd, *J* = 10.1, 2.2 Hz, 1H), 4.85 (br, 1H), 3.56 (s, 3H), 2.15 – 2.03 (m, 1H), 2.03 – 1.83 (m, 4H), 1.70 – 1.52 (m, 1H). ^{13}C NMR (101 MHz, $CDCl_3$) δ 170.2, 134.7, 131.4, 130.2, 127.9, 127.5, 126.5, 64.1, 55.4, 26.3, 24.2, 20.9. HRMS (EI) calcd for $C_{14}H_{17}NO_2$ $[M]^+$: 231.1259; found: 231.1260.

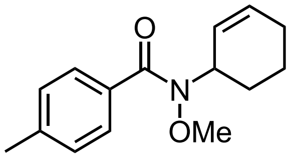

***N*-(Cyclohex-2-enyl)-*N*-Methoxy-4-methylbenzamide (3ba),** a colorless oil, ^{1}H NMR (400 MHz, $CDCl_3$) δ = 7.54 (d, *J* = 8.0 Hz, 2H), 7.20 (d, *J* = 8.0 Hz, 2H), 6.04 – 5.85 (m, 1H), 5.71 (dd, *J* = 10.2, 1.8 Hz, 1H), 4.93 (br, 1H), 3.60 (s, 3H), 2.38 (s, 3H), 2.14 – 2.03 (m, 1H), 2.03 – 1.83 (m, 4H), 1.68 – 1.55 (m, 1H). ^{13}C NMR (101 MHz, $CDCl_3$) δ 170.4, 140.6, 131.8, 131.4, 128.7, 127.8, 126.7, 64.2, 55.6, 26.4, 24.3, 21.3, 21.0. HRMS (EI) calcd for $C_{15}H_{19}NO_2$ $[M]^+$: 245.1416; found: 245.1417.

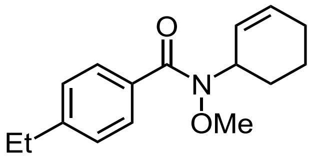

***N*-(Cyclohex-2-enyl)-4-ethyl-*N*-Methoxybenzamide (3ca),** a colorless oil, ^{1}H NMR (400 MHz, $CDCl_3$) δ = 7.61 – 7.52 (m, 2H), 7.23 (d, *J* = 8.4 Hz, 2H), 5.99 – 5.90 (m, 1H), 5.77 – 5.66 (m, 1H), 4.93 (br, 1H), 3.61 (s, 3H), 2.68 (q, *J* = 7.6 Hz, 2H), 2.17 – 2.04 (m, 1H), 2.03 – 1.79 (m, 4H), 1.70 – 1.55 (m, 1H), 1.25 (t, *J* = 7.6 Hz, 3H). ^{13}C NMR (101 MHz, $CDCl_3$) δ 170.4, 146.8, 132.1, 131.4, 127.9, 127.5, 126.8, 64.2, 55.7, 28.7, 26.4, 24.3, 21.1, 15.2. HRMS (EI) calcd for $C_{16}H_{21}NO_2$ $[M]^+$: 259.1572; found: 259.1569.

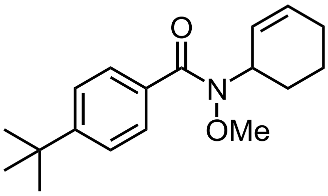

4-*tert*-butyl-*N*-(Cyclohex-2-enyl)-N-Methoxybenzamide (3da), a colorless oil, ^{1}H NMR (400 MHz, $CDCl_3$) δ = 7.69 – 7.50 (m, 2H), 7.49 – 7.34 (m, 2H), 6.04 – 5.85 (m, 1H), 5.72 (dd, *J* = 10.2, 2.0 Hz, 1H), 4.94 (br, 1H), 3.63 (s, 3H), 2.17 – 2.04 (m, 1H), 2.04 – 1.84 (m, 4H), 1.69 – 1.56 (m, 1H), 1.33 (s, 9H). ^{13}C NMR (101 MHz, $CDCl_3$) δ 170.4, 153.7, 131.8, 131.4, 127.6, 126.8, 125.0, 64.2, 55.7, 34.7, 31.1, 26.4, 24.3, 21.1. HRMS (EI) calcd for $C_{18}H_{25}NO_2$ $[M]^+$: 287.1890; found: 287.1890.

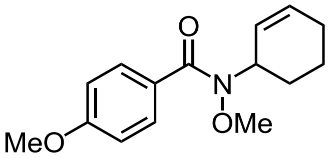

N-(cyclohex-2-enyl)-N,4-dimethoxybenzamide (3ea), a colorless oil, 1H NMR (400 MHz, CDCl3) δ = 7.77 – 7.53 (m, 2H), 6.99 – 6.78 (m, 2H), 6.01 – 5.86 (m, 1H), 5.72 (dd, *J* = 10.2, 2.3, 1H), 4.97 (br, 1H), 3.84 (s, 3H), 3.60 (s, 3H), 2.16 – 2.05 (m, 1H), 2.05 – 1.84 (m, 4H), 1.70 – 1.56 (m, 1H). ^{13}C NMR (101 MHz, $CDCl_3$) δ 169.9, 161.3, 131.4, 130.0, 126.8, 126.8, 113.3, 64.2, 55.7, 55.2, 26.4, 24.3, 21.1. HRMS (EI) calcd for $C_{15}H_{19}NO_3$ $[M]^+$: 261.1365; found: 261.1366.

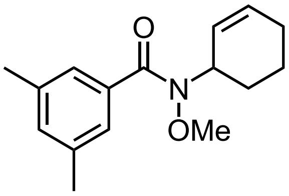

***N*-(Cyclohex-2-enyl)-*N*-Methoxy-3,5-dimethylbenzamide (3fa),** a colorless oil, ^{1}H NMR (400 MHz, $CDCl_3$) δ = 7.20 (s, 2H), 7.07 (s, 1H), 6.02 – 5.85 (m, 1H), 5.81 – 5.59 (m, 1H), 4.88 (br, 1H), 3.63 (s, 3H), 2.34 (s, 6H), 2.16 – 2.03 (m, 1H), 2.03 – 1.84 (m, 4H), 1.69 – 1.53 (m, 1H). ^{13}C NMR (101 MHz, $CDCl_3$) δ 170.9, 137.7, 135.0, 131.9, 131.6, 126.7, 125.2, 64.2, 55.8, 26.5, 24.3, 21.2, 21.1. HRMS (EI) calcd for $C_{16}H_{21}NO_2$ $[M]^+$: 259.1572; found: 259.1568.

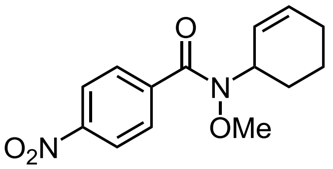

N-(Cyclohex-2-enyl)-N-Methoxy-4-nitrobenzamide (3ga), a colorless oil, ^{1}H NMR (400 MHz, $CDCl_3$) δ = 8.44 – 8.14 (m, 2H), 7.92 – 7.68 (m, 2H), 6.14 – 5.88 (m, 1H), 5.73 (dd, J = 10.2, 2.2 Hz, 1H), 5.06 (br, 1H), 3.55 (s, 3H), 2.20 – 2.09 (m, 1H), 2.09 – 1.87 (m, 4H), 1.76 – 1.62 (m, 1H). ^{13}C NMR (101 MHz, $CDCl_3$) δ 168.1, 148.6, 140.9, 132.2, 128.9, 126.0, 123.3, 64.9, 54.8, 26.5, 24.3, 21.0. HRMS (EI) calcd for $C_{14}H_{16}N_2O_4$ $[M]^+$: 276.1110; found: 276.1113.

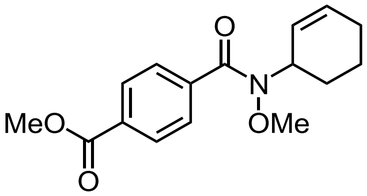

Methyl 4-(cyclohex-2-enyl(methoxy)carbamoyl)benzoate (3ha), a colorless oil, ^{1}H NMR (400 MHz, $CDCl_3$) δ = 8.18 – 8.00 (m, 2H), 7.69 (d, J = 8.3 Hz, 2H), 6.05 – 5.88 (m, 1H), 5.71 (dd, J = 10.1, 1.8 Hz, 1H), 4.98 (br, 1H), 3.94 (s, 3H), 3.56 (s, 3H), 2.16 – 2.07 (m, 1H), 2.06 – 1.85 (m, 4H), 1.72 – 1.57 (m, 1H). ^{13}C NMR (101 MHz, $CDCl_3$) δ 169.4, 166.3, 139.1, 131.8, 131.5, 129.3, 127.6, 126.3, 64.6, 55.1, 52.2, 26.5, 24.3, 21.0. HRMS (EI) calcd for $C_{16}H_{19}NO_4$ $[M]^+$: 289.1314; found: 289.1315.

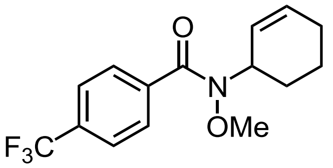

N-(Cyclohex-2-enyl)-N-Methoxy-4-(trifluoromethyl)benzamide (3ia),[2] a colorless oil, ^{1}H NMR (400 MHz, $CDCl_3$) δ = 7.76 (d, J = 8.0, 2H), 7.68 (d, J = 8.0, 2H), 6.05 – 5.92 (m, 1H), 5.78 – 5.67 (m, 1H), 5.01 (br, 1H), 3.56 (s, 3H), 2.19 – 2.07 (m, 1H), 2.07 – 1.86 (m, 4H), 1.75 – 1.60 (m, 1H). ^{13}C NMR (101 MHz, $CDCl_3$) δ = 168.9, 138.4, 132.0 (q, J = 32.9 Hz), 128.2, 126.2, 125.1 (q, J = 4.0 Hz), 123.7 (q, J = 273.4 Hz), 64.7, 55.0, 26.5, 24.3, 21.0. ^{19}F NMR (377 MHz, $CDCl_3$) δ -62.94. HRMS (EI) calcd for $C_{15}H_{16}F_3NO_2$ $[M]^+$: 299.1133; found: 299.1138.

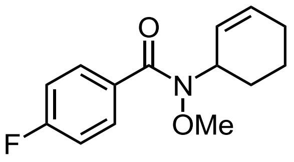

***N*-(Cyclohex-2-enyl)-4-fluoro-*N*-Methoxybenzamide (3ja),** a colorless oil, 1H NMR (400 MHz, CDCl3) δ = 7.80 – 7.59 (m, 2H), 7.17 – 7.01 (m, 2H), 6.01 – 5.91 (m, 1H), 5.75 – 5.67 (m, 1H), 5.00 (br, 1H), 3.56 (s, 3H), 2.18 – 2.05 (m, 1H), 2.05 – 1.85 (m, 4H), 1.72 – 1.58 (m, 1H). ^{13}C NMR (101 MHz, $CDCl_3$) δ = 169.2, 163.8 (d, *J* = 251.6 Hz), 131.7, 130.8 (d, *J* = 3.4 Hz), 130.4 (d, *J* = 8.6 Hz), 126.6, 115.2 (d, *J* = 21.8 Hz), 64.5, 55.3, 26.5, 24.4, 21.1. ^{19}F NMR (377 MHz, $CDCl_3$) δ -109.18. HRMS (EI) calcd for $C_{14}H_{16}FNO_2$ $[M]^+$: 249.1165; found: 249.1160.

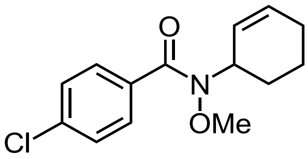

4-chloro-*N*-(Cyclohex-2-enyl)-*N*-Methoxybenzamide (3ka), a colorless oil, ^{1}H NMR (400 MHz, $CDCl_3$) δ = 7.80 – 7.59 (m, 2H), 7.17 – 7.01 (m, 2H), 6.01 – 5.91 (m, 1H), 5.75 – 5.67 (m, 1H), 5.00 (s, 1H), 3.56 (s, 3H), 2.18 – 2.05 (m, 1H), 2.05 – 1.85 (m, 4H), 1.72 – 1.58 (m, 1H). ^{13}C NMR (101 MHz, $CDCl_3$) δ 169.1, 136.5, 133.1, 131.8, 129.4, 128.3, 126.4, 64.5, 55.2, 26.5, 24.3, 21.0. HRMS (EI) calcd for $C_{14}H_{16}ClNO_2$ $[M]^+$: 265.0870; found: 265.0867.

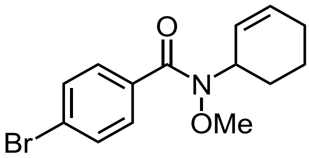

4-bromo-*N*-(Cyclohex-2-enyl)-*N*-Methoxybenzamide (**3la**), a colorless oil, 1H NMR (400 MHz, CDCl3) δ = 7.66 – 7.46 (m, 4H), 6.04 – 5.87 (m, 1H), 5.75 – 5.65 (m, 1H), 4.99 (s, 1H), 3.57 (s, 3H), 2.18 – 2.06 (m, 1H), 2.05 – 1.85 (m, 4H), 1.72 – 1.59 (m, 1H). ^{13}C NMR (101 MHz, $CDCl_3$) δ 169.1, 133.6, 131.7, 131.3, 129.6, 126.4, 124.8, 64.5, 55.1, 26.4, 24.3, 21.0. HRMS (EI) calcd for $C_{14}H_{16}BrNO_2$ $[M]^+$: 309.0364; found: 309.0360.

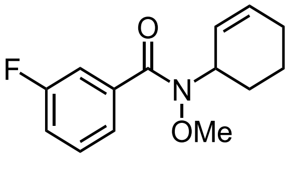

N-(Cyclohex-2-enyl)-3-fluoro-N-Methoxybenzamide (3ma), a colorless oil, ^{1}H NMR (400 MHz, $CDCl_3$) δ = 7.48 – 7.32 (m, 3H), 7.18 – 7.12 (m, 1H), 6.07 – 5.85 (m, 1H), 5.71 (dd, *J* = 10.1, 2.3 Hz, 1H), 4.97 (br, 1H), 3.59 (s, 3H), 2.17 – 2.06 (m, 1H), 2.06 – 1.86 (m, 4H), 1.72 – 1.58 (m, 1H). ^{13}C NMR (101 MHz, $CDCl_3$) δ = 168.8 (d, *J* = 2.4 Hz), 162.1 (d, *J* = 248.0 Hz), 136.8 (d, *J* = 7.1 Hz), 131.8, 129.8 (d, *J* = 8.0 Hz), 126.3, 123.6 (d, *J* = 3.1 Hz), 117.3 (d, *J* = 21.2 Hz), 115.0 (d, *J* = 23.2 Hz), 64.5, 55.3, 26.5, 24.3, 21.0. ^{19}F NMR (377 MHz, $CDCl_3$) δ -112.45. HRMS (EI) calcd for $C_{14}H_{16}FNO_2$ $[M]^+$: 249.1165; found: 249.1168.

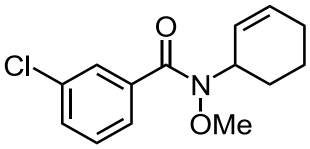

4-chloro-N-(Cyclohex-2-enyl)-N-Methoxybenzamide (3na), a colorless oil, ^{1}H NMR (400 MHz, $CDCl_3$) δ = 7.63 (s, 1H), 7.53 (dd, *J* = 7.6, 1.1 Hz, 1H), 7.46 – 7.39 (m, 1H), 7.35 (t, *J* = 7.8 Hz, 1H), 6.02 – 5.91 (m, 1H), 5.71 (d, J=10.1, 1H), 4.98 (br, 1H), 3.58 (s, 3H), 2.18 – 2.06 (m, 1H), 2.06 – 1.84 (m, 4H), 1.72 – 1.58 (m, 1H). ^{13}C NMR (101 MHz, $CDCl_3$) δ 168.7, 136.5, 134.1, 131.9, 130.4, 129.5, 128.0, 126.3, 126.0, 64.6, 55.2, 26.5, 24.3, 21.0. HRMS (EI) calcd for $C_{14}H_{16}ClNO_2$ $[M]^+$: 265.0870; found: 265.0868.

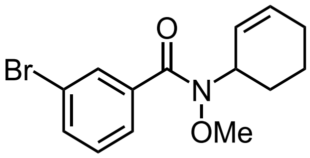

3-bromo-N-(Cyclohex-2-enyl)-N-Methoxybenzamide (3oa), a colorless oil, 1H NMR (400 MHz, CDCl3) δ = 7.78 (t, *J* = 1.7 Hz, 1H), 7.66 – 7.44 (m, 2H), 7.32 – 7.24 (m, 1H), 6.05 – 5.83 (m, 1H), 5.76 – 5.63 (m, 1H), 4.97 (br, 1H), 3.57 (s, 3H), 2.16 – 2.05 (m, 1H), 2.05 – 1.84 (m, 4H), 1.78 – 1.51 (m, 1H). 13C NMR (101 MHz, CDCl3) δ 168.5, 136.7, 133.2, 131.8, 130.7, 129.6, 126.3, 126.2, 122.0, 64.5, 55.1, 26.4, 24.3, 20.9. HRMS (EI) calcd for $C_{14}H_{16}BrNO_2$ $[M]^+$: 309.0364; found: 309.0362.

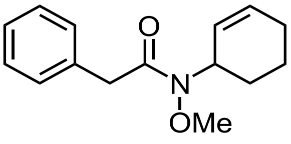

N-(Cyclohex-2-enyl)-N-Methoxy-2-phenylacetamide (3pa), a colorless oil, ^{1}H NMR (400 MHz, CDCl3) δ = 7.35 – 7.26 (m, 4H), 7.26 – 7.21 (m, 1H), 5.93 – 5.84 (m, 1H), 5.59 (d, *J*

= 9.6, 1H), 5.04 (br, 1H), 3.85 – 3.74 (m, 2H), 3.72 (s, 3H), 2.14 – 1.90 (m, 2H), 1.87 – 1.76 (m, 3H), 1.69 – 1.55 (m, 1H).^{13}C NMR (101 MHz, $CDCl_3$) δ 173.6, 134.8, 131.1, 129.16, 128.4, 127.0, 126.6, 64.9, 53.7, 39.9, 26.2, 24.4, 21.1. HRMS (EI) calcd for $C_{15}H_{19}NO_2$ $[M]^+$: 245.1416; found: 245.1420.

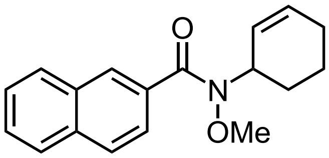

***N*-(Cyclohex-2-enyl)-*N*-Methoxy-2-naphthamide (3qa),** a colorless oil, 1H NMR (400 MHz, CDCl3) δ = 8.18 (s, 1H), 7.95 – 7.81 (m, 3H), 7.70 (dd, *J* = 8.5, 1.5 Hz, 1H), 7.60 – 7.44 (m, 2H), 6.04 – 5.87 (m, 1H), 5.76 (d, *J* = 10.1 Hz, 1H), 5.01 (br, 1H), 3.60 (s, 3H), 2.16 – 1.83 (m, 5H), 1.69 – 1.54 (m, 1H). ^{13}C NMR (101 MHz, $CDCl_3$) δ 170.3, 134.0, 132.4, 132.1, 131.6, 128.6, 128.0, 127.7, 127.6, 127.2, 126.6, 126.4, 124.6, 64.4, 55.6, 26.5, 24.3, 21.03. HRMS (EI) calcd for $C_{18}H_{19}NO_2$ [M]+: 281.1416; found: 281.1411.

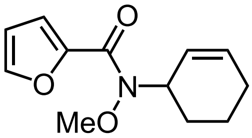

***N*-(Cyclohex-2-enyl)-*N*-Methoxyfuran-2-carboxamide (3ra),** a colorless oil,1H NMR (400 MHz, CDCl3) δ = 7.67 – 7.54 (m, 1H), 7.15 (d, *J* = 3.5 Hz, 1H), 6.52 (dd, *J* = 3.5, 1.7 Hz, 1H), 6.08 – 5.86 (m, 1H), 5.71 (dd, *J* = 10.2, 1.9 Hz, 1H), 5.30 – 5.10 (m, 1H), 3.81 (s, 3H), 2.17 – 2.07 (m, 1H), 2.07 – 1.85 (m, 4H), 1.75 – 1.63 (m, 1H). ^{13}C NMR (101 MHz, $CDCl_3$) δ 159.8, 146.0, 145.2, 131.5, 126.6, 117.4, 111.5, 65.0, 54.3, 26.2, 24.4, 21.0. HRMS (EI) calcd for $C_{12}H_{15}NO_3$ $[M]^+$: 221.1052; found: 221.1049.

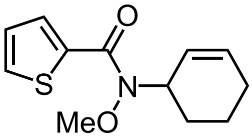

***N*-(Cyclohex-2-enyl)-*N*-Methoxythiophene-2-carboxamide (3sa),** a colorless oil, ^{1}H NMR (400 MHz, $CDCl_3$) δ = 7.96 (dd, *J* = 3.8, 1.3 Hz, 1H), 7.56 (dd, J = 5.1, 1.3 Hz, 1H), 7.10 (dd, *J* = 5.0, 3.9 Hz, 1H), 6.01 – 5.91 (m, 1H), 5.74 (dd, *J* = 10.1, 2.2 Hz, 1H), 5.27 – 5.17 (m, 1H), 3.81 (s, 3H), 2.12 (m, 1H), 2.07 – 1.85 (m, 4H), 1.77 – 1.63 (m, 1H). ^{13}C NMR (101 MHz, $CDCl_3$) δ 163.0, 134.4, 133.8, 132.2, 131.3, 126.8, 126.7, 65.3, 54.6, 26.3, 24.4, 21.08. HRMS (EI) calcd for $C_{12}H_{15}NO_2S$ $[M]^+$: 237.0823; found: 237.0821.

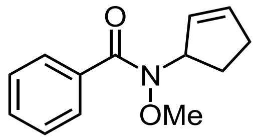

***N*-(Cyclopent-2-enyl)-*N*-Methoxybenzamide (3ab),** a colorless oil, ^{1}H NMR (400 MHz, $CDCl_3$) δ = 7.70 – 7.53 (m, 2H), 7.52 – 7.34 (m, 3H), 6.07 (ddd, *J* = 6.5, 4.3, 2.2 Hz, 1H), 5.87 – 5.67 (m, 1H), 5.45 (br, 1H), 3.56 (s, 3H), 2.65 – 2.51 (m, 1H), 2.42 – 2.25 (m, 1H), 2.24 – 2.14 (m, 1H), 2.14 – 2.04 (m, 1H). ^{13}C NMR (101 MHz, $CDCl_3$) δ 169.7, 136.2, 134.9, 130.3, 128.4, 128.03, 127.7, 65.0, 64.4, 31.6, 27.0. HRMS (EI) calcd for $C_{13}H_{15}NO_2$ $[M]^+$: 217.1103; found: 217.1104.

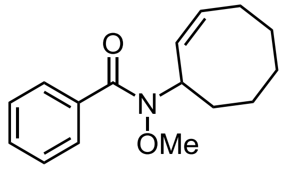

(*Z*)-*N*-(Cyclooct-2-enyl)-*N*-Methoxybenzamide (3ac), a colorless oil, ^{1}H NMR (400 MHz, $CDCl_3$) δ = 7.65 – 7.53 (m, 2H), 7.47 – 7.37 (m, 3H), 5.90 – 5.63 (m, 2H), 5.05 (s, 1H), 3.70 (s, 3H), 2.05 – 1.92 (m, 2H), 1.90 – 1.75 (m, 2H), 1.69 – 1.52 (m, 3H), 1.51 – 1.40 (m, 1H), 1.32 – 1.19 (m, 2H). ^{13}C NMR (101 MHz, $CDCl_3$) δ 170.29, 135.1, 130.5, 130.3, 128.1, 127.5, 127.2, 64.1, 58.2, 33.2, 28.9, 26.3, 24.2. HRMS (EI) calcd for $C_{16}H_{21}NO_2$ $[M]^+$: 259.1572; found: 259.1576.

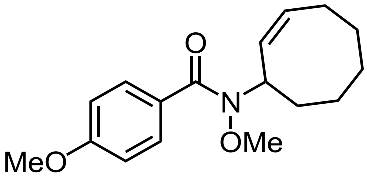

(*Z*)-*N*-(Cyclooct-2-enyl)-*N*,4-dimethoxybenzamide (3ad), a colorless oil, ^{1}H NMR (400 MHz, $CDCl_3$) δ = 7.73 – 7.53 (m, 2H), 6.99 – 6.80 (m, 2H), 5.89 – 5.64 (m, 2H), 5.15 – 5.06 (m, 1H), 3.84 (s, 3H), 3.69 (s, 3H), 2.08 – 1.90 (m, 3H), 1.86 – 1.76 (m, 1H), 1.70 – 1.53 (m, 3H), 1.53 – 1.41 (m, 1H), 1.36 – 1.22 (m, 2H). ^{13}C NMR (101 MHz, $CDCl_3$) δ 170.0, 161.3, 130.3, 129.8, 127.4, 127.0, 113.3, 64.0, 58.4, 55.2, 33.3, 28.9, 26.3, 26.3, 24.3. HRMS (EI) calcd for $C_{17}H_{23}NO_3$ $[M]^+$: 289.1683; found: 289.1682.

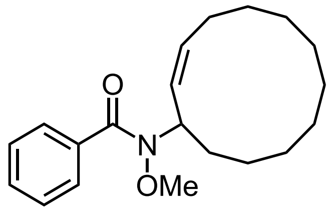

(*Z*)-*N*-(Cyclododec-2-enyl)-*N*-Methoxybenzamide (3ae), a colorless oil, a mixture of R and S. ^{1}H NMR (400 MHz, $CDCl_3$) δ = 7.74 – 7.53 (m, 2H), 7.51 – 7.32 (m, 3H), 5.72 – 5.53 (m, 2H), 5.45 (td, *J* = 11.4, 4.5 Hz, 1H), 5.12 (s, 1H), 4.56 (s, 1H), 3.71 – 3.55 (m, 3H), 2.32 – 2.07 (m, 1H), 2.05 – 1.69 (m, 3H), 1.63 – 1.05 (m, 14H). ^{13}C NMR (101 MHz, $CDCl_3$) δ 170.2, 167.0, 135.8, 135.1, 134.9, 132.8, 130.4, 130.3, 128.0, 128.0, 127.9, 127.8, 127.7, 127.15, 64.2, 64.0, 63.1, 54.2, 32.0, 30.4, 29.8, 26.6, 26.0, 25.8, 25.2, 25.1, 24.6, 24.4, 24.3, 24.1, 23.7, 23.3, 23.1, 22.7, 22.1, 21.8. HRMS (EI) calcd for $C_{20}H_{29}NO_2$ $[M-OMe]^+$: 284.2014; found: 284.2018.

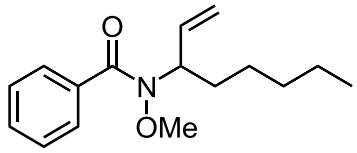

***N*-Methoxy-*N*-(Oct-1-en-3-yl)benzamide (3af),**[3] a colorless oil, ^{1}H NMR (400 MHz, $CDCl_3$) δ = 7.69 – 7.53 (m, 2H), 7.51 – 7.33 (m, 3H), 6.00 (ddd, *J* = 17.4, 10.5, 7.1 Hz, 1H), 5.32 – 5.11 (m, 2H), 4.64 (br, 1H), 3.63 (s, 3H), 1.96 – 1.81 (m, 1H), 1.72 – 1.57 (m, 1H), 1.43 – 1.20 (m, 6H), 0.87 (t, J=6.9 Hz, 3H). ^{13}C NMR (101 MHz, $CDCl_3$) δ 170.5, 136.3, 135.0, 130.4, 128.1, 127.8, 117.2, 64.1, 62.2, 31.5, 31.2, 25.7, 22.5, 14.0. HRMS (EI) calcd for $C_{16}H_{23}NO_2$ $[M]^+$: 261.1729; found: 261.1723.

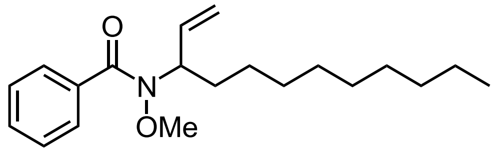

***N*-(Dodec-1-en-3-yl)-*N*-Methoxybenzamide (3ag),** a colorless oil, ^{1}H NMR (400 MHz, $CDCl_3$) δ = 7.70 – 7.53 (m, 2H), 7.50 – 7.32 (m, 3H), 6.00 (ddd, *J* = 17.5, 10.5, 7.1 Hz, 1H), 5.28 – 5.14 (m, 2H), 4.63 (br, 1H), 3.64 (s, 3H), 1.97 – 1.83 (m, 1H), 1.74 – 1.57 (m, 1H), 1.43 – 1.16 (m, 14H), 0.88 (t, *J* = 6.9 Hz, 3H). ^{13}C NMR (101 MHz, $CDCl_3$) δ 170.6, 136.4, 135.0, 130.5, 128.2, 127.9, 117.3, 64.1, 62.0, 31.9, 31.4, 29.6, 29.5, 29.4, 29.3, 26.2, 22.7, 14.2. HRMS (EI) calcd for $C_{20}H_{31}NO_2$ $[M]^+$: 317.2355; found: 317.2351.

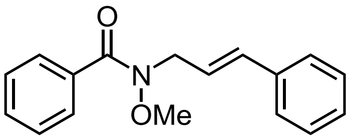

***N*-Cinnamyl-*N*-Methoxybenzamide (3ah),[4]** a colorless oil, ^{1}H NMR (400 MHz, $CDCl_3$) δ = 7.77 – 7.64 (m, 2H), 7.49 – 7.43 (m, 1H), 7.43 – 7.36 (m, 4H), 7.36 – 7.28 (m, 2H), 7.28 – 7.21 (m, 1H), 6.64 (d, *J* = 15.9 Hz, 1H), 6.33 (dt, J=15.8, 6.4, 1H), 4.49 (d, J = 6.4 Hz, 2H), 3.59 (s, 3H). ^{13}C NMR (101 MHz, $CDCl_3$) δ 169.7, 136.3, 134.1, 133.7, 130.6, 128.5, 128.1, 128.0, 127.8, 126.4, 123.2, 62.1, 49.5. HRMS (EI) calcd for $C_{17}H_{17}NO_2$ $[M]^+$: 267.1264; found: 267.1262.

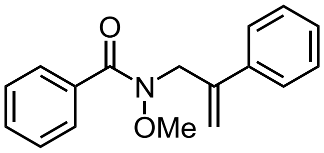

***N*-Methoxy-*N*-(2-phenylallyl)benzamide (3ai),** a colorless oil, ^{1}H NMR (400 MHz, $CDCl_3$) δ = 7.52 (dd, *J* = 5.2, 3.3 Hz, 2H), 7.50 – 7.45 (m, 2H), 7.44 – 7.38 (m, 1H), 7.38 – 7.29 (m, 5H), 5.57 (s, 1H), 5.40 (s, 1H), 4.83 (s, 2H), 3.45 (s, 3H). ^{13}C NMR (101 MHz, $CDCl_3$) δ 169.7, 142.8, 138.2, 134.0, 130.5, 128.4, 128.0, 127.9, 127.9, 126.2, 115.5, 61.8, 49.8. HRMS (EI) calcd for $C_{17}H_{17}NO_2$ $[M]^+$: 267.1259; found: 267.1256.

References

1. Zhou, L. *et al.* Transition-Metal-Assisted Radical/Radical Cross-Coupling: A New Strategy to the Oxidative C(sp^3)-H/N-H Cross-Coupling. *Org. Lett.* **16**, 3404-3407 (2014).
2. Nielsen, D. U., Taaning, R. H., Lindhardt, A. T., Gogsig, T. M. & Skrydstrup, T. Palladium-Catalyzed Approach to Primary Amides Using Nongaseous Precursors. *Org. Lett.* **13**, 4454-4457 (2011).
3. Castagnolo, D., Armaroli, S., Corelli, F. & Botta, M. Enantioselective synthesis of 1-aryl-2-propenylamines: a new approach to a stereoselective synthesis of the Taxol side chain. *Tetrahedron: Asymmetry* **15**, 941-949 (2004).
4. Miyabe, H., Matsumura, A., Moriyama, K. & Takemoto, Y. Utility of the Iridium Complex of the Pybox Ligand in Regio- and Enantioselective Allylic Substitution. *Org. Lett.* **6**, 4631-4634 (2004).

Copies of product ^{1}H NMR, ^{13}C NMR and ^{19}F NMR

3aa

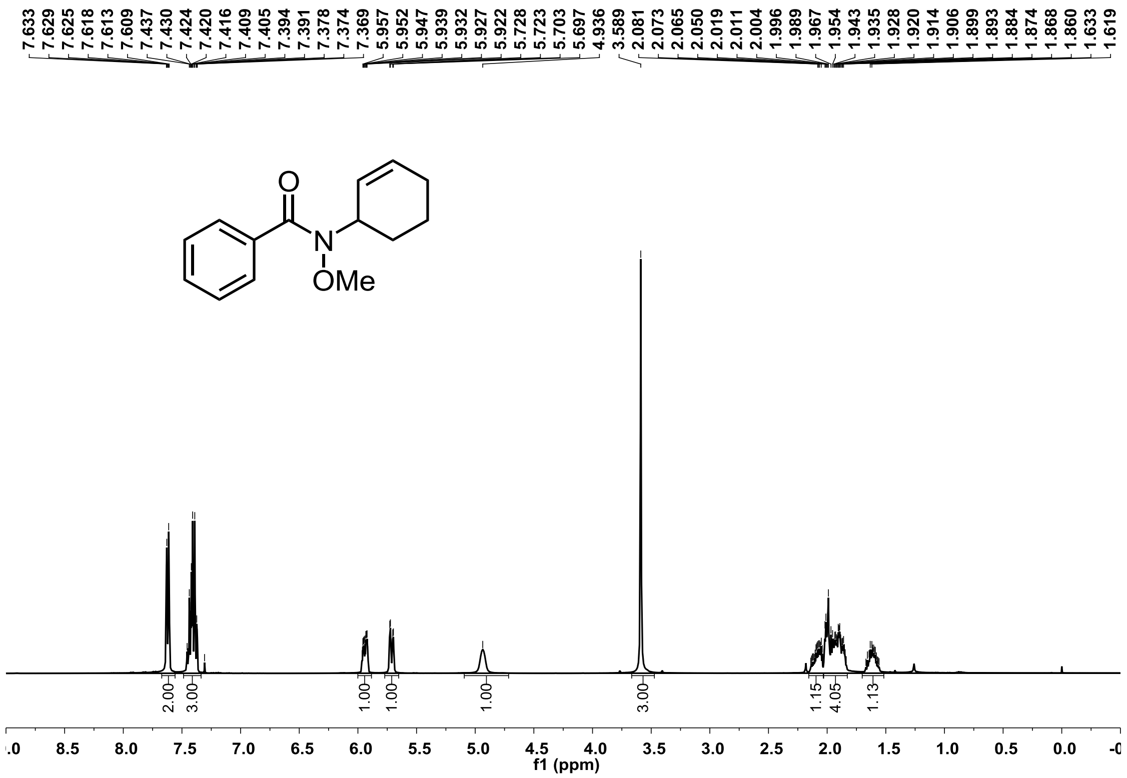

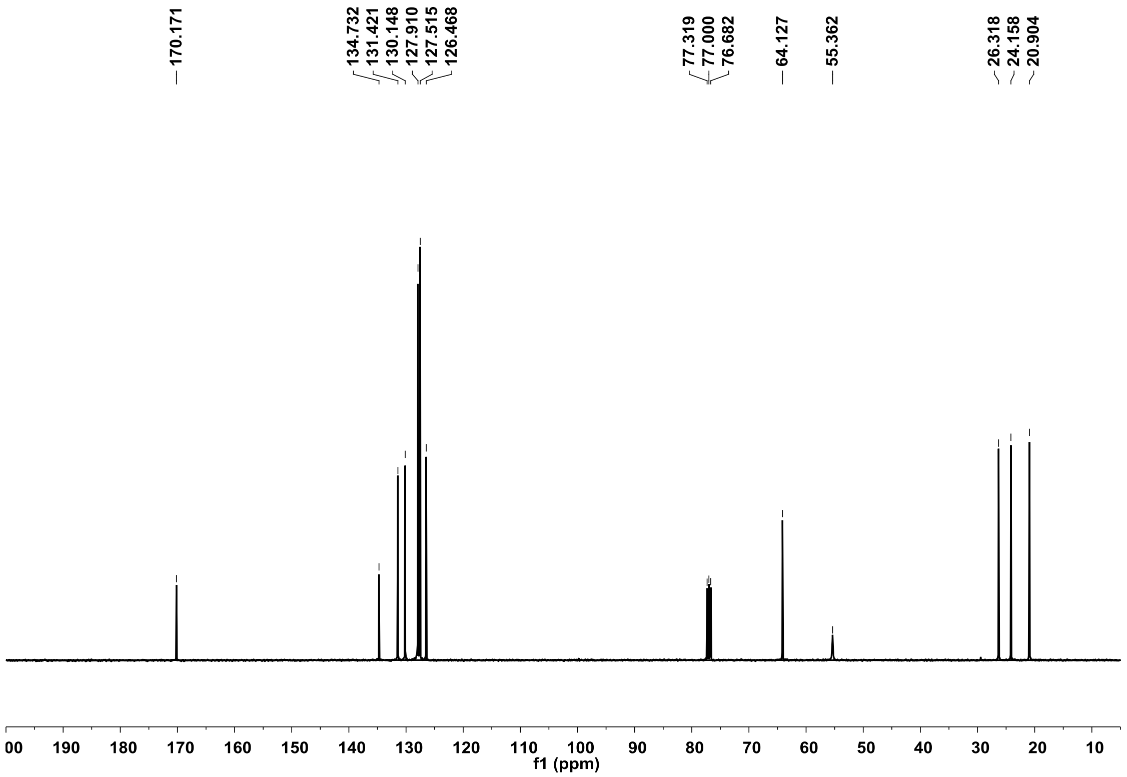

3ba

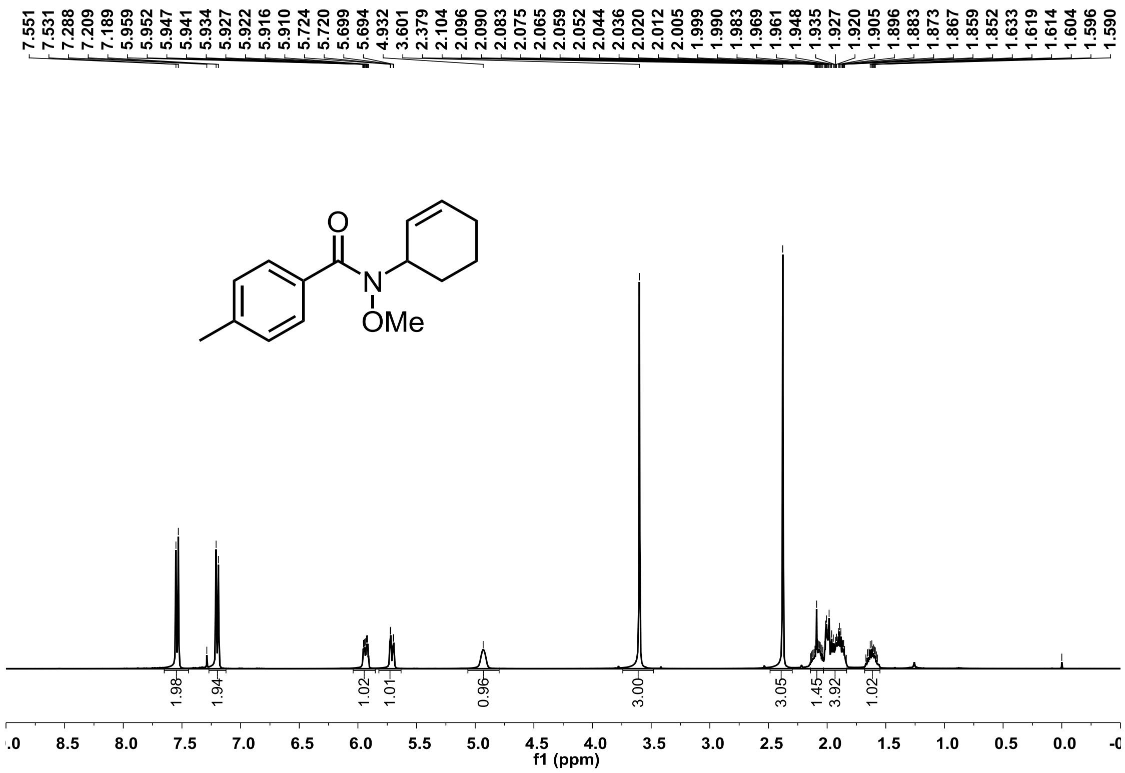
7.551 7.531 7.288 7.209 7.189 5.959 5.952 5.947 5.941 5.934 5.927 5.922 5.916 5.910 5.724 5.720 5.699 5.694 4.932 3.601 2.379 2.104 2.096 2.090 2.083 2.075 2.065 2.059 2.052 2.044 2.036 2.020 2.012 2.005 1.999 1.990 1.983 1.969 1.961 1.948 1.935 1.927 1.920 1.905 1.896 1.883 1.873 1.867 1.859 1.852 1.633 1.619 1.614 1.604 1.596 1.590
O
N
OMe
1.98 1.94 1.02 1.01 0.96 3.00 3.05 1.45 3.92 1.02
8.5 8.0 7.5 7.0 6.5 6.0 5.5 5.0 4.5 4.0 3.5 3.0 2.5 2.0 1.5 1.0 0.5 0.0
f1 (ppm)

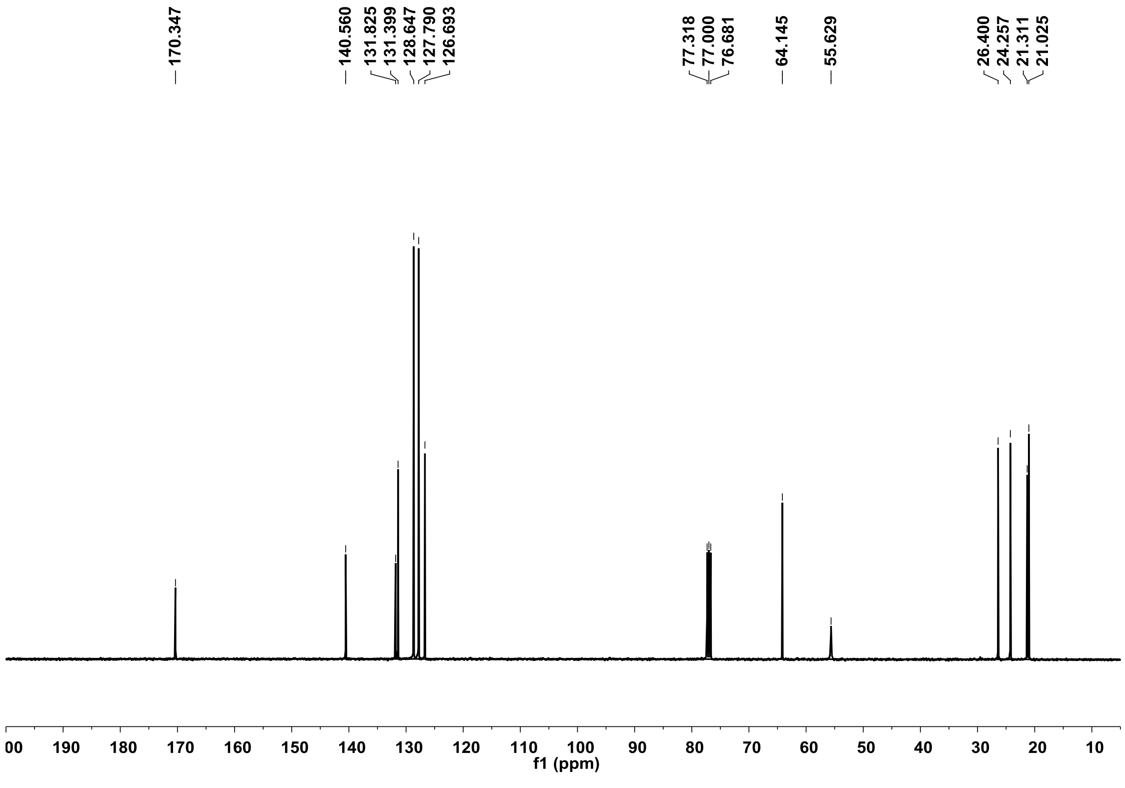
170.347 140.560 131.825 131.399 128.647 127.790 126.693 77.318 77.000 76.681 64.145 55.629 26.400 24.257 21.311 21.025
190 180 170 160 150 140 130 120 110 100 90 80 70 60 50 40 30 20 10
f1 (ppm)

3ca

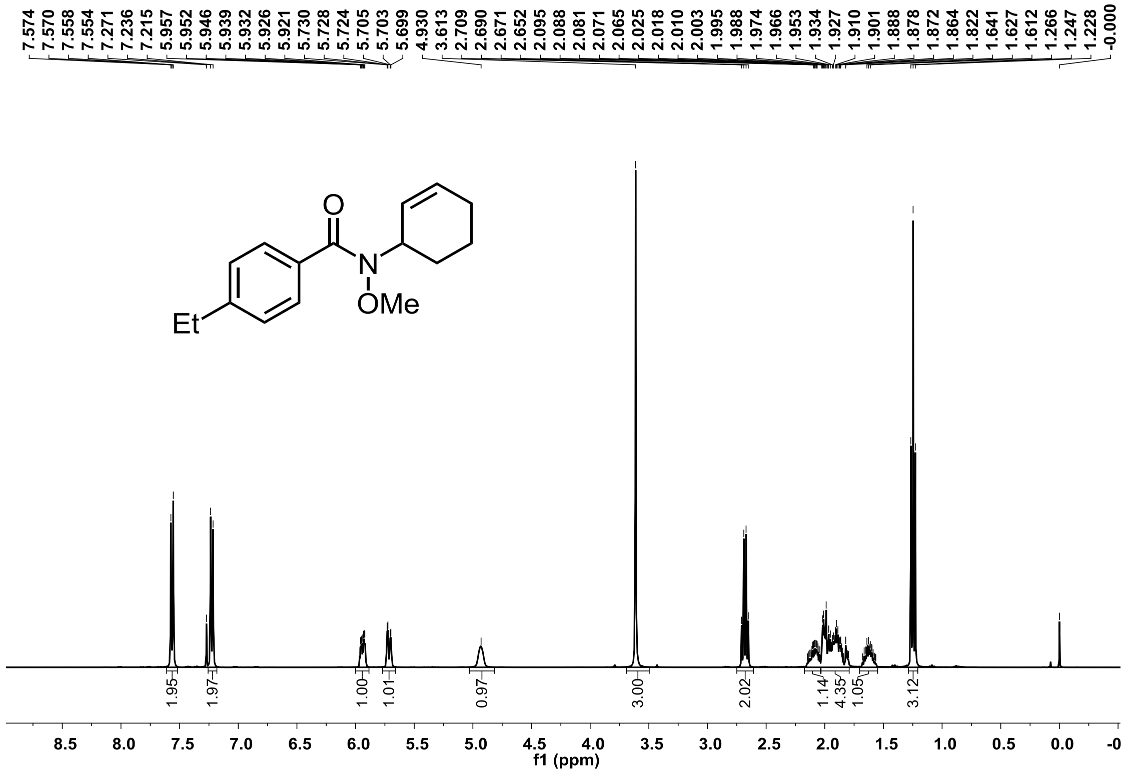
7.574
7.570
7.558
7.554
7.271
7.236
7.215
5.957
5.952
5.946
5.939
5.932
5.926
5.921
5.730
5.728
5.724
5.705
5.703
5.699
4.930
3.613
2.709
2.690
2.671
2.652
2.095
2.088
2.081
2.071
2.065
2.025
2.018
2.010
2.003
1.995
1.988
1.974
1.966
1.953
1.934
1.927
1.910
1.901
1.888
1.878
1.872
1.864
1.822
1.641
1.627
1.612
1.266
1.247
1.228
-0.000
O
N
OMe
Et
1.95
1.97
1.00
1.01
0.97
3.00
2.02
1.14
4.35
1.05
3.12
f1 (ppm)

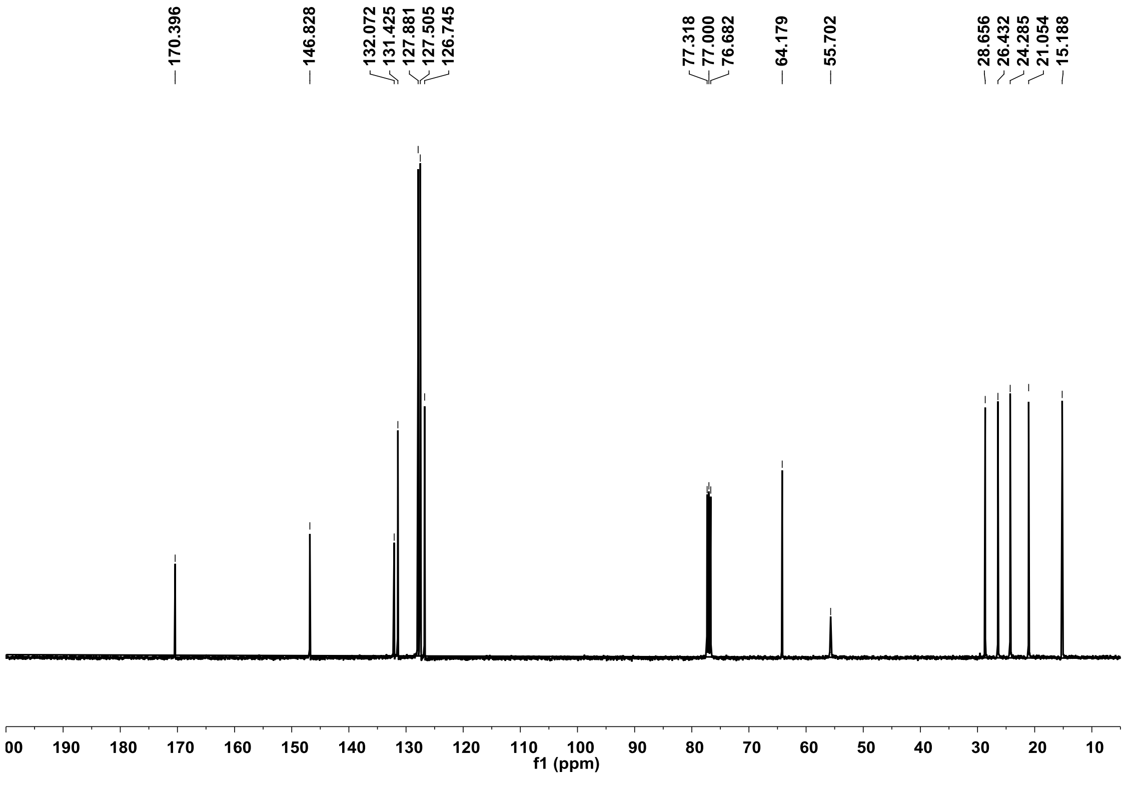
170.396
146.828
132.072
131.425
127.881
127.505
126.745
77.318
77.000
76.682
64.179
55.702
28.656
26.432
24.285
21.054
15.188
f1 (ppm)

3da

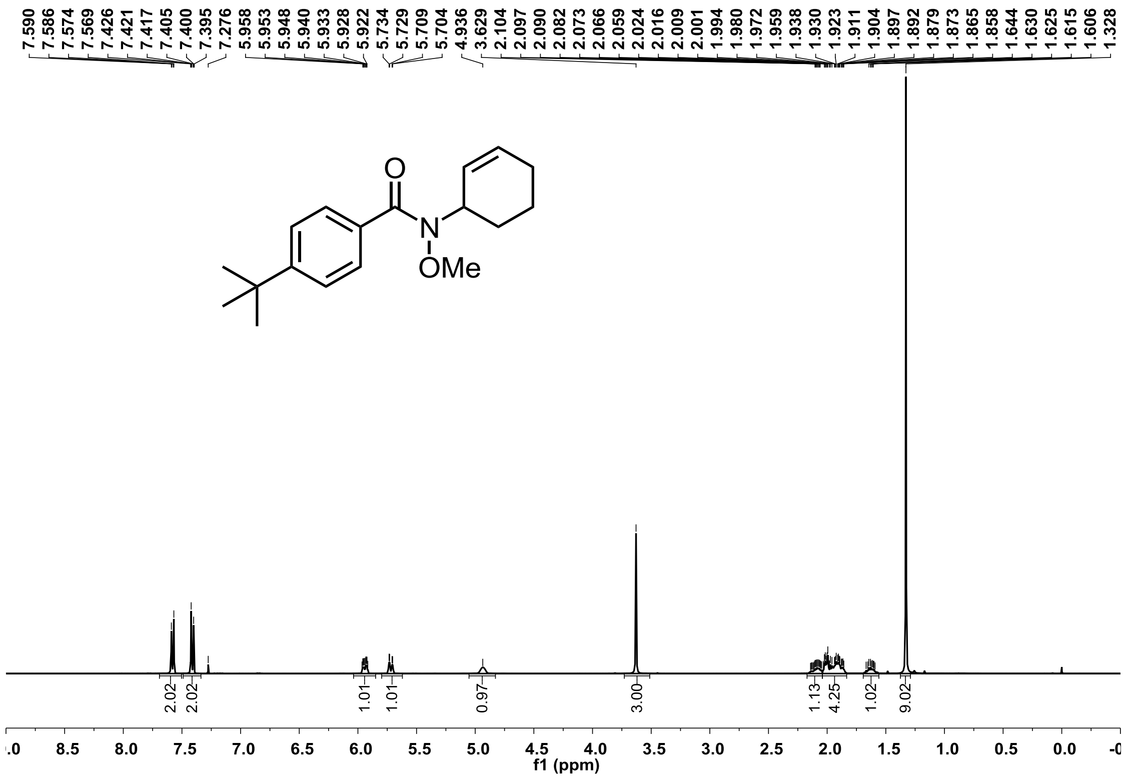

7.590 7.586 7.574 7.569 7.426 7.421 7.417 7.405 7.400 7.395 7.276 5.958 5.953 5.948 5.940 5.933 5.928 5.922 5.734 5.729 5.709 5.704 4.936 3.629 2.104 2.097 2.090 2.082 2.073 2.066 2.059 2.024 2.016 2.009 2.001 1.994 1.980 1.972 1.959 1.938 1.930 1.923 1.911 1.904 1.897 1.892 1.879 1.873 1.865 1.858 1.644 1.630 1.625 1.615 1.606 1.328
O
N
OMe
2.02 2.02 1.01 1.01 0.97 3.00 1.13 4.25 1.02 9.02
f1 (ppm)

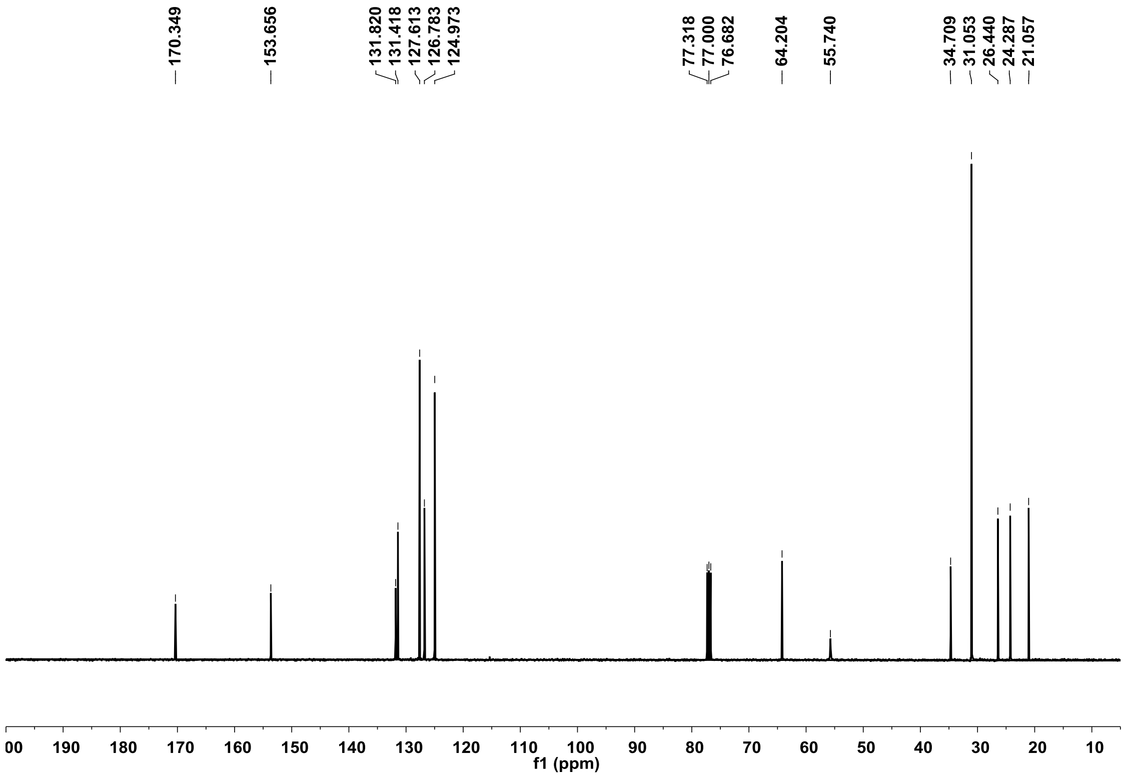

170.349 153.656 131.820 131.418 127.613 126.783 124.973 77.318 77.000 76.682 64.204 55.740 34.709 31.053 26.440 24.287 21.057
f1 (ppm)

3ea

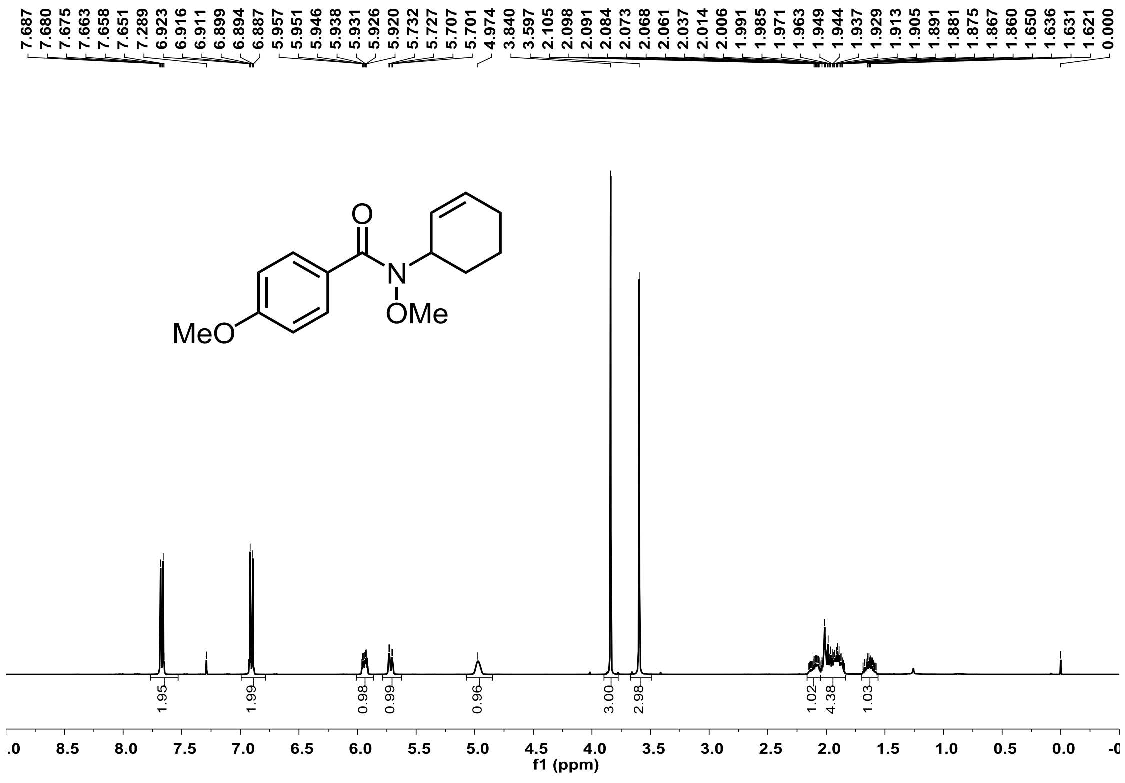

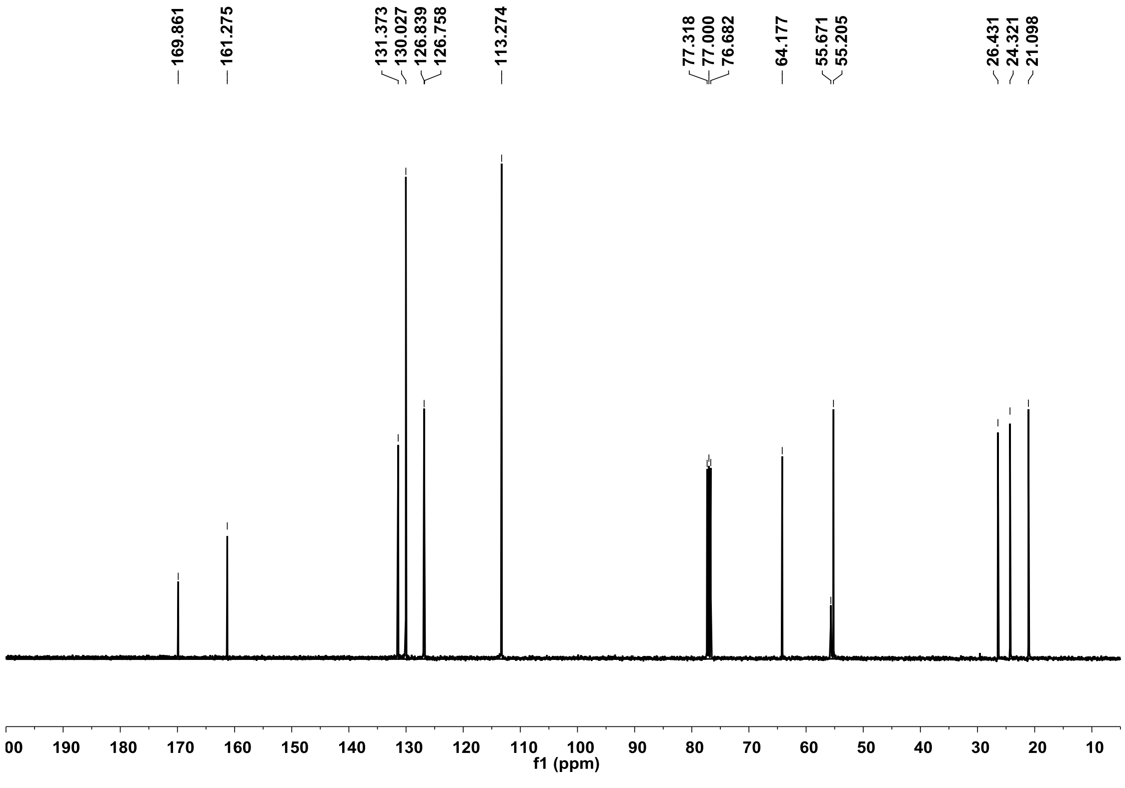

3fa

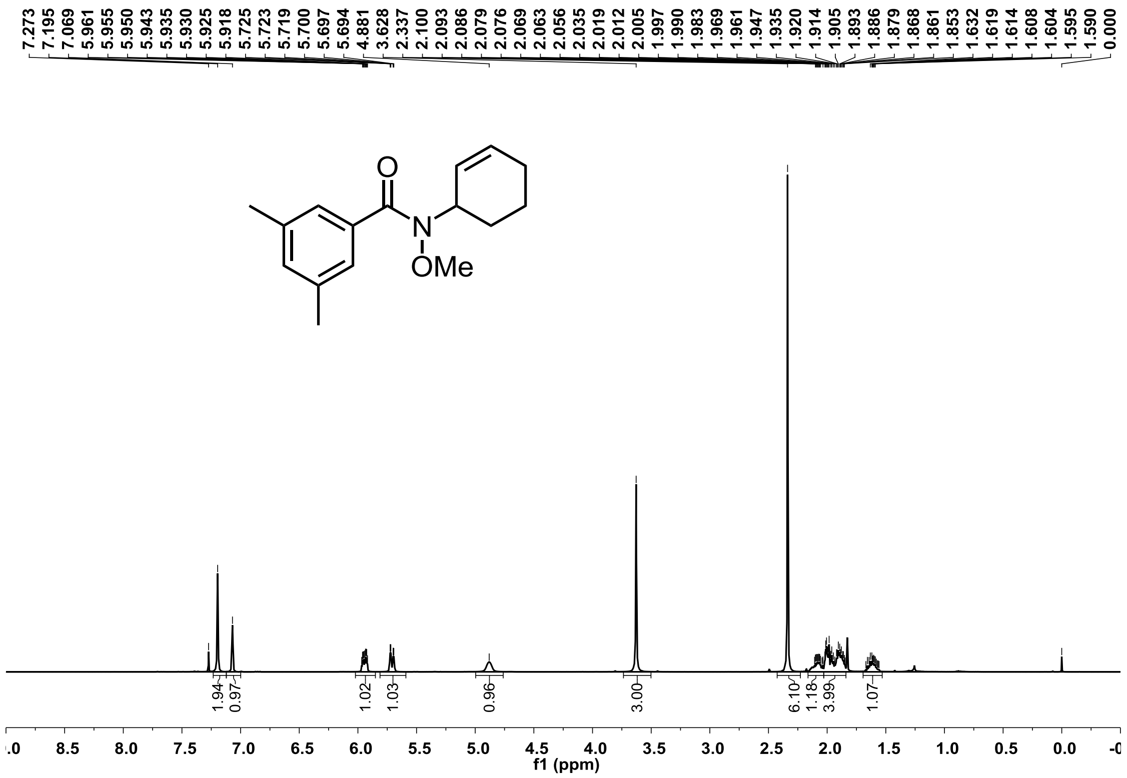
7.273 7.195 7.069 5.961 5.955 5.950 5.943 5.935 5.930 5.925 5.918 5.725 5.723 5.719 5.700 5.697 5.694 4.881 3.628 2.337 2.100 2.093 2.086 2.079 2.076 2.069 2.063 2.056 2.035 2.019 2.012 2.005 1.997 1.990 1.983 1.969 1.961 1.947 1.935 1.920 1.914 1.905 1.893 1.886 1.879 1.868 1.861 1.853 1.632 1.619 1.614 1.608 1.604 1.595 1.590 0.000
O
N
OMe
1.94 0.97 1.02 1.03 0.96 3.00 6.10 1.18 3.99 1.07
8.5 8.0 7.5 7.0 6.5 6.0 5.5 5.0 4.5 4.0 3.5 3.0 2.5 2.0 1.5 1.0 0.5 0.0
f1 (ppm)

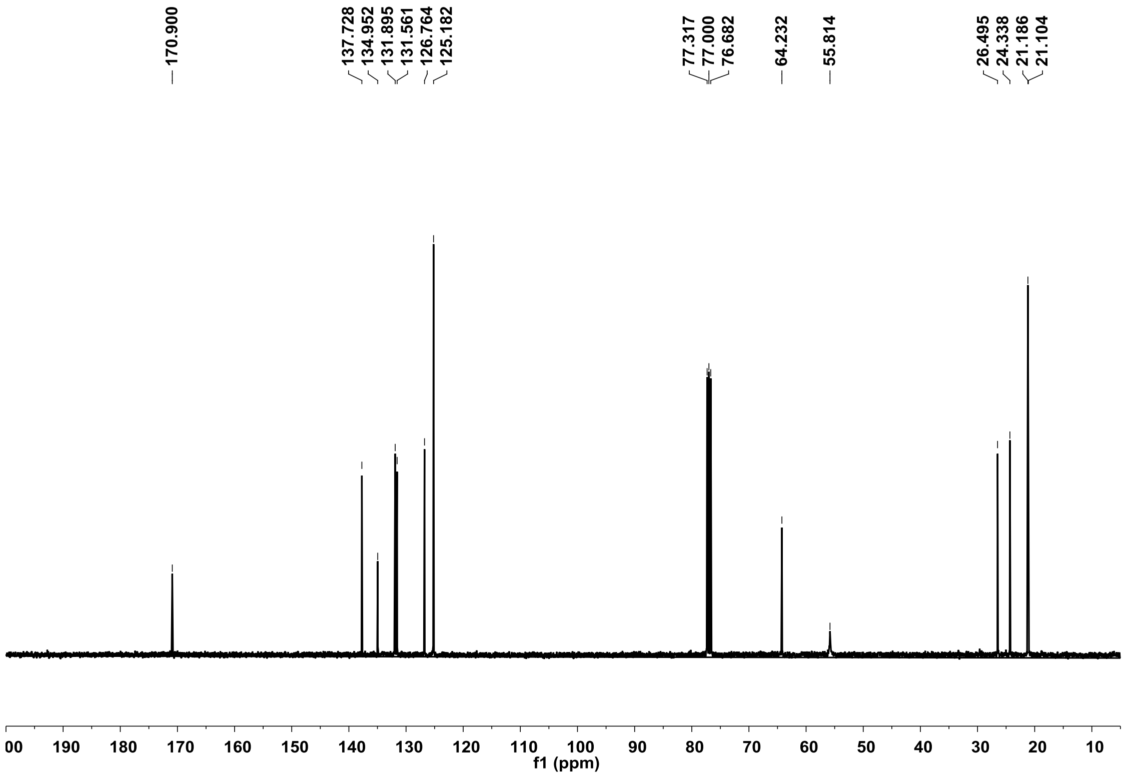
170.900
137.728 134.952 131.895 131.561 126.764 125.182
77.317 77.000 76.682
64.232
55.814
26.495 24.338 21.186 21.104
190 180 170 160 150 140 130 120 110 100 90 80 70 60 50 40 30 20 10
f1 (ppm)

3ga

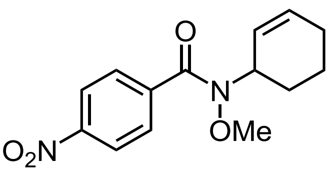

O
N
OMe
O_2N

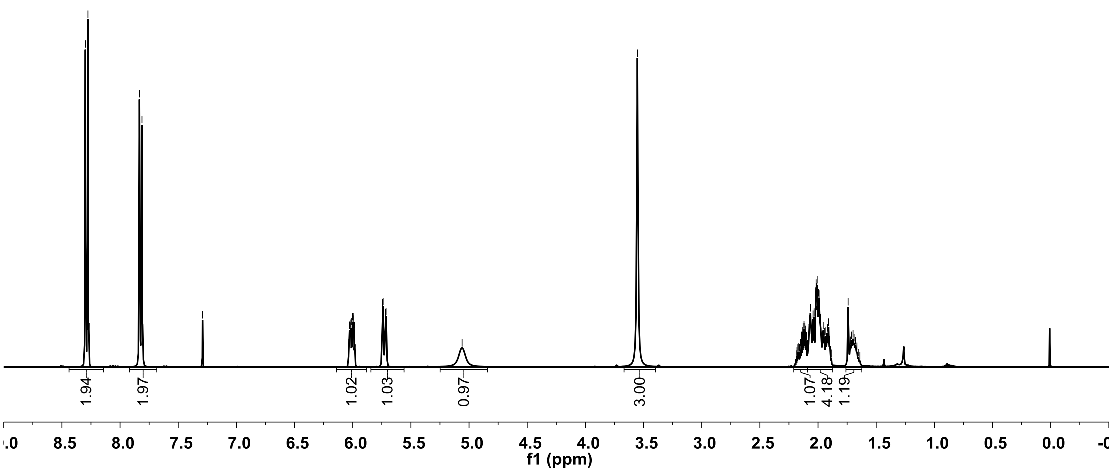

8.304 8.298 8.293 8.281 8.276 8.271 7.839 7.834 7.829 7.816 7.811 7.806 7.291 6.027 6.021 6.016 6.009 6.001 5.996 5.991 5.984 5.744 5.739 5.719 5.713 3.553 2.143 2.136 2.128 2.120 2.113 2.106 2.099 2.074 2.065 2.043 2.038 2.034 2.021 2.016 2.013 2.007 1.996 1.991 1.979 1.971 1.963 1.956 1.943 1.933 1.920 1.910 1.741 1.713 1.699 1.690
1.94
1.97
1.02
1.03
0.97
3.00
1.07
4.18
1.19
8.5 8.0 7.5 7.0 6.5 6.0 5.5 5.0 4.5 4.0 3.5 3.0 2.5 2.0 1.5 1.0 0.5 0.0
f1 (ppm)

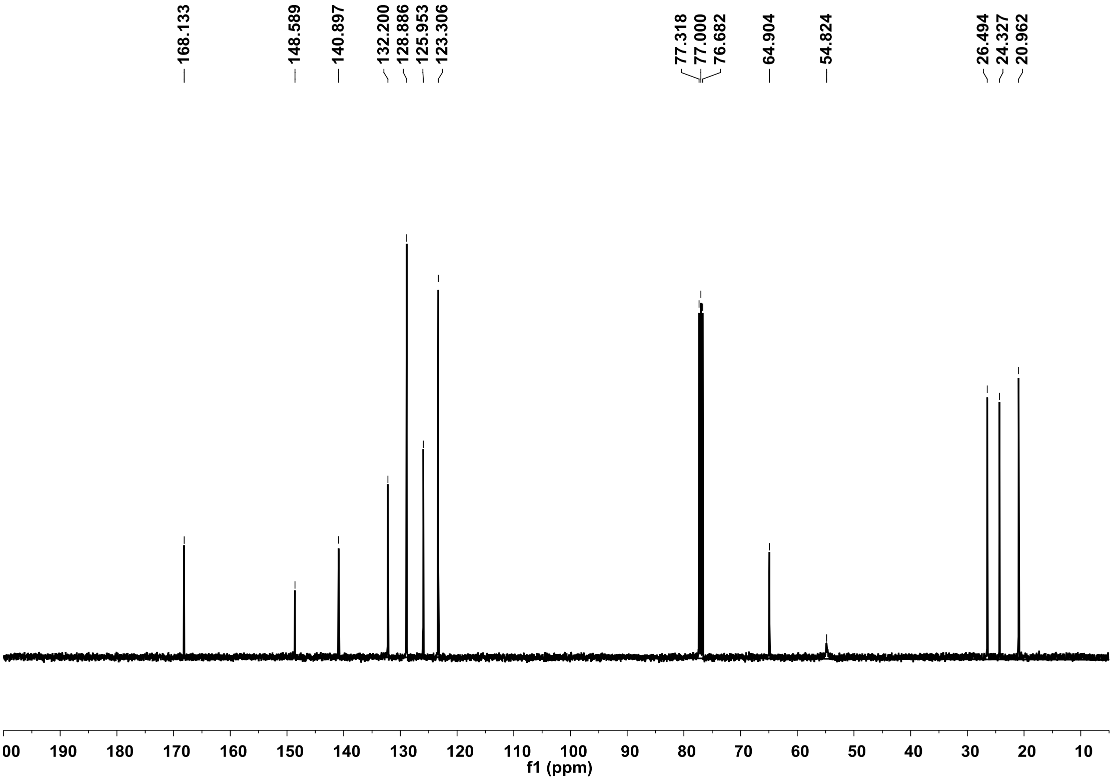

168.133
148.589
140.897
132.200 128.886 125.953 123.306
77.318 77.000 76.682
64.904
54.824
26.494 24.327 20.962
190 180 170 160 150 140 130 120 110 100 90 80 70 60 50 40 30 20 10
f1 (ppm)

3ha

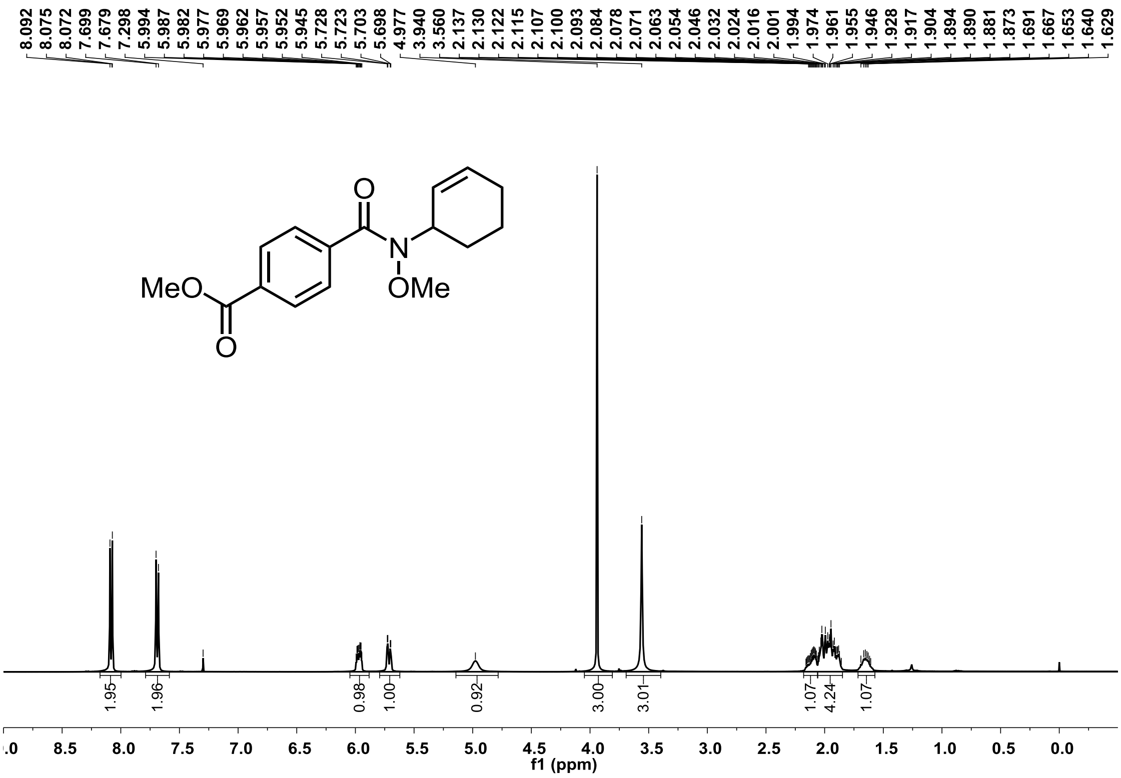
8.092
8.075
8.072
7.699
7.679
7.298
5.994
5.987
5.982
5.977
5.969
5.962
5.957
5.952
5.945
5.728
5.723
5.703
5.698
4.977
3.940
3.560
2.137
2.130
2.122
2.115
2.107
2.100
2.093
2.084
2.078
2.071
2.063
2.054
2.046
2.032
2.024
2.016
2.001
1.994
1.974
1.961
1.955
1.946
1.928
1.917
1.904
1.894
1.890
1.881
1.873
1.691
1.667
1.653
1.640
1.629
MeO
O
O
N
OMe
1.95
1.96
0.98
1.00
0.92
3.00
3.01
1.07
4.24
1.07
8.5
8.0
7.5
7.0
6.5
6.0
5.5
5.0
4.5
4.0
3.5
3.0
2.5
2.0
1.5
1.0
0.5
0.0
f1 (ppm)

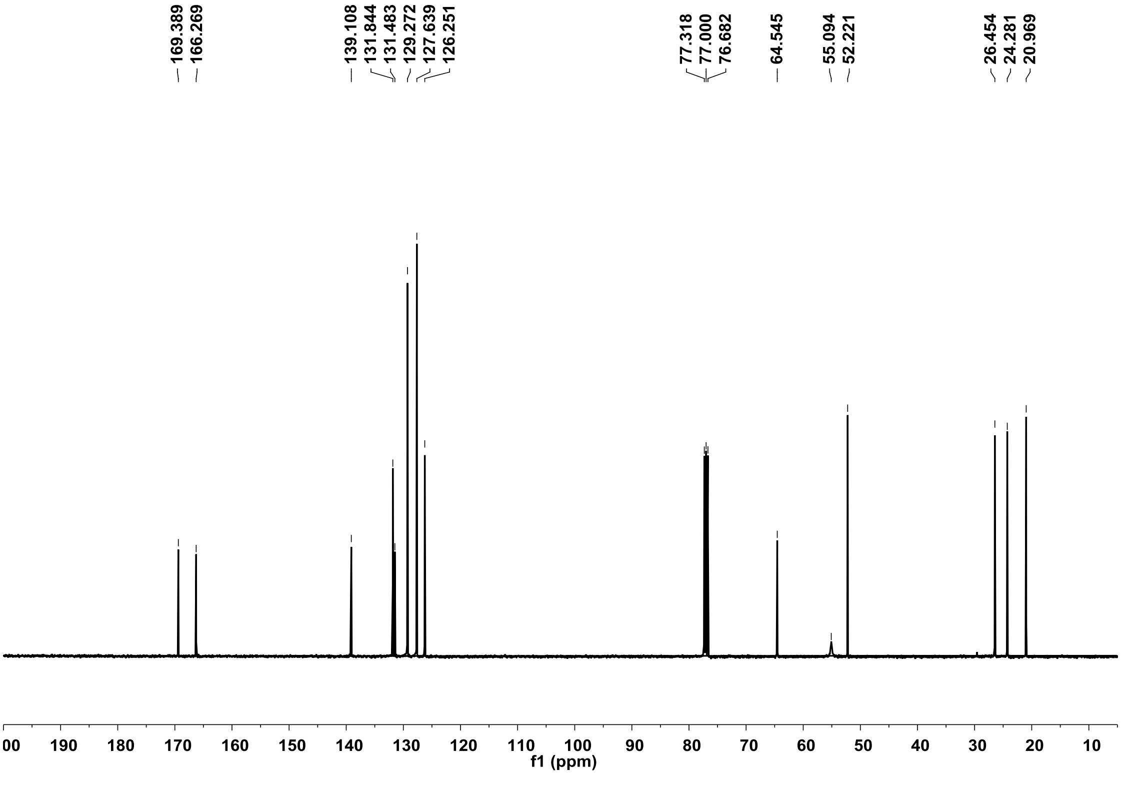
169.389
166.269
139.108
131.844
131.483
129.272
127.639
126.251
77.318
77.000
76.682
64.545
55.094
52.221
26.454
24.281
20.969
190
180
170
160
150
140
130
120
110
100
90
80
70
60
50
40
30
20
10
f1 (ppm)

3ia

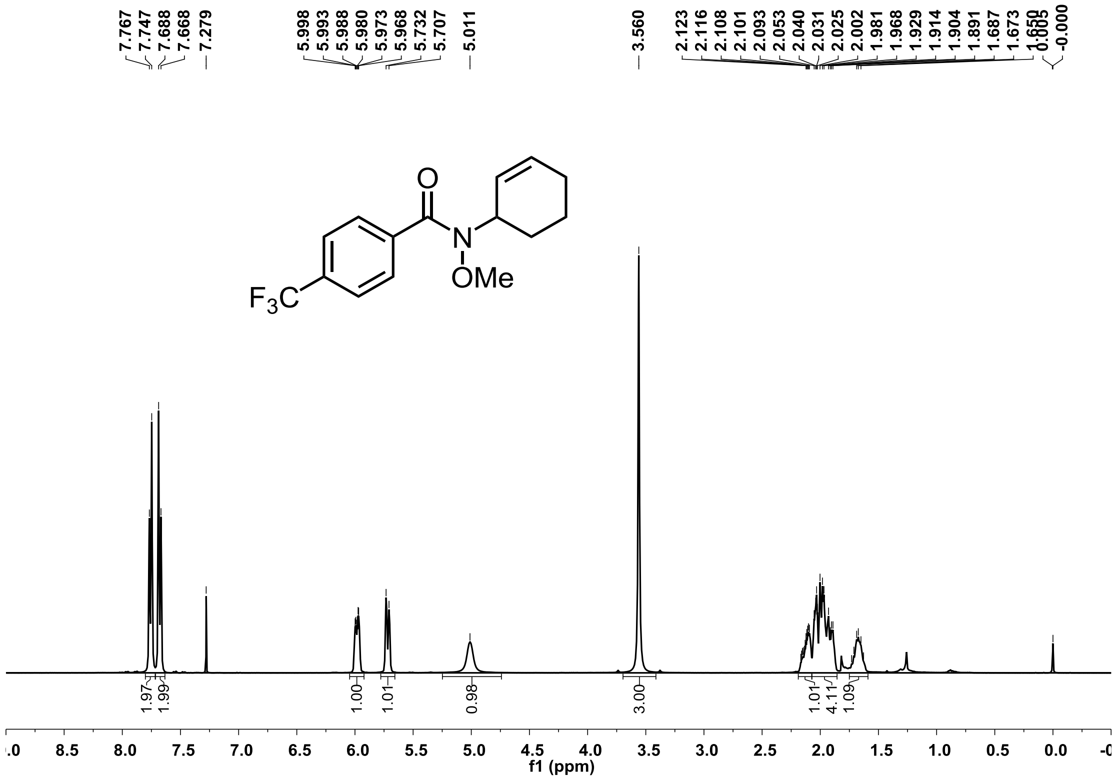

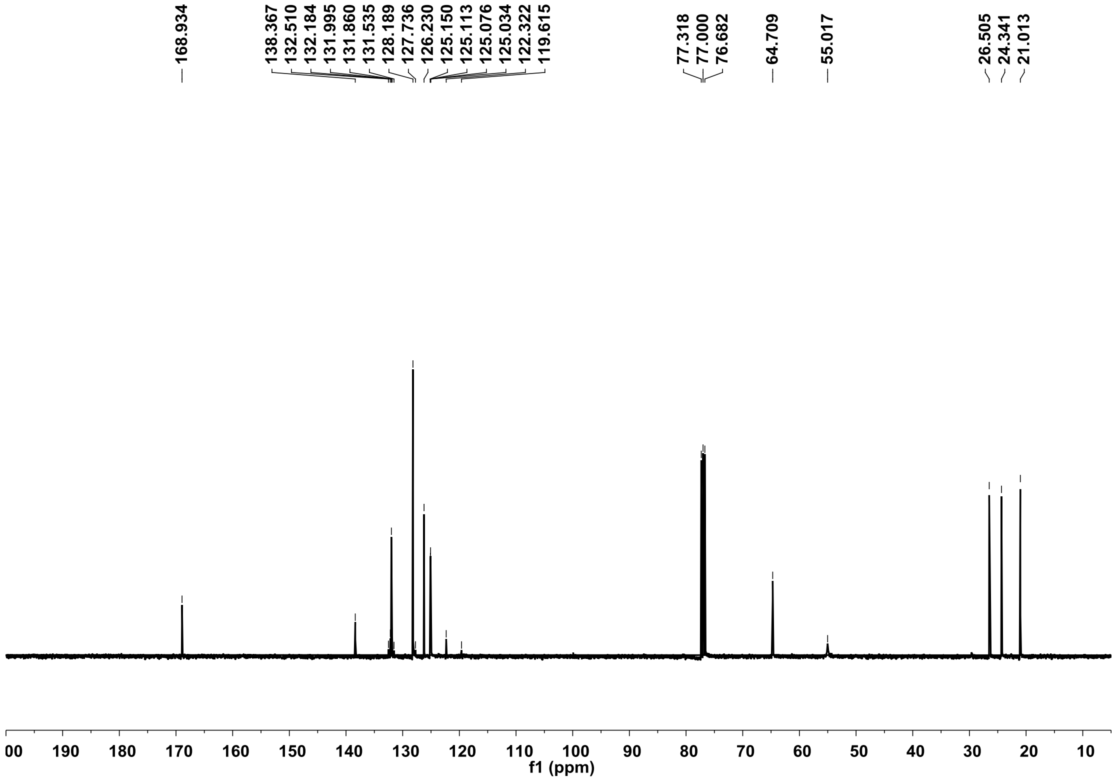

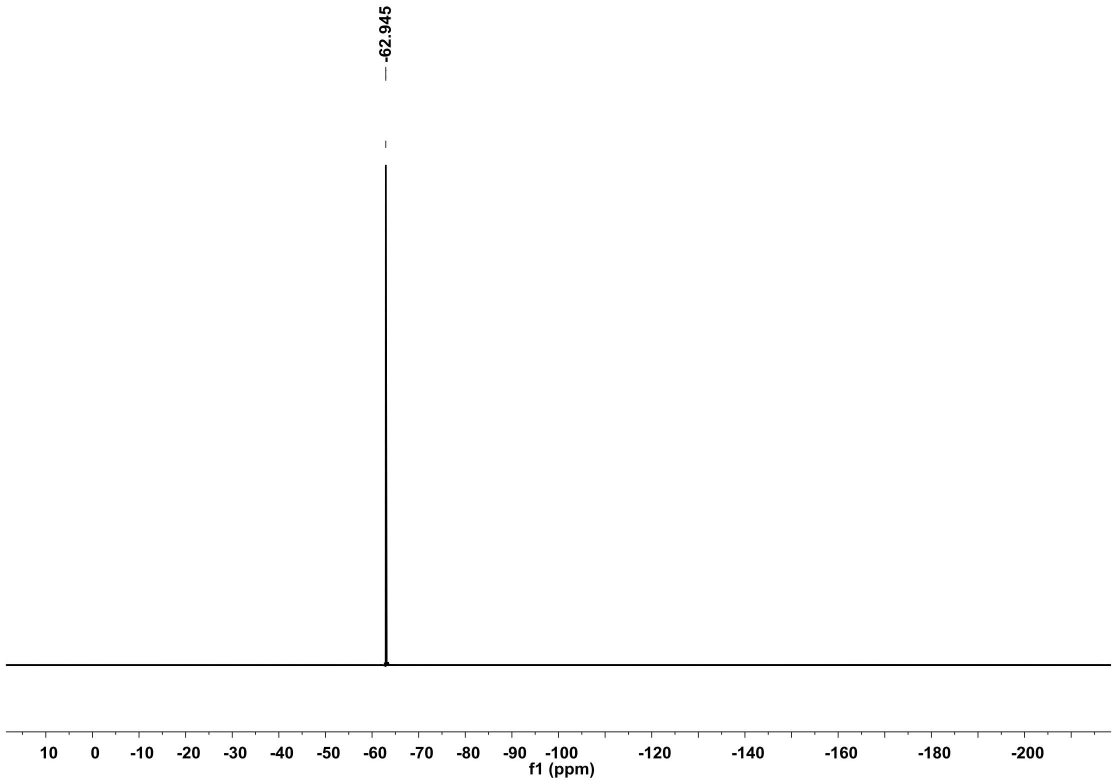
-62.945
10 0 -10 -20 -30 -40 -50 -60 -70 -80 -90 -100 -120 -140 -160 -180 -200
f1 (ppm)

3ja

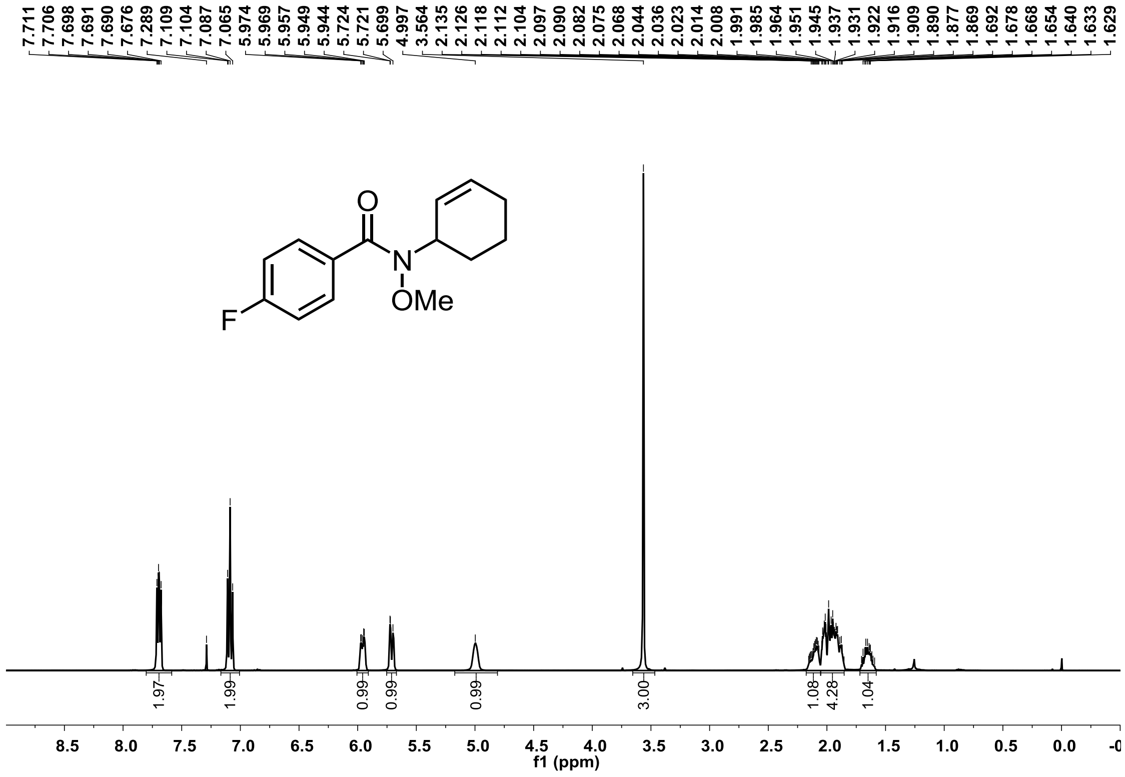
7.711
7.706
7.698
7.691
7.690
7.676
7.289
7.109
7.104
7.087
7.065
5.974
5.969
5.957
5.949
5.944
5.724
5.721
5.699
4.997
3.564
2.135
2.126
2.118
2.112
2.104
2.097
2.090
2.082
2.075
2.068
2.044
2.036
2.023
2.014
2.008
1.991
1.985
1.964
1.951
1.945
1.937
1.931
1.922
1.916
1.909
1.890
1.877
1.869
1.692
1.678
1.668
1.654
1.640
1.633
1.629
O
N
OMe
F
1.97
1.99
0.99
0.99
0.99
3.00
1.08
4.28
1.04
f1 (ppm)

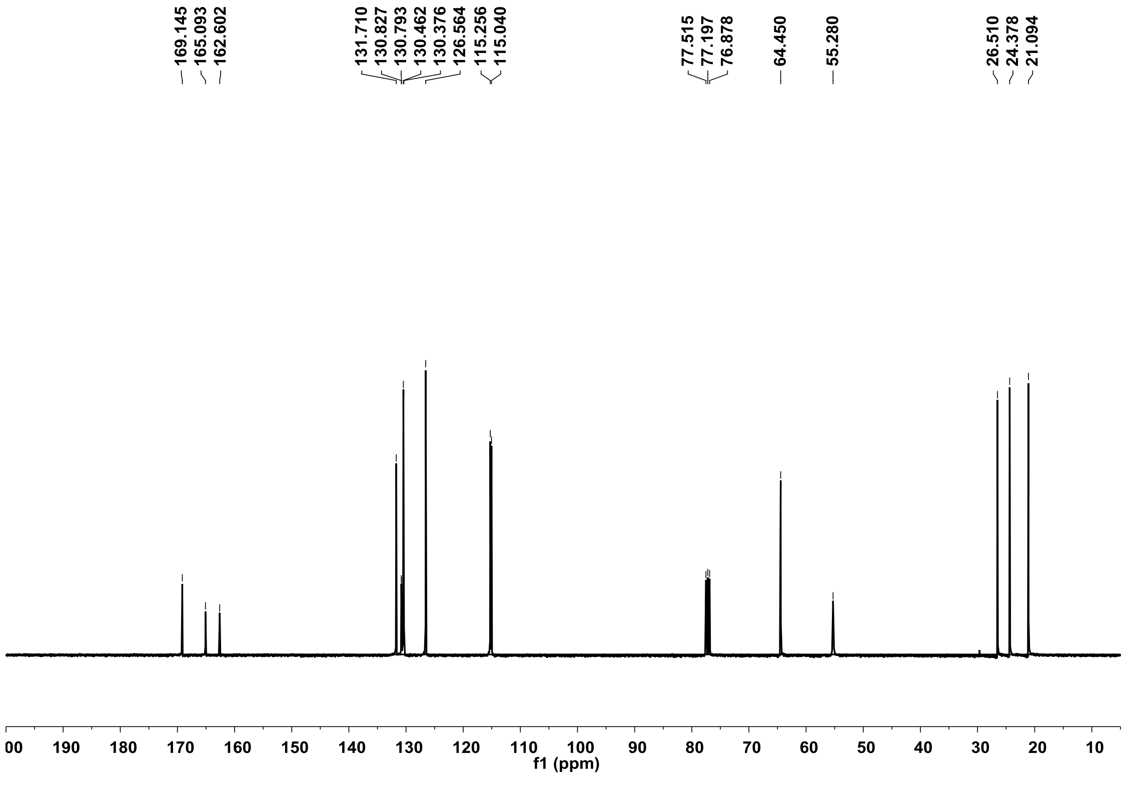
169.145
165.093
162.602
131.710
130.827
130.793
130.462
130.376
126.564
115.256
115.040
77.515
77.197
76.878
64.450
55.280
26.510
24.378
21.094
f1 (ppm)

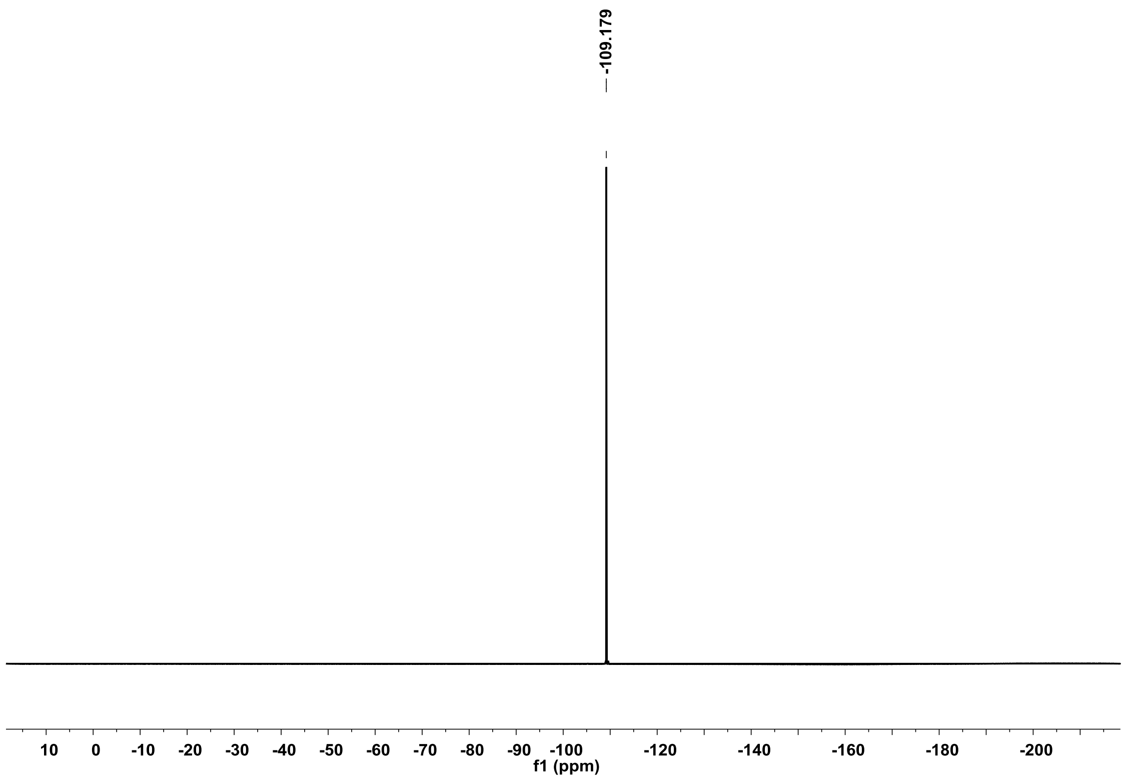
-109.179
10
0
-10
-20
-30
-40
-50
-60
-70
-80
-90
-100
-120
-140
-160
-180
-200
f1 (ppm)

3ka

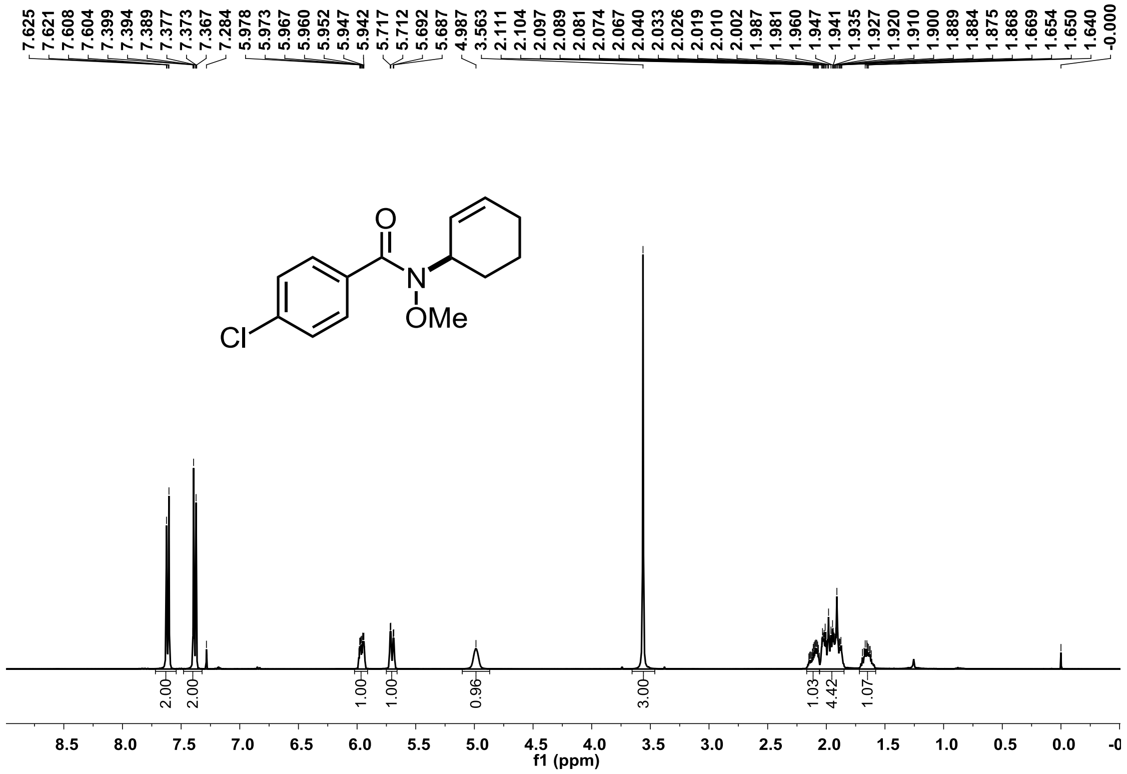
7.625 7.621 7.608 7.604 7.399 7.394 7.389 7.377 7.373 7.367 7.284 5.978 5.973 5.967 5.960 5.952 5.947 5.942 5.717 5.712 5.692 5.687 4.987 3.563 2.111 2.104 2.097 2.089 2.081 2.074 2.067 2.040 2.033 2.026 2.019 2.010 2.002 1.987 1.981 1.960 1.947 1.941 1.935 1.927 1.920 1.910 1.900 1.889 1.884 1.875 1.868 1.669 1.654 1.650 1.640 -0.000
O
N
OMe
Cl
2.00 2.00 1.00 1.00 0.96 3.00 1.03 4.42 1.07
f1 (ppm)

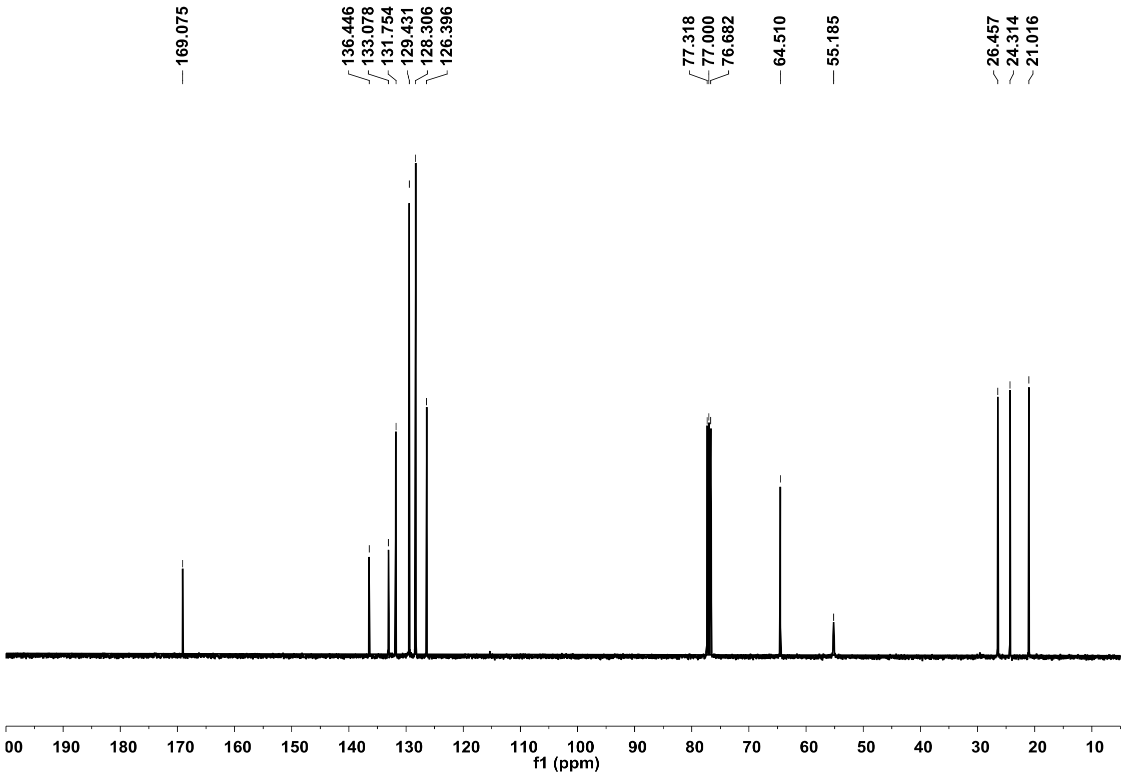
169.075
136.446 133.078 131.754 129.431 128.306 126.396
77.318 77.000 76.682
64.510
55.185
26.457 24.314 21.016
f1 (ppm)

3la

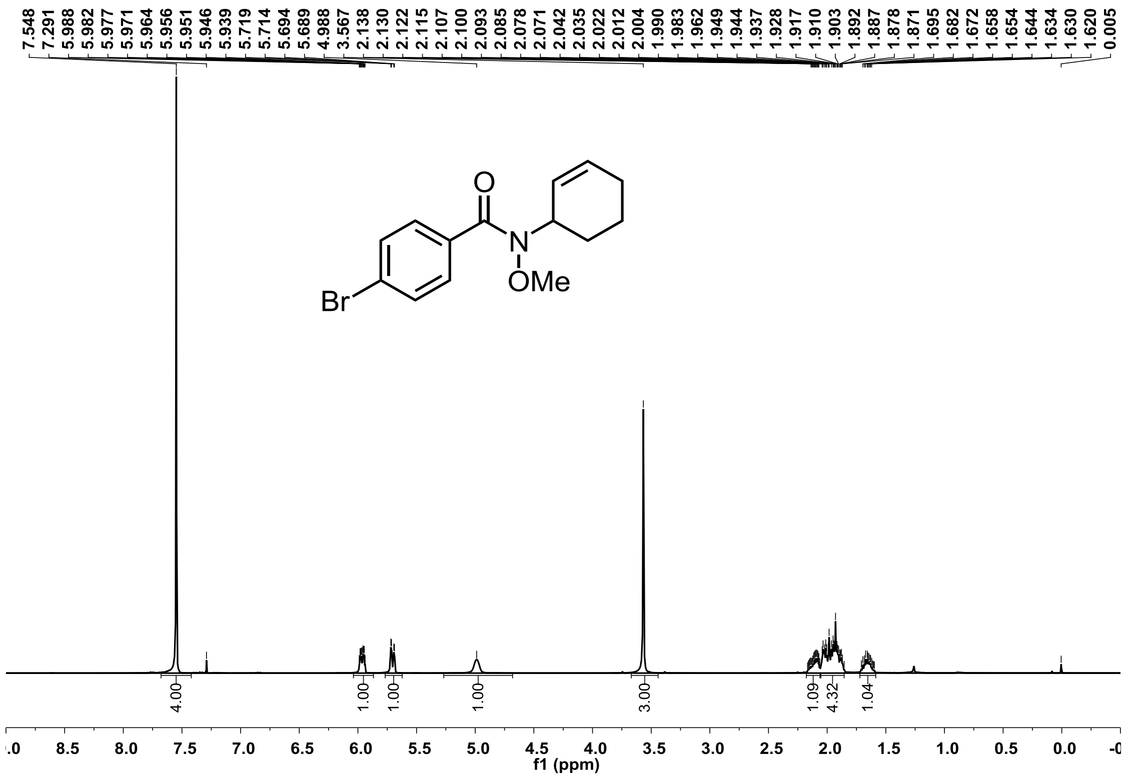

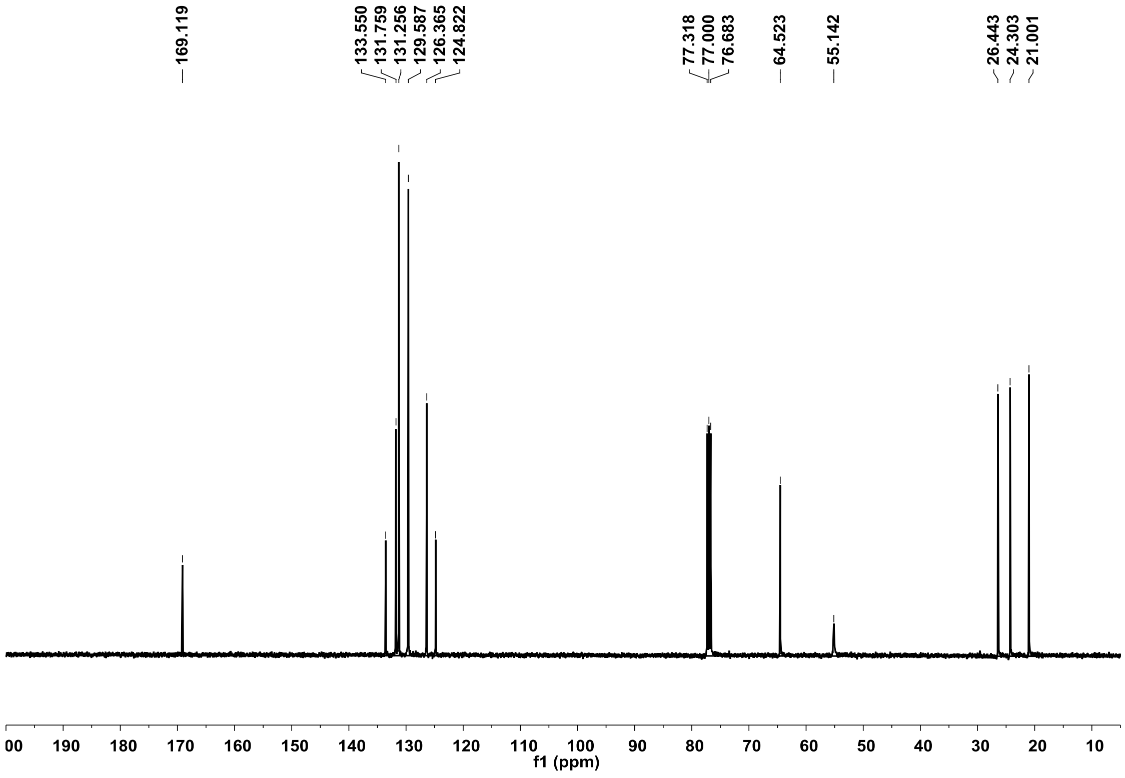

3ma

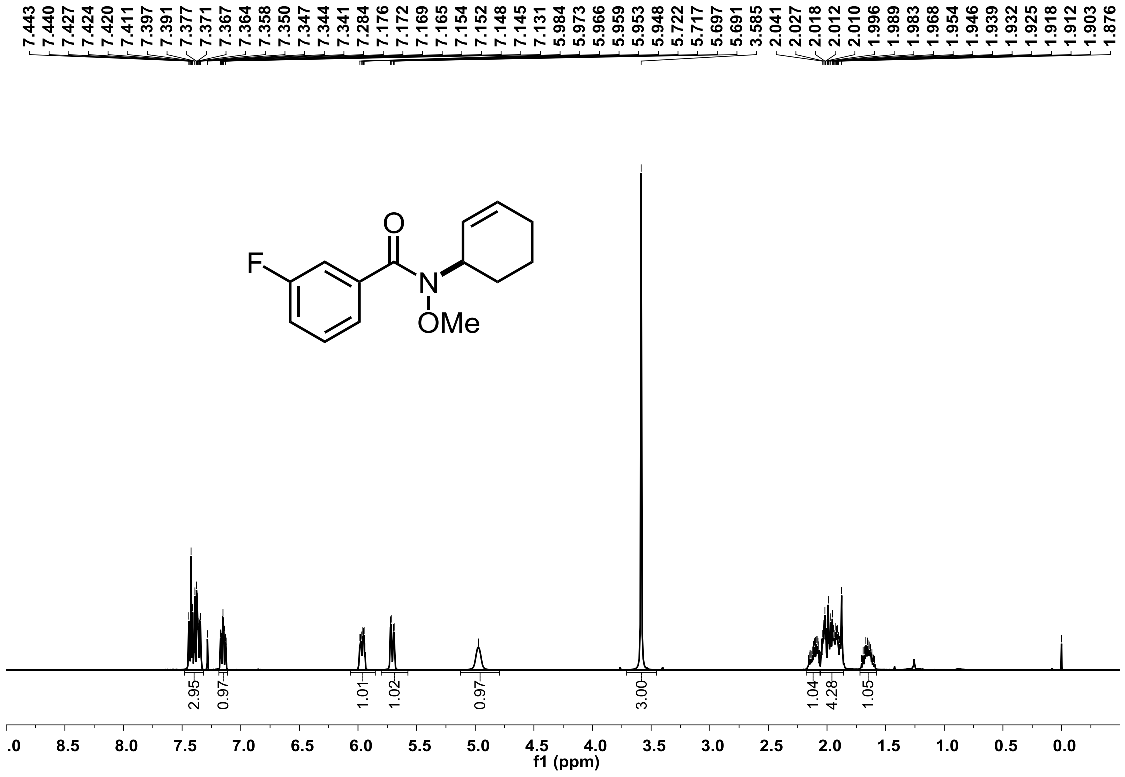

7.443
7.440
7.427
7.424
7.420
7.411
7.397
7.391
7.377
7.371
7.367
7.364
7.358
7.350
7.347
7.344
7.341
7.284
7.176
7.172
7.169
7.165
7.154
7.152
7.148
7.145
7.131
5.984
5.973
5.966
5.959
5.953
5.948
5.722
5.717
5.697
5.691
3.585
2.041
2.027
2.018
2.012
2.010
1.996
1.989
1.983
1.968
1.954
1.946
1.939
1.932
1.925
1.918
1.912
1.903
1.876
F
O
N
OMe
2.95
0.97
1.01
1.02
0.97
3.00
1.04
4.28
1.05
f1 (ppm)

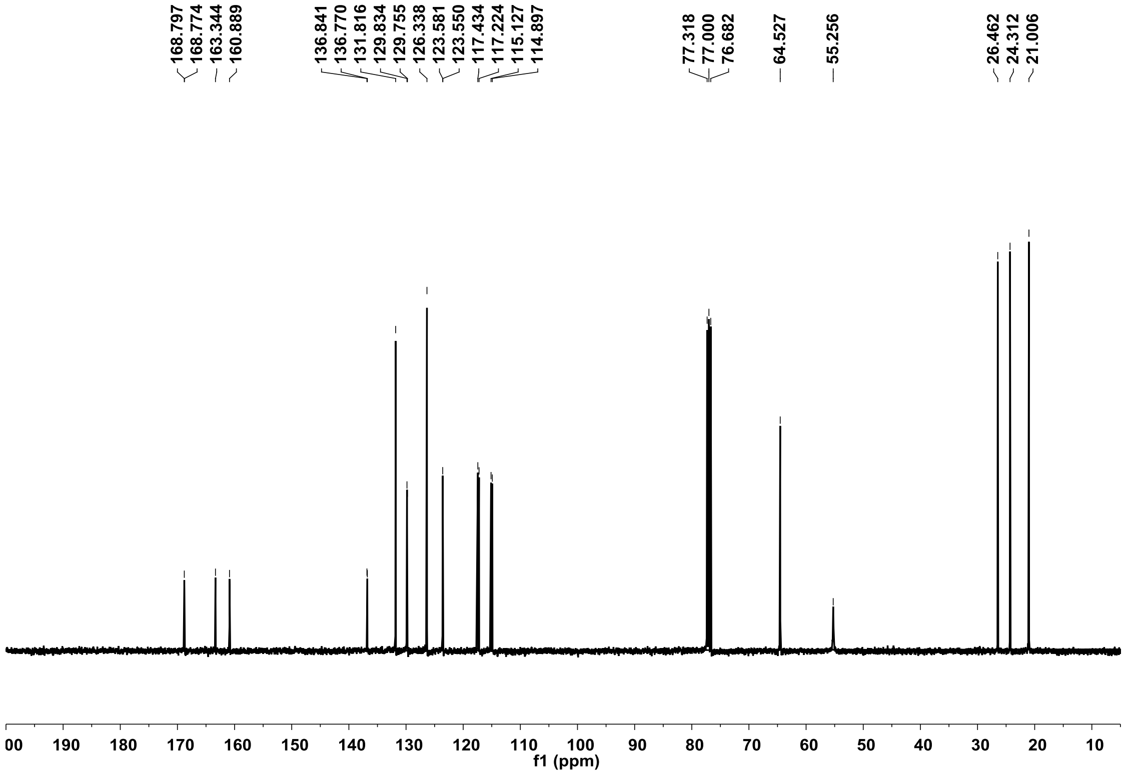

168.797
168.774
163.344
160.889
136.841
136.770
131.816
129.834
129.755
126.338
123.581
123.550
117.434
117.224
115.127
114.897
77.318
77.000
76.682
64.527
55.256
26.462
24.312
21.006
f1 (ppm)

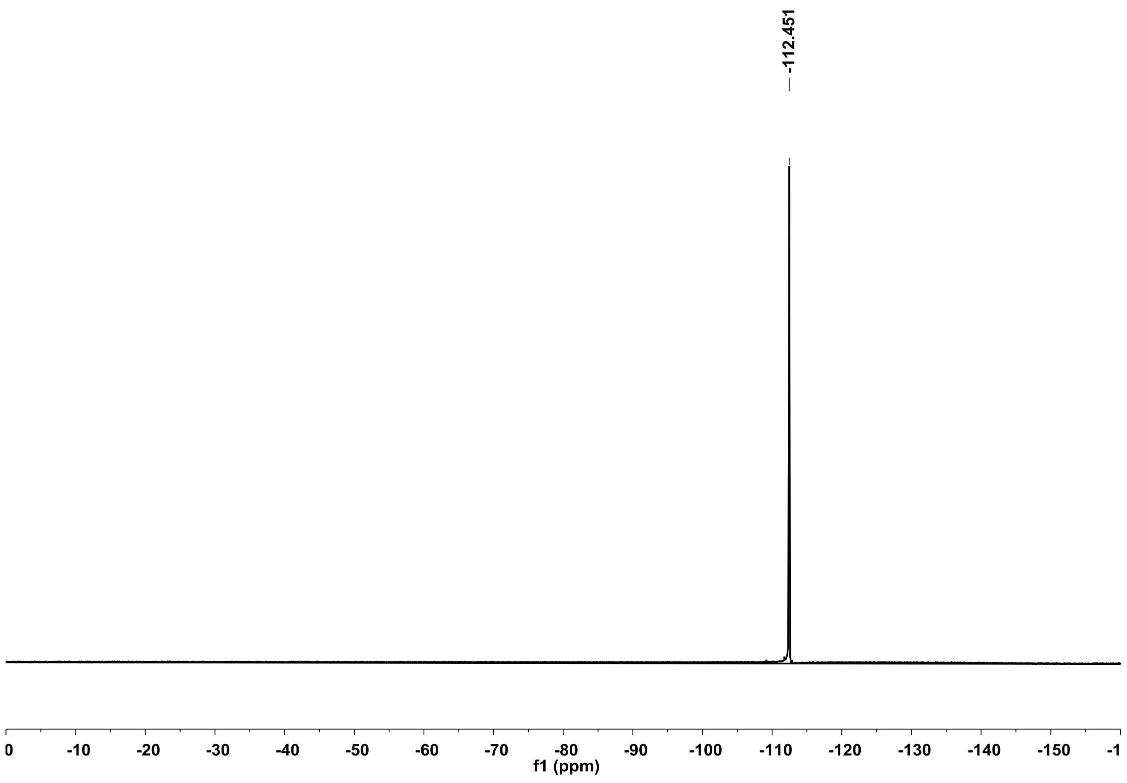
-112.451
0
-10
-20
-30
-40
-50
-60
-70
-80
-90
-100
-110
-120
-130
-140
-150
-1
f1 (ppm)

3na

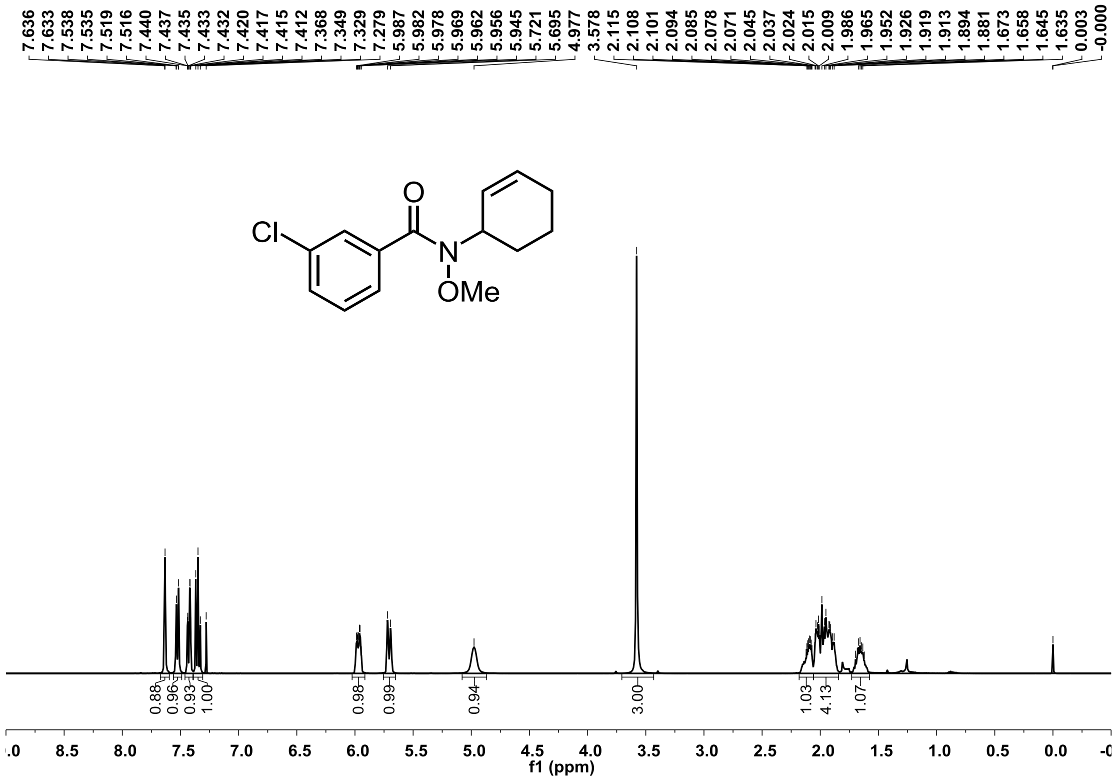
7.636
7.633
7.538
7.535
7.519
7.516
7.440
7.437
7.435
7.433
7.432
7.420
7.417
7.415
7.412
7.368
7.349
7.329
7.279
5.987
5.982
5.978
5.969
5.962
5.956
5.945
5.721
5.695
4.977
3.578
2.115
2.108
2.101
2.094
2.085
2.078
2.071
2.045
2.037
2.024
2.015
2.009
1.986
1.965
1.952
1.926
1.919
1.913
1.894
1.881
1.673
1.658
1.645
1.635
0.003
-0.000
Cl
O
N
OMe
0.88
0.96
0.93
1.00
0.98
0.99
0.94
3.00
1.03
4.13
1.07
f1 (ppm)

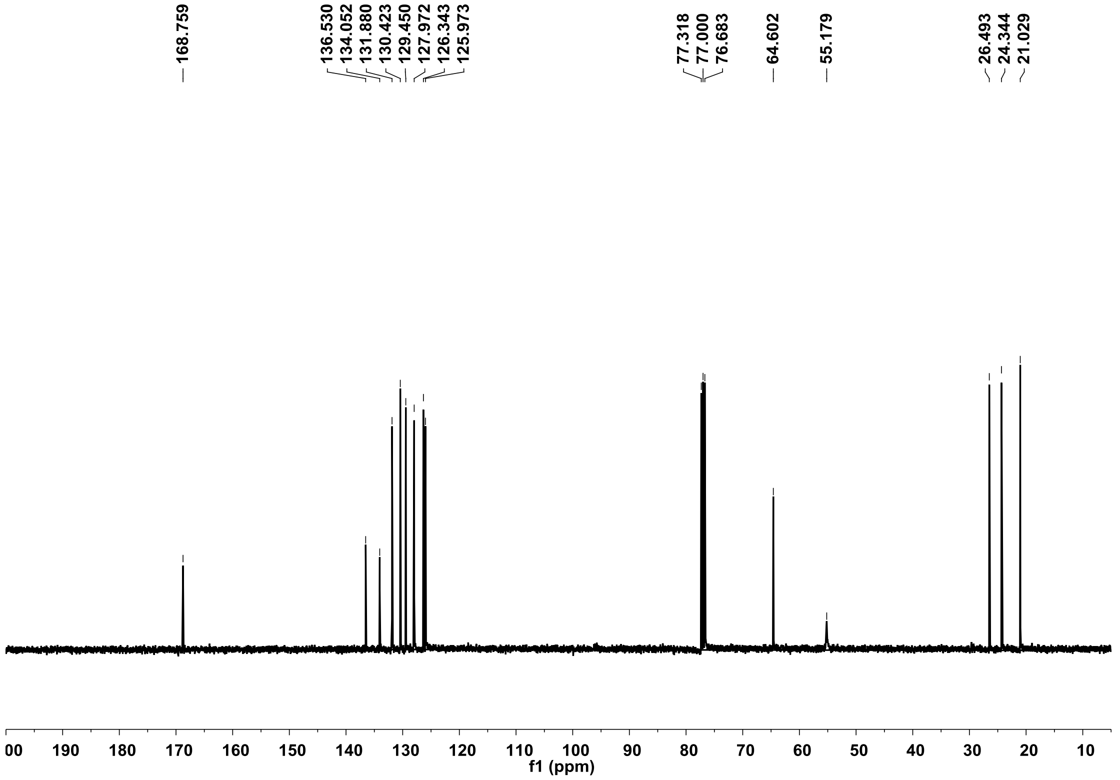
168.759
136.530
134.052
131.880
130.423
129.450
127.972
126.343
125.973
77.318
77.000
76.683
64.602
55.179
26.493
24.344
21.029
f1 (ppm)

3oa

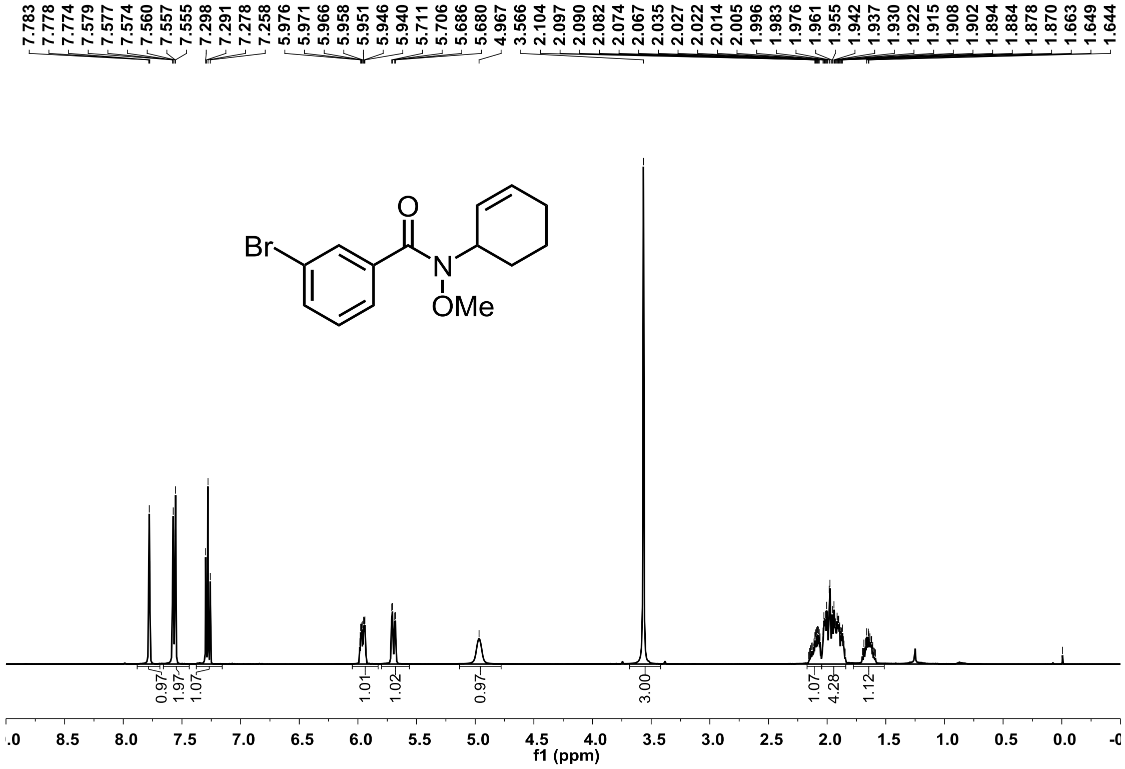
7.783
7.778
7.774
7.579
7.577
7.574
7.560
7.557
7.555
7.298
7.291
7.278
7.258
5.976
5.971
5.966
5.958
5.951
5.946
5.940
5.711
5.706
5.686
5.680
4.967
3.566
2.104
2.097
2.090
2.082
2.074
2.067
2.035
2.027
2.022
2.014
2.005
1.996
1.983
1.976
1.961
1.955
1.942
1.937
1.930
1.922
1.915
1.908
1.902
1.894
1.884
1.878
1.870
1.663
1.649
1.644
0.97
1.97
1.07
1.01
1.02
0.97
3.00
1.07
4.28
1.12
f1 (ppm)

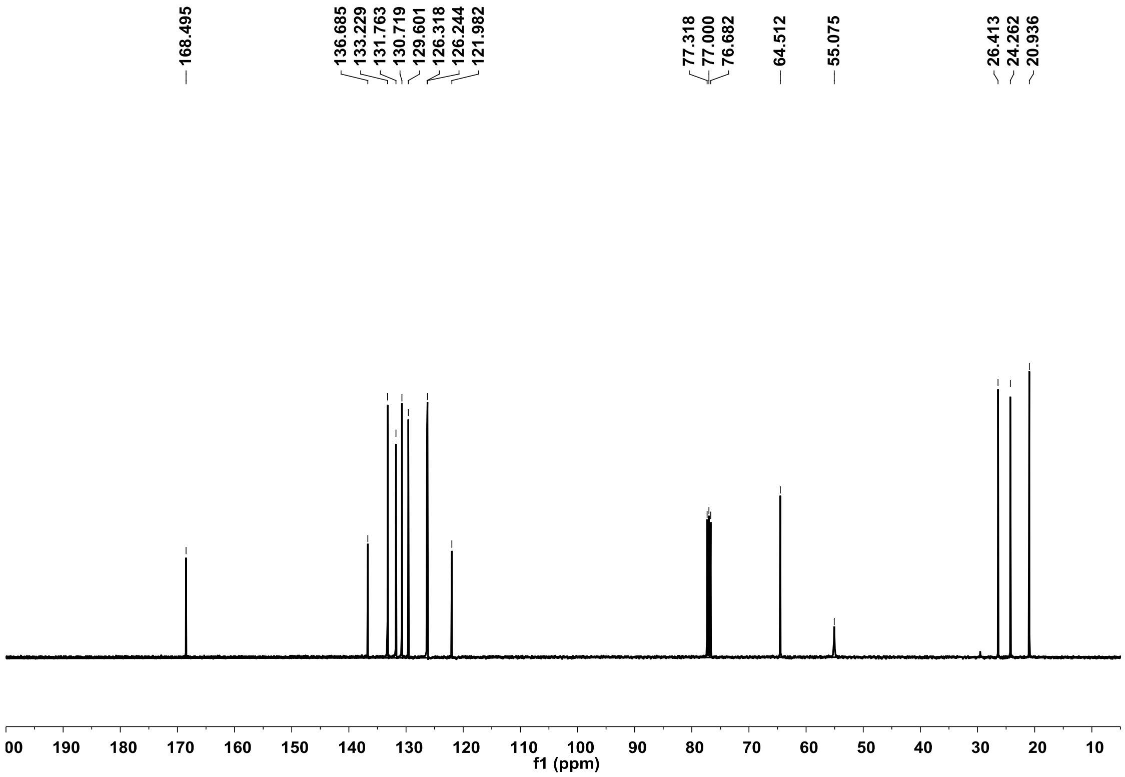
168.495
136.685
133.229
131.763
130.719
129.601
126.318
126.244
121.982
77.318
77.000
76.682
64.512
55.075
26.413
24.262
20.936
f1 (ppm)

3pa

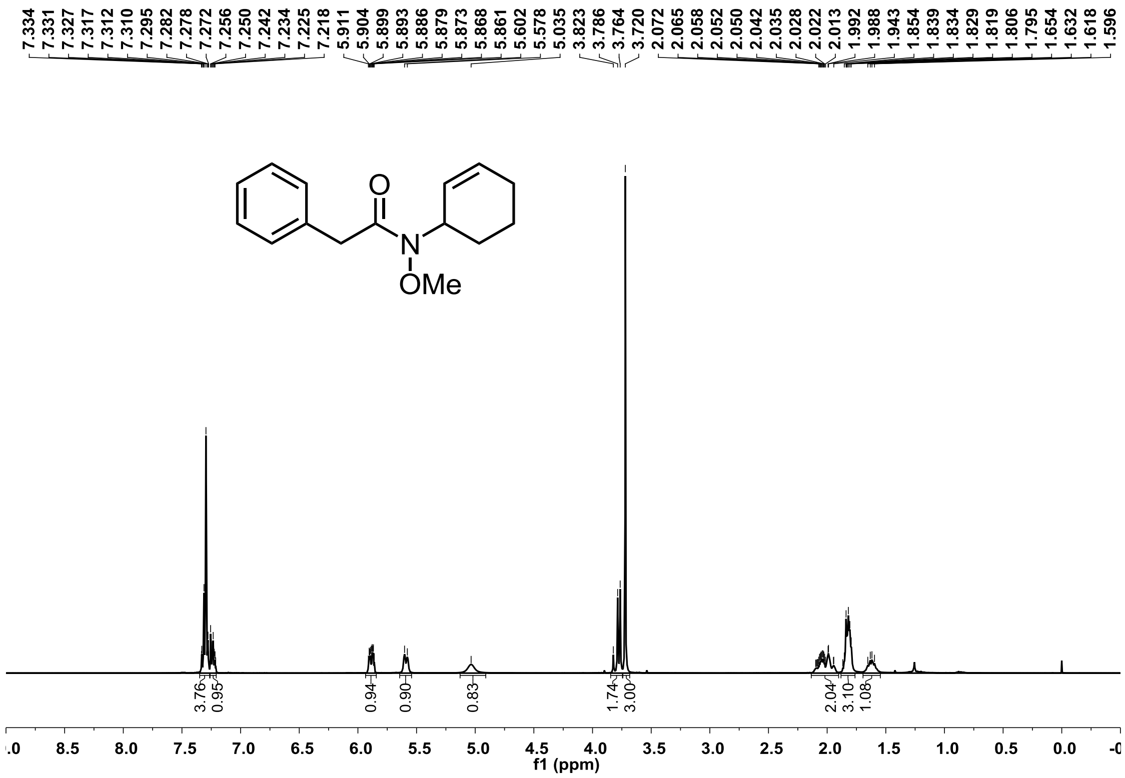
7.334
7.331
7.327
7.317
7.312
7.310
7.295
7.282
7.278
7.272
7.256
7.250
7.242
7.234
7.225
7.218
5.911
5.904
5.899
5.893
5.886
5.879
5.873
5.868
5.861
5.602
5.578
5.035
3.823
3.786
3.764
3.720
2.072
2.065
2.058
2.052
2.050
2.042
2.035
2.028
2.022
2.013
1.992
1.988
1.943
1.854
1.839
1.834
1.829
1.819
1.806
1.795
1.654
1.632
1.618
1.596
O
N
OMe
3.76
0.95
0.94
0.90
0.83
1.74
3.00
2.04
3.10
1.08
8.5
8.0
7.5
7.0
6.5
6.0
5.5
5.0
4.5
4.0
3.5
3.0
2.5
2.0
1.5
1.0
0.5
0.0
f1 (ppm)

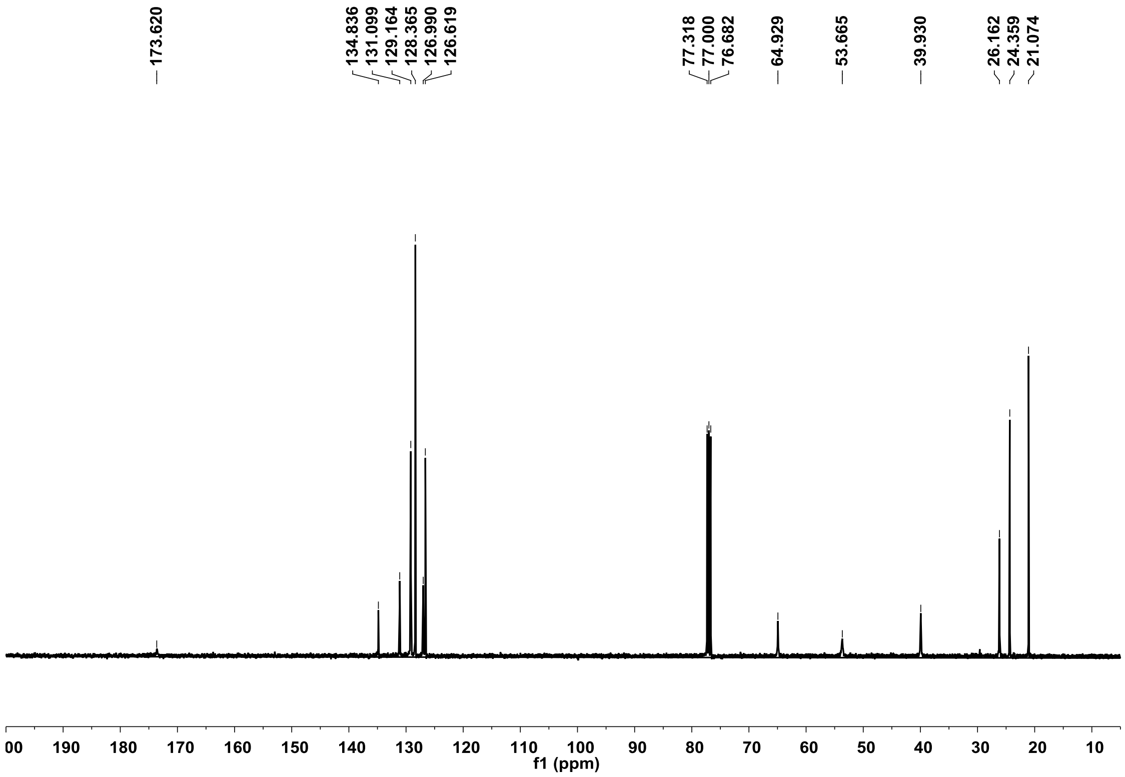
173.620
134.836
131.099
129.164
128.365
126.990
126.619
77.318
77.000
76.682
64.929
53.665
39.930
26.162
24.359
21.074
190
180
170
160
150
140
130
120
110
100
90
80
70
60
50
40
30
20
10
f1 (ppm)

3qa

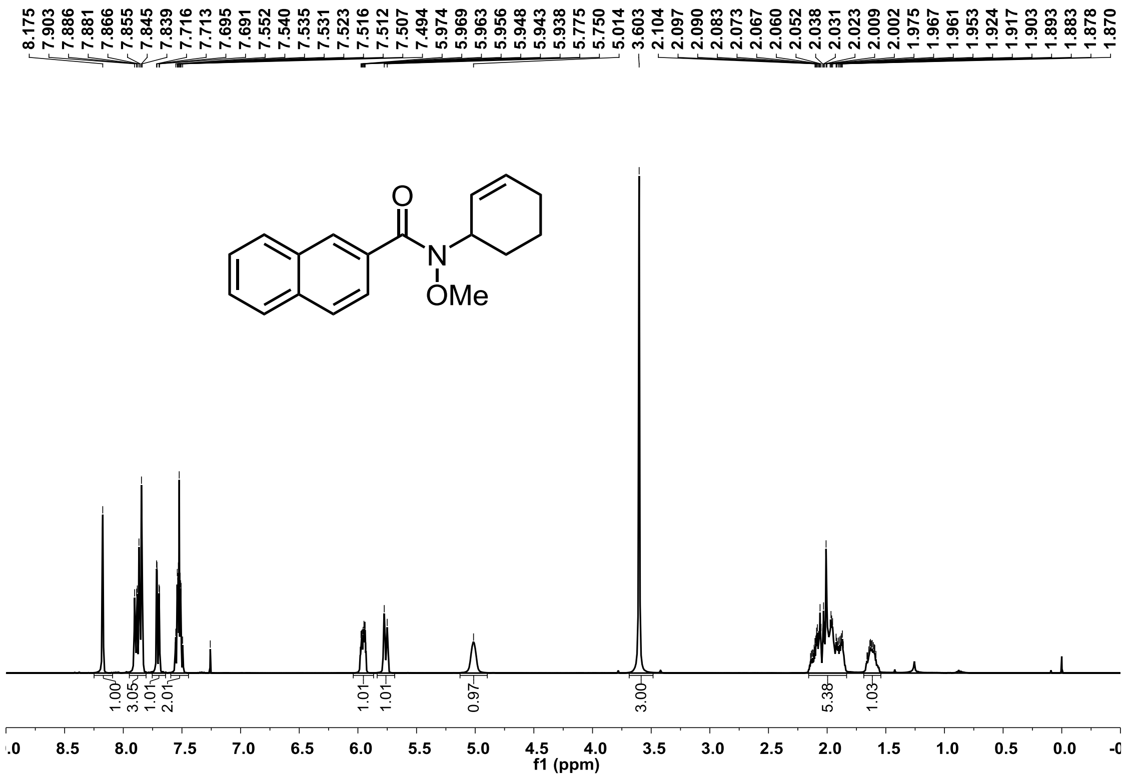
8.175 7.903 7.886 7.881 7.866 7.855 7.845 7.839 7.716 7.713 7.695 7.691 7.552 7.540 7.535 7.531 7.523 7.516 7.512 7.507 7.494 5.974 5.969 5.963 5.956 5.948 5.943 5.938 5.775 5.750 5.014
3.603
2.104 2.097 2.090 2.083 2.073 2.067 2.060 2.052 2.038 2.031 2.023 2.009 2.002 1.975 1.967 1.961 1.953 1.924 1.917 1.903 1.893 1.883 1.878 1.870
O
N
OMe
1.00 3.05 1.01 2.01 1.01 1.01 0.97 3.00 5.38 1.03
f1 (ppm)

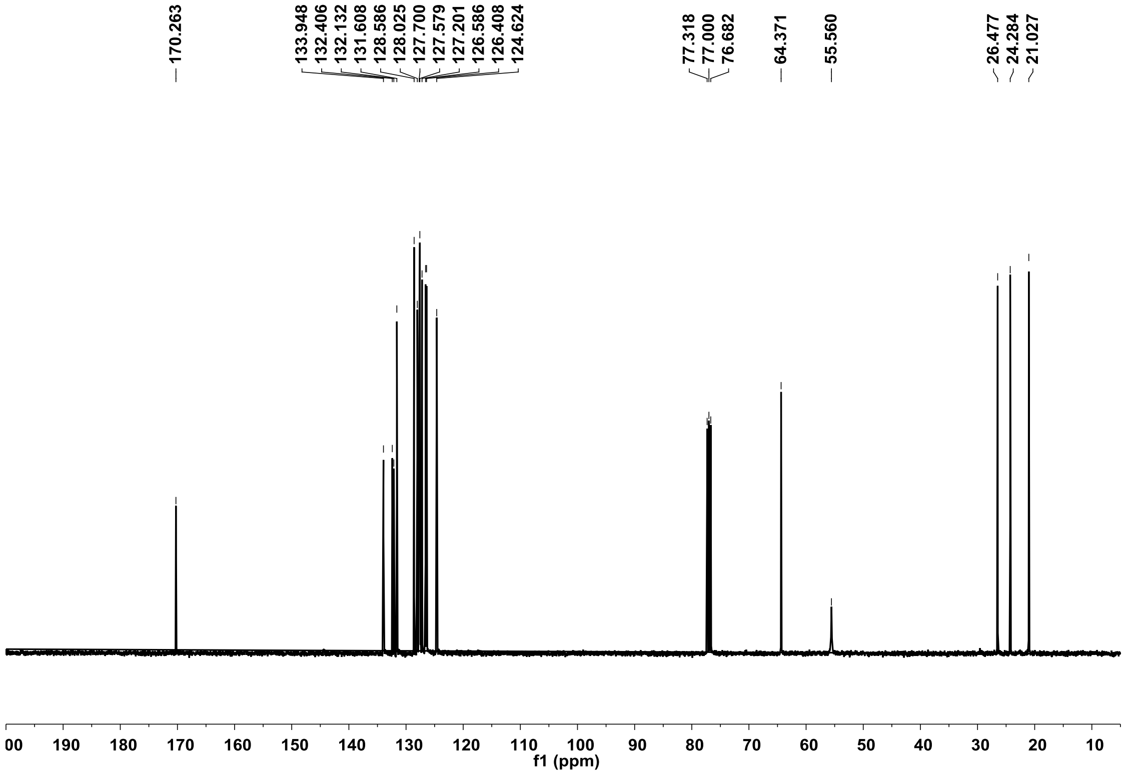
170.263
133.948 132.406 132.132 131.608 128.586 128.025 127.700 127.579 127.201 126.586 126.408 124.624
77.318 77.000 76.682
64.371
55.560
26.477 24.284 21.027
f1 (ppm)

3ra

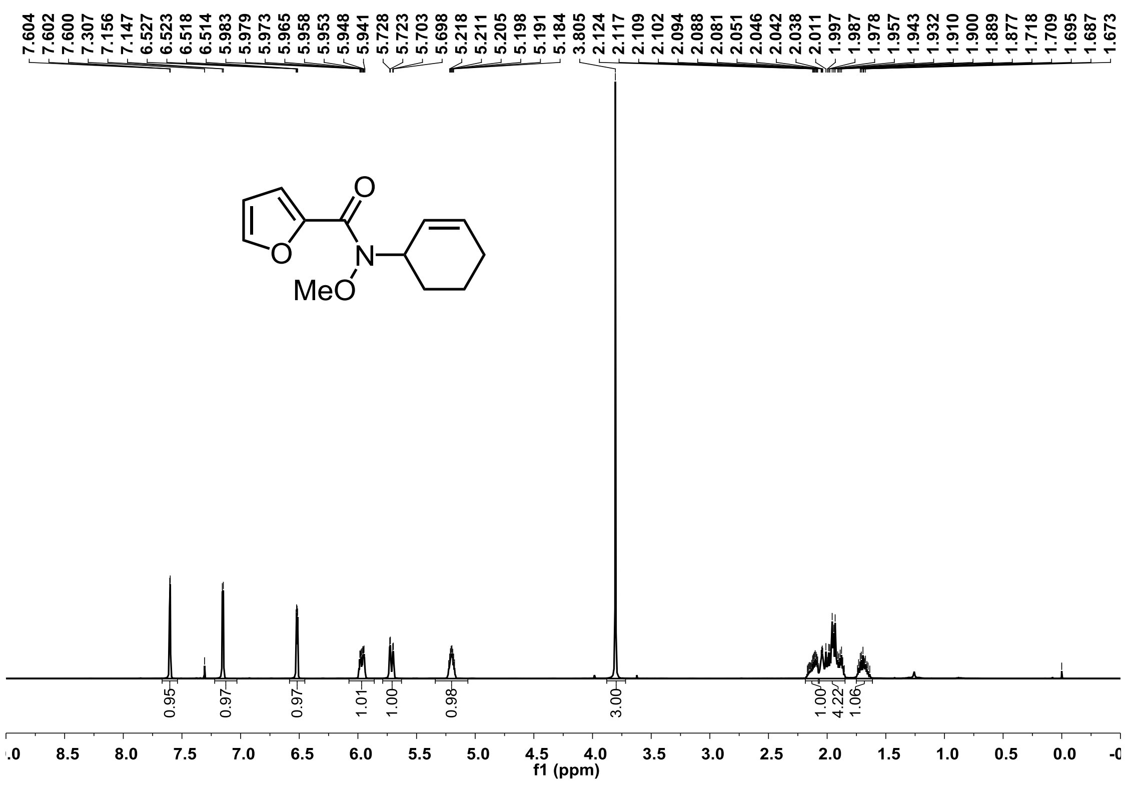
7.604 7.602 7.600 7.307 7.156 7.147 6.527 6.523 6.518 6.514 5.983 5.979 5.973 5.965 5.958 5.953 5.948 5.941 5.728 5.723 5.703 5.698 5.218 5.211 5.205 5.198 5.191 5.184 3.805 2.124 2.117 2.109 2.102 2.094 2.088 2.081 2.051 2.046 2.042 2.038 2.011 1.997 1.987 1.978 1.957 1.943 1.932 1.910 1.900 1.889 1.877 1.718 1.709 1.695 1.687 1.673
O
N
MeO
0.95 0.97 0.97 1.01 1.00 0.98 3.00 1.00 4.22 1.06
f1 (ppm)

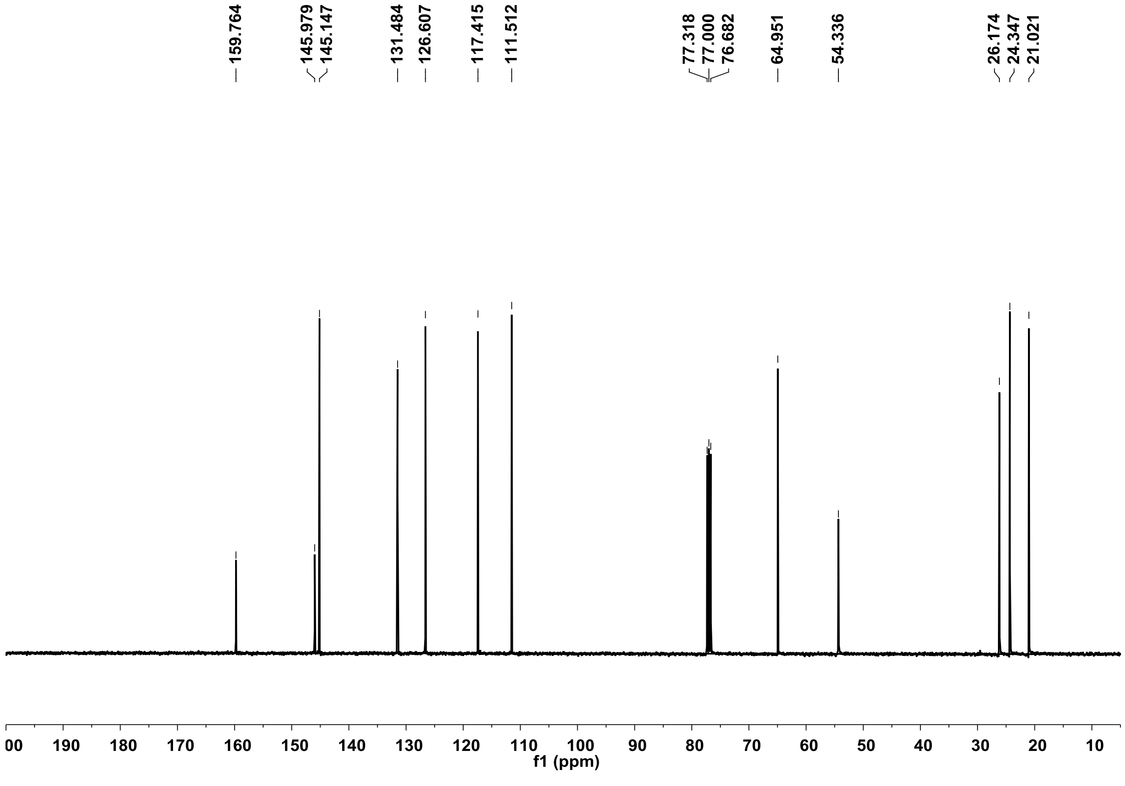
159.764 145.979 145.147 131.484 126.607 117.415 111.512 77.318 77.000 76.682 64.951 54.336 26.174 24.347 21.021
f1 (ppm)

3sa

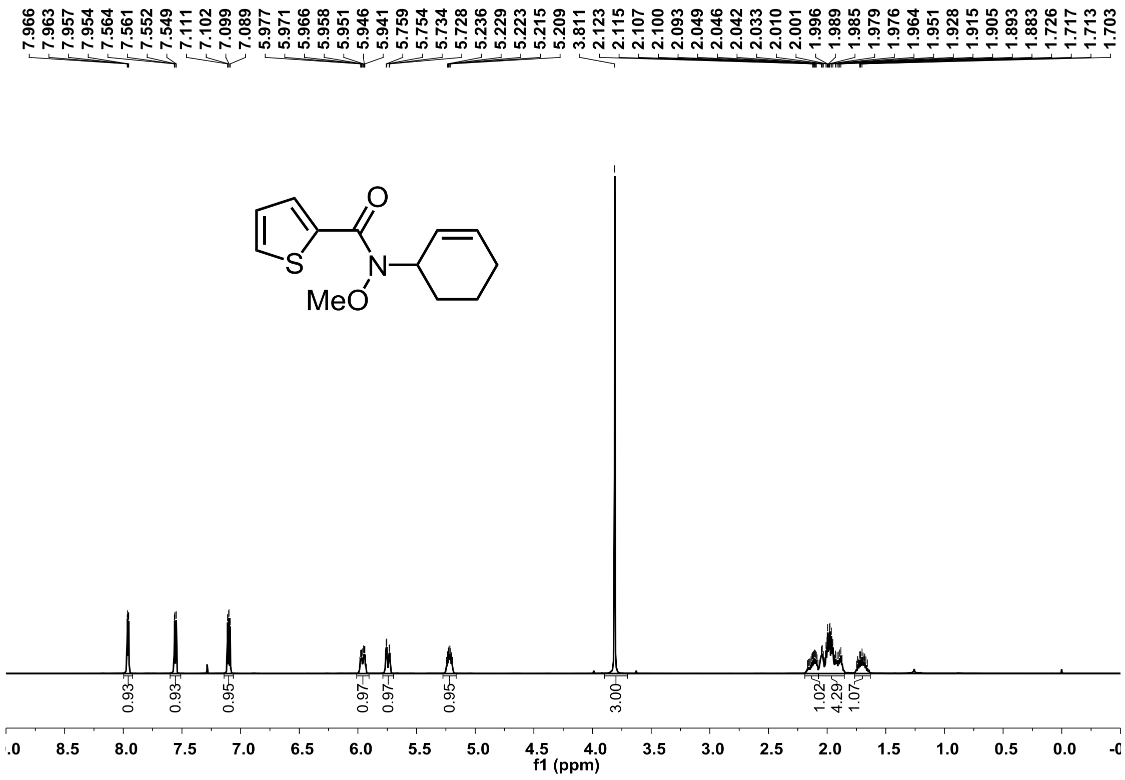
7.966 7.963 7.957 7.954 7.564 7.561 7.552 7.549 7.111 7.102 7.099 7.089 5.977 5.971 5.966 5.958 5.951 5.946 5.941 5.759 5.754 5.734 5.728 5.236 5.229 5.223 5.215 5.209 3.811 2.123 2.115 2.107 2.100 2.093 2.049 2.046 2.042 2.033 2.010 2.001 1.996 1.989 1.985 1.979 1.976 1.964 1.951 1.928 1.915 1.905 1.893 1.883 1.726 1.717 1.713 1.703
O
N
S
MeO
0.93
0.93
0.95
0.97
0.97
0.95
3.00
1.02
4.29
1.07
f1 (ppm)

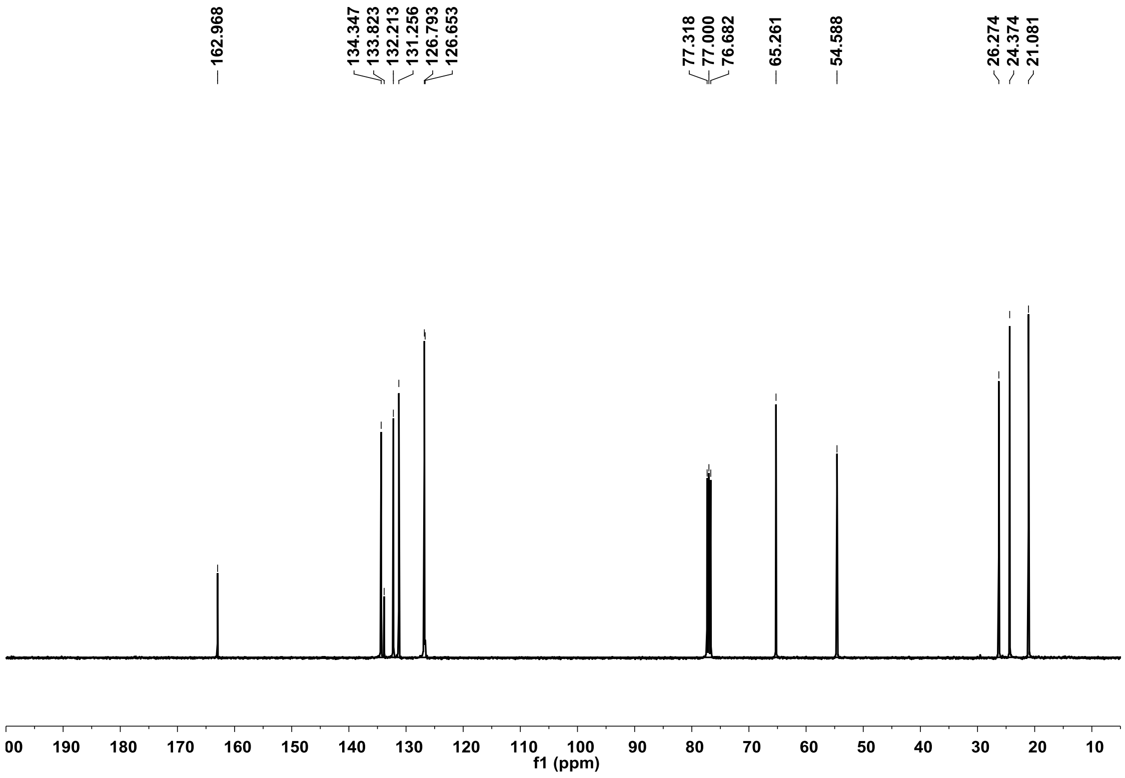
162.968
134.347 133.823 132.213 131.256 126.793 126.653
77.318 77.000 76.682
65.261
54.588
26.274 24.374 21.081
f1 (ppm)

3ab

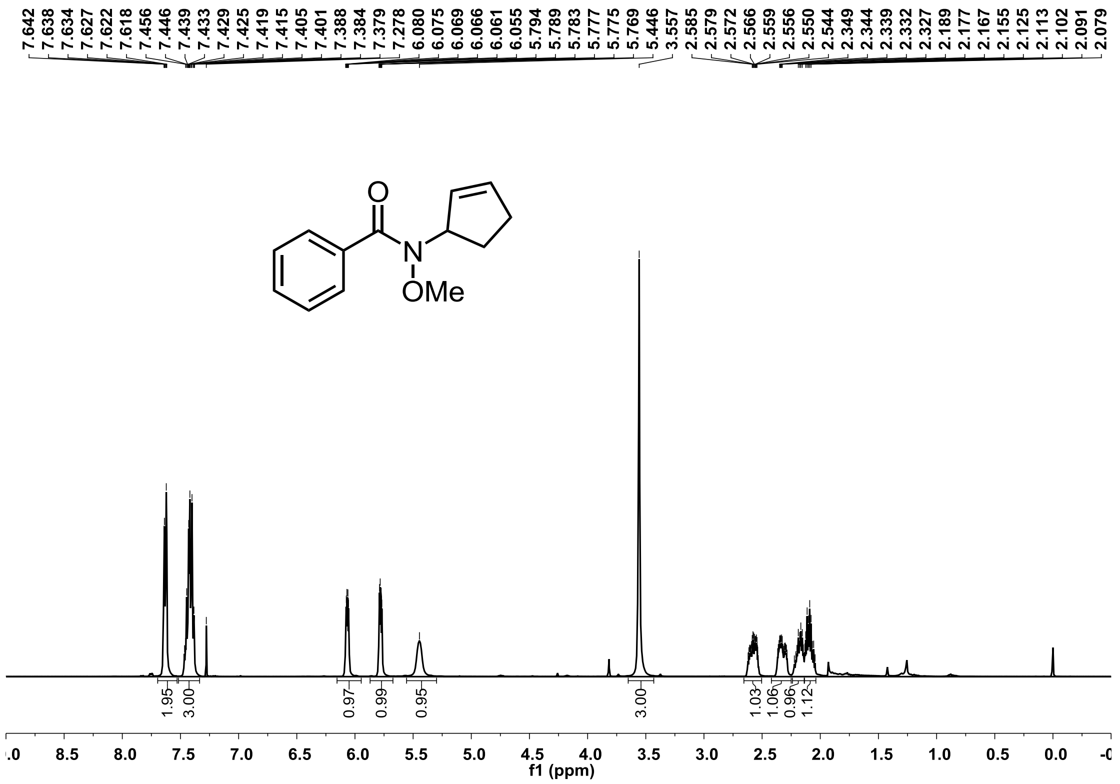
7.642 7.638 7.634 7.627 7.622 7.618 7.456 7.446 7.439 7.433 7.429 7.425 7.419 7.415 7.405 7.401 7.388 7.384 7.379 7.278 6.080 6.075 6.069 6.066 6.061 6.055 5.794 5.789 5.783 5.777 5.775 5.769 5.446 3.557 2.585 2.579 2.572 2.566 2.559 2.556 2.550 2.544 2.349 2.344 2.339 2.332 2.327 2.189 2.177 2.167 2.155 2.125 2.113 2.102 2.091 2.079
O
N
OMe
1.95 3.00 0.97 0.99 0.95 3.00 1.03 1.06 0.96 1.12
8.5 8.0 7.5 7.0 6.5 6.0 5.5 5.0 4.5 4.0 3.5 3.0 2.5 2.0 1.5 1.0 0.5 0.0
f1 (ppm)

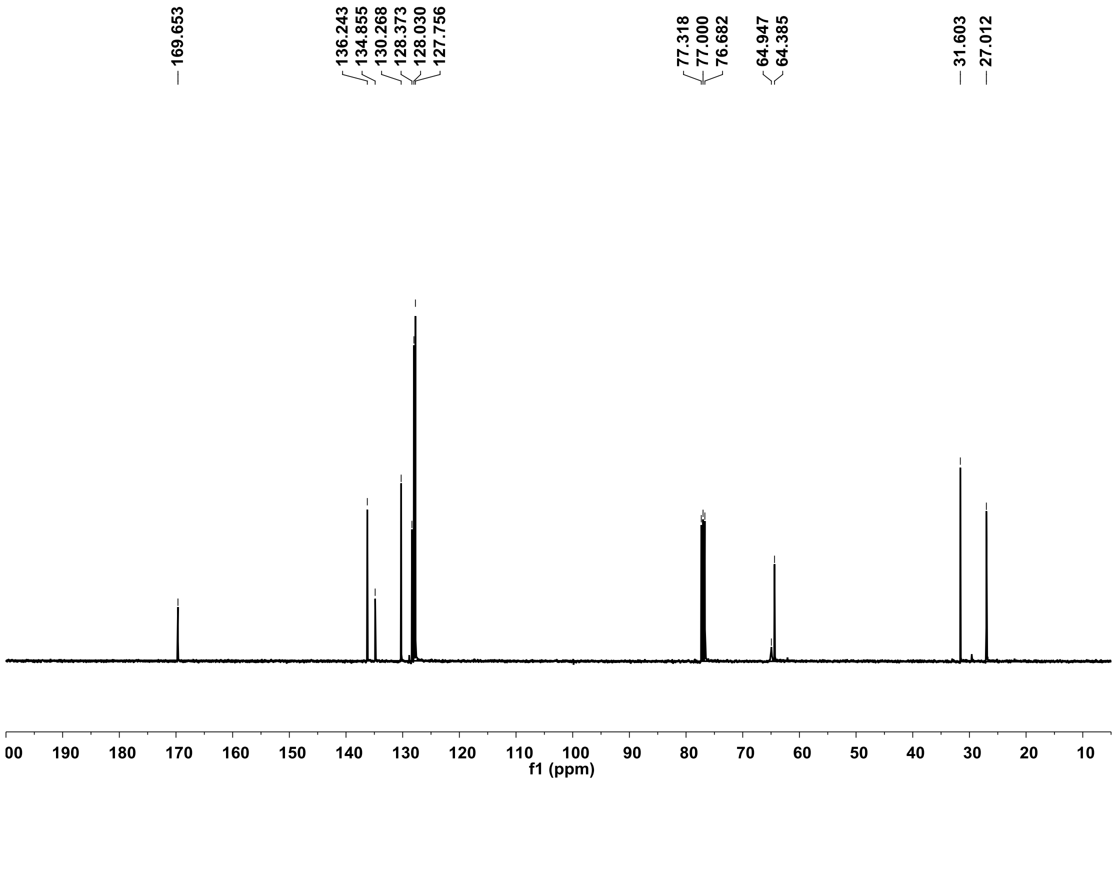
169.653
136.243 134.855 130.268 128.373 128.030 127.756
77.318 77.000 76.682
64.947 64.385
31.603 27.012
190 180 170 160 150 140 130 120 110 100 90 80 70 60 50 40 30 20 10
f1 (ppm)

3ac

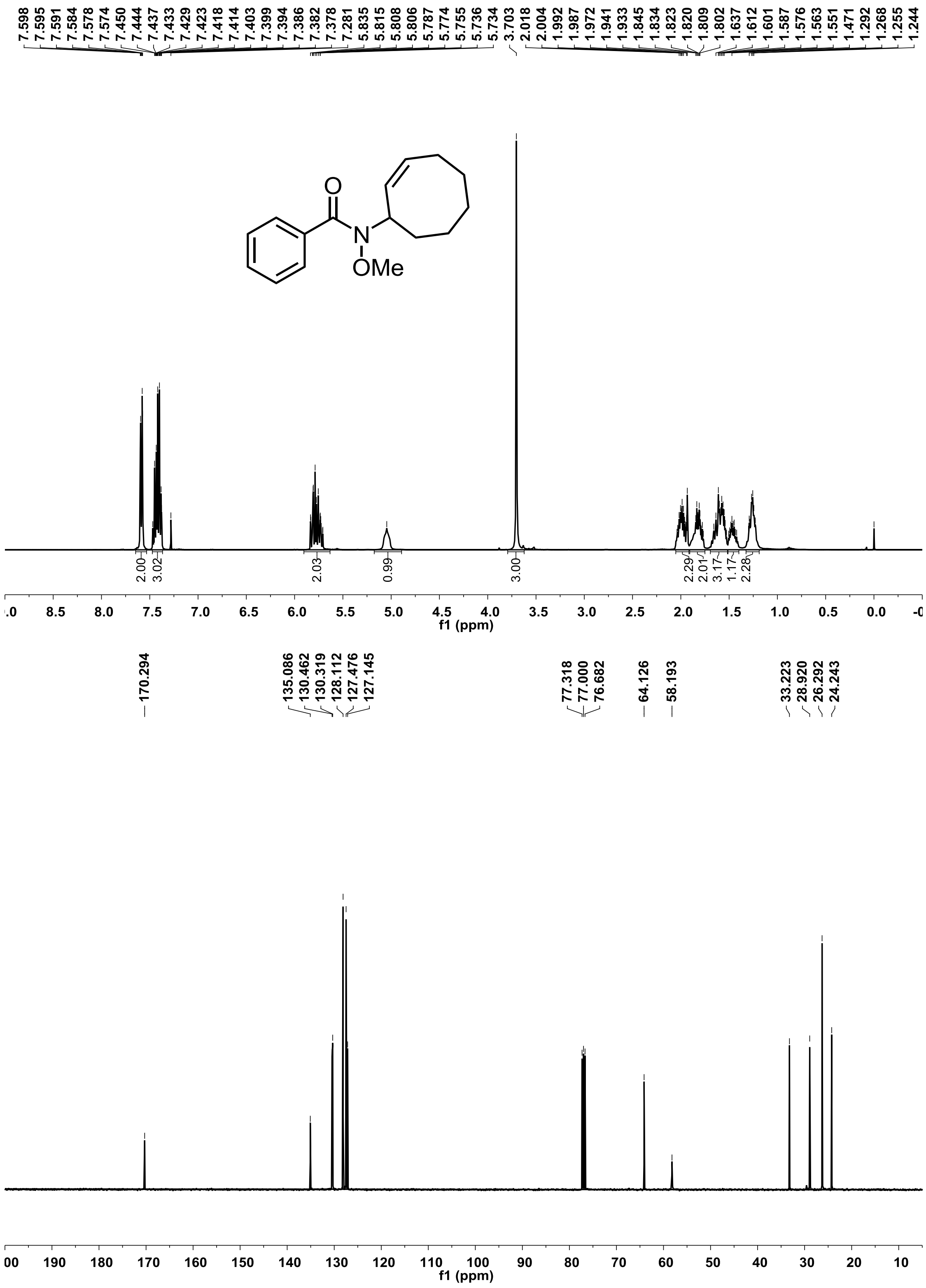
7.598
7.595
7.591
7.584
7.578
7.574
7.450
7.444
7.437
7.433
7.429
7.423
7.418
7.414
7.403
7.399
7.394
7.386
7.382
7.378
7.281
5.835
5.815
5.808
5.806
5.787
5.774
5.755
5.736
5.734
3.703
2.018
2.004
1.992
1.987
1.972
1.941
1.933
1.845
1.834
1.823
1.820
1.809
1.802
1.637
1.612
1.601
1.587
1.576
1.563
1.551
1.471
1.292
1.268
1.255
1.244
O
N
OMe
2.00
3.02
2.03
0.99
3.00
2.29
2.01
3.17
1.17
2.28
f1 (ppm)
170.294
135.086
130.462
130.319
128.112
127.476
127.145
77.318
77.000
76.682
64.126
58.193
33.223
28.920
26.292
24.243
f1 (ppm)

3ad

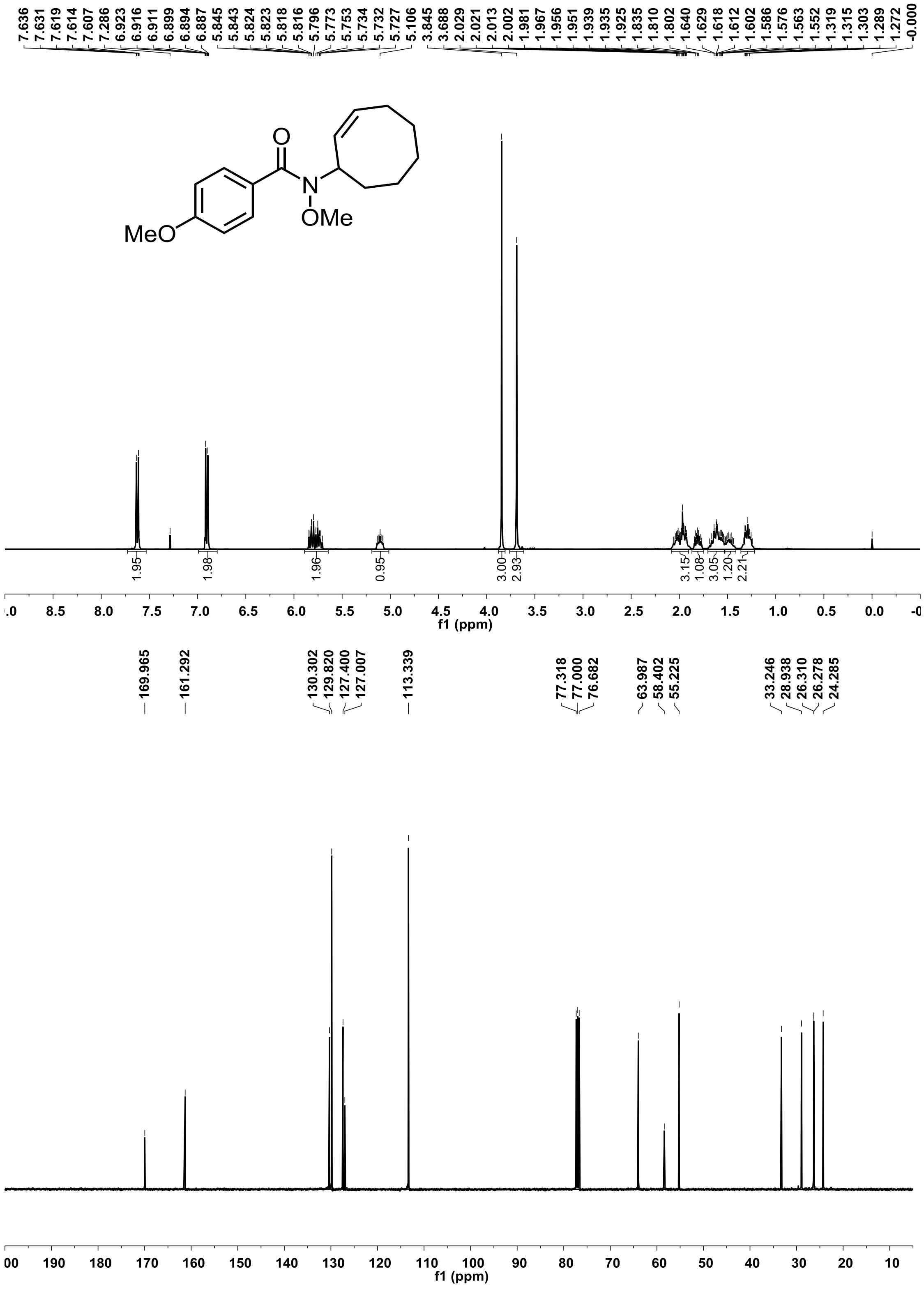
7.636
7.631
7.619
7.614
7.607
7.286
6.923
6.916
6.911
6.899
6.894
6.887
5.845
5.843
5.824
5.823
5.818
5.816
5.796
5.773
5.753
5.734
5.732
5.727
5.106
3.845
3.688
2.029
2.021
2.013
2.002
1.981
1.967
1.956
1.951
1.939
1.935
1.925
1.835
1.810
1.802
1.640
1.629
1.618
1.612
1.602
1.586
1.576
1.563
1.552
1.319
1.315
1.303
1.289
1.272
-0.000
MeO
O
N
OMe
1.95
1.98
1.96
0.95
3.00
2.93
3.15
1.08
3.05
1.20
2.21
f1 (ppm)
169.965
161.292
130.302
129.820
127.400
127.007
113.339
77.318
77.000
76.682
63.987
58.402
55.225
33.246
28.938
26.310
26.278
24.285
f1 (ppm)

3ae

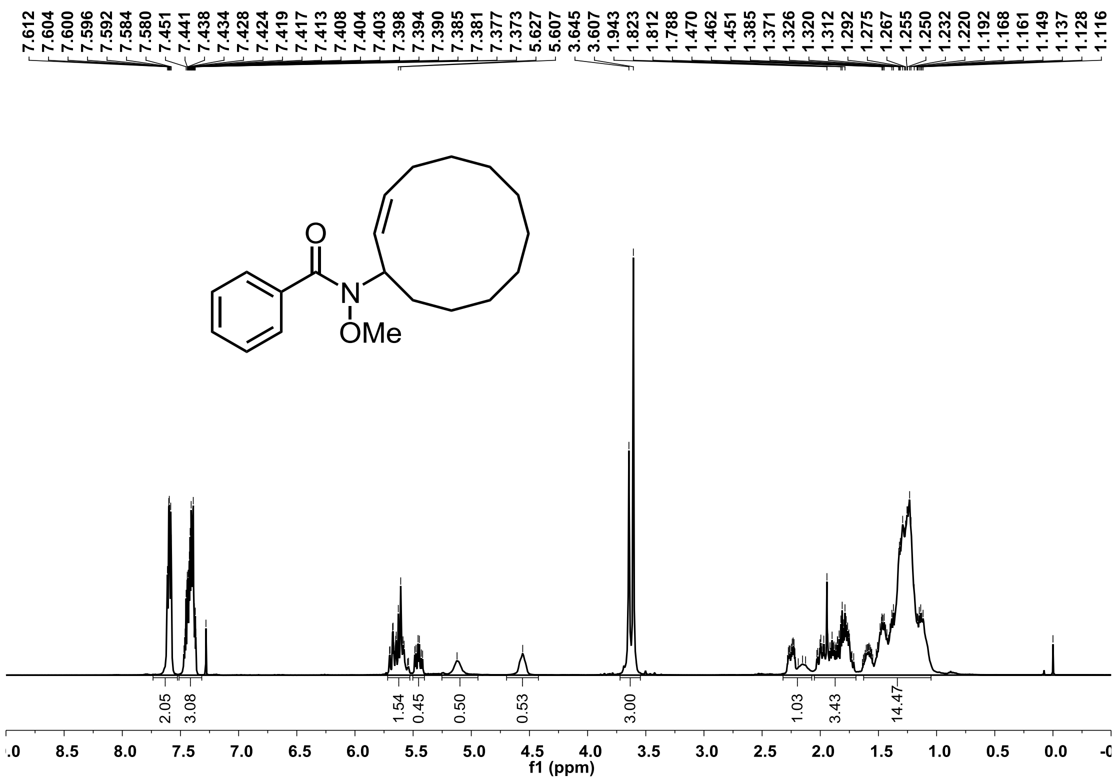

7.612
7.604
7.600
7.596
7.592
7.584
7.580
7.451
7.441
7.438
7.434
7.428
7.424
7.419
7.417
7.413
7.408
7.404
7.403
7.398
7.394
7.390
7.385
7.381
7.377
7.373
5.627
5.607
3.645
3.607
1.943
1.823
1.812
1.788
1.470
1.462
1.451
1.385
1.371
1.326
1.320
1.312
1.292
1.275
1.267
1.255
1.250
1.232
1.220
1.192
1.168
1.161
1.149
1.137
1.128
1.116
O
N
OMe
2.05
3.08
1.54
0.45
0.50
0.53
3.00
1.03
3.43
14.47
8.5 8.0 7.5 7.0 6.5 6.0 5.5 5.0 4.5 4.0 3.5 3.0 2.5 2.0 1.5 1.0 0.5 0.0
f1 (ppm)

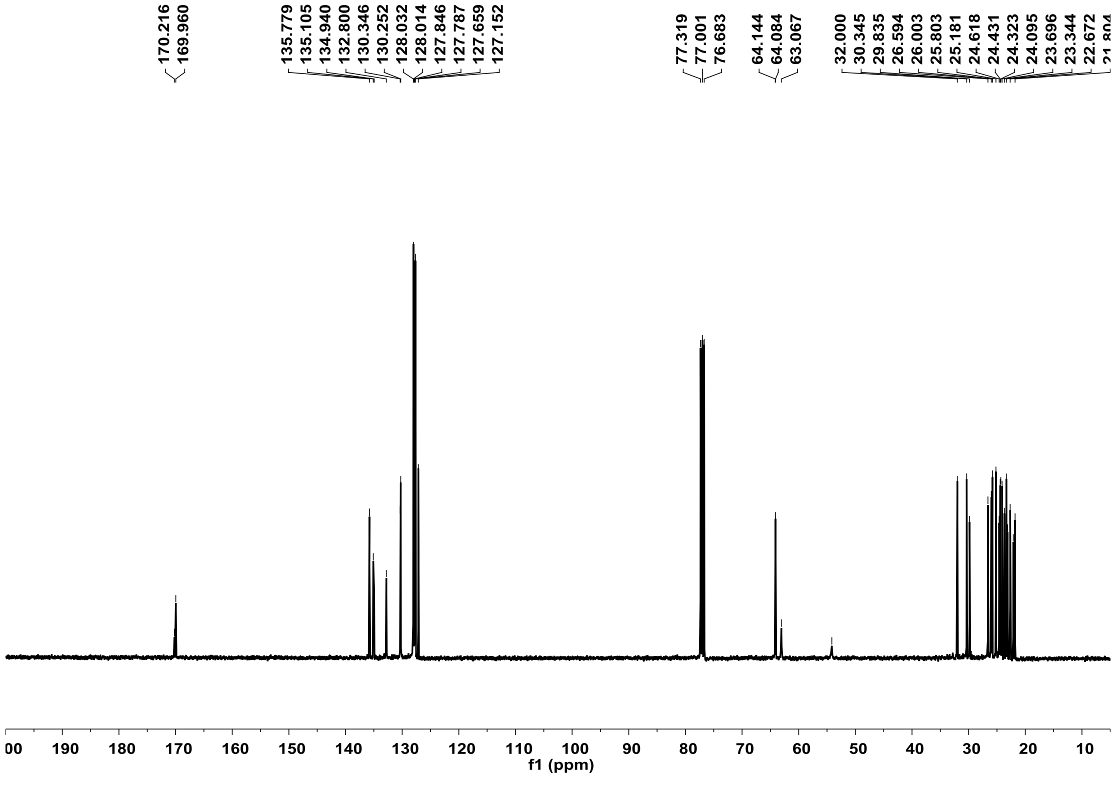

170.216
169.960
135.779
135.105
134.940
132.800
130.346
130.252
128.032
128.014
127.846
127.787
127.659
127.152
77.319
77.001
76.683
64.144
64.084
63.067
32.000
30.345
29.835
26.594
26.003
25.803
25.181
24.618
24.431
24.323
24.095
23.696
23.344
22.672
190 180 170 160 150 140 130 120 110 100 90 80 70 60 50 40 30 20 10
f1 (ppm)

3af

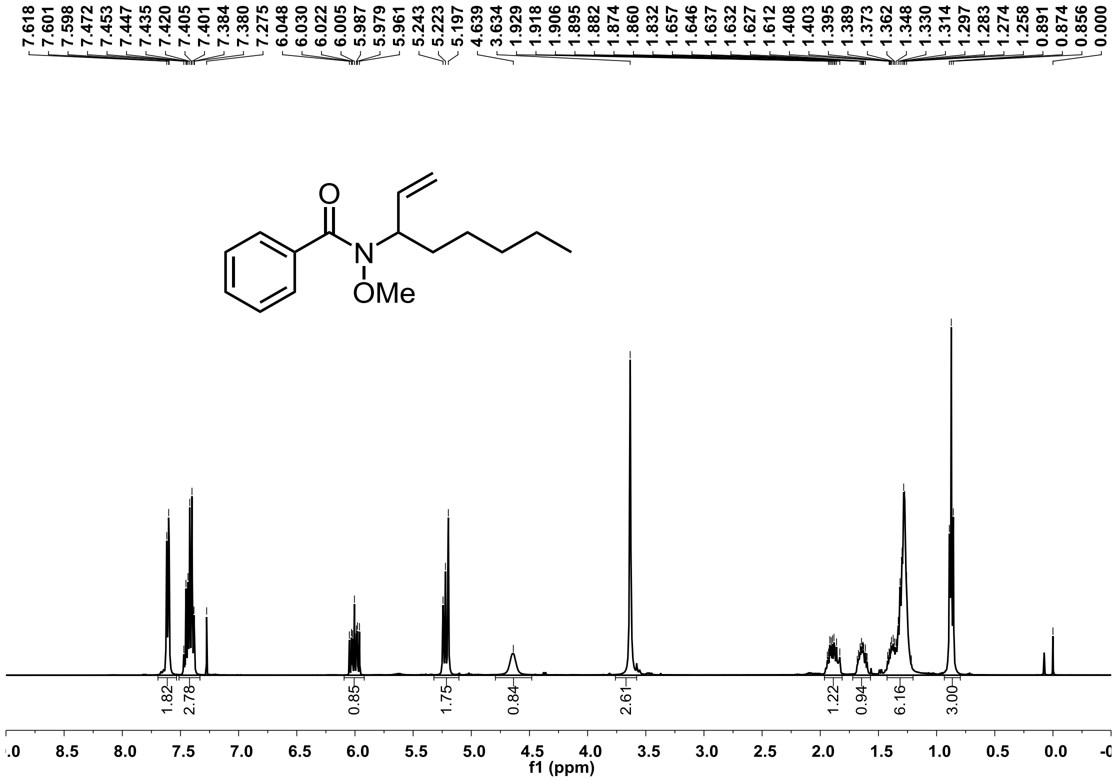
7.618 7.601 7.598 7.472 7.453 7.447 7.435 7.420 7.405 7.401 7.384 7.380 7.275 6.048 6.030 6.022 6.005 5.987 5.979 5.961 5.243 5.223 5.197 4.639 3.634 1.929 1.918 1.906 1.895 1.882 1.874 1.860 1.832 1.657 1.646 1.637 1.632 1.627 1.612 1.408 1.403 1.395 1.389 1.373 1.362 1.348 1.330 1.314 1.297 1.283 1.274 1.258 0.891 0.874 0.856 0.000
O
N
OMe
1.82 2.78 0.85 1.75 0.84 2.61 1.22 0.94 6.16 3.00
f1 (ppm)

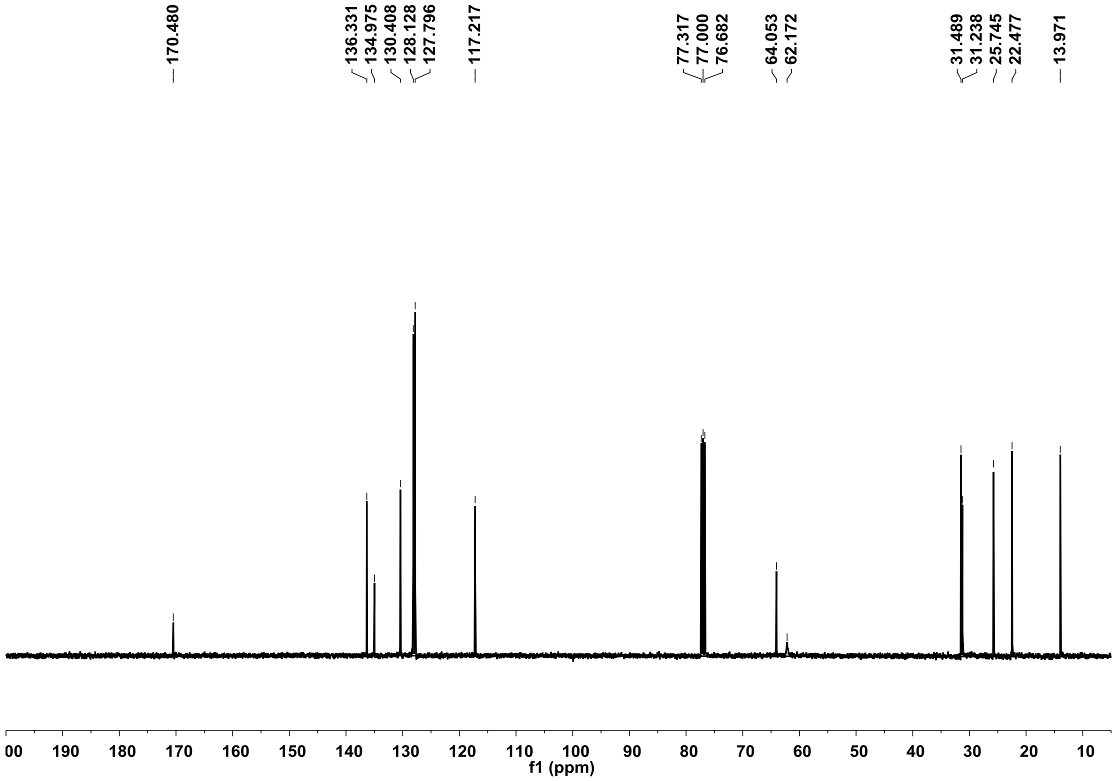
170.480 136.331 134.975 130.408 128.128 127.796 117.217 77.317 77.000 76.682 64.053 62.172 31.489 31.238 25.745 22.477 13.971
f1 (ppm)

3ag

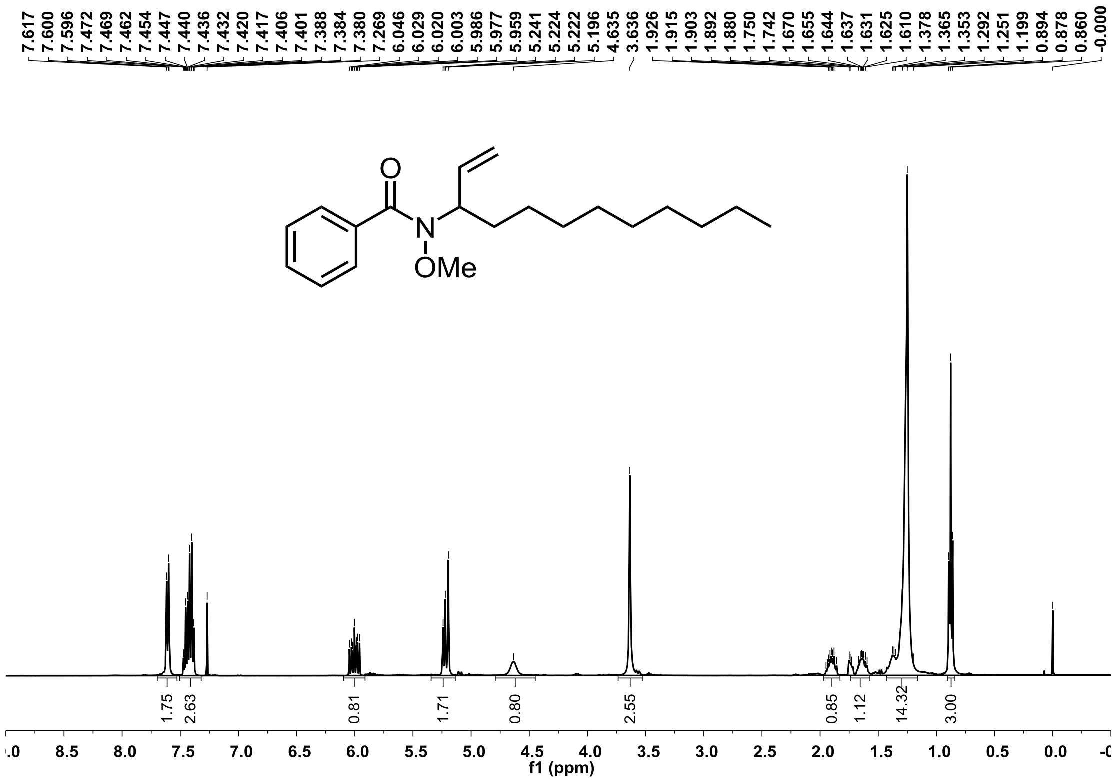
7.617 7.600 7.596 7.472 7.469 7.462 7.454 7.447 7.440 7.436 7.432 7.420 7.417 7.406 7.401 7.388 7.384 7.380 7.269 6.046 6.029 6.020 6.003 5.986 5.977 5.959 5.241 5.224 5.222 5.196 4.635 3.636 1.926 1.915 1.903 1.892 1.880 1.750 1.742 1.670 1.655 1.644 1.637 1.631 1.625 1.610 1.378 1.365 1.353 1.292 1.251 1.199 0.894 0.878 0.860 -0.000
O
N
OMe
1.75 2.63 0.81 1.71 0.80 2.55 0.85 1.12 14.32 3.00
8.5 8.0 7.5 7.0 6.5 6.0 5.5 5.0 4.5 4.0 3.5 3.0 2.5 2.0 1.5 1.0 0.5 0.0
f1 (ppm)

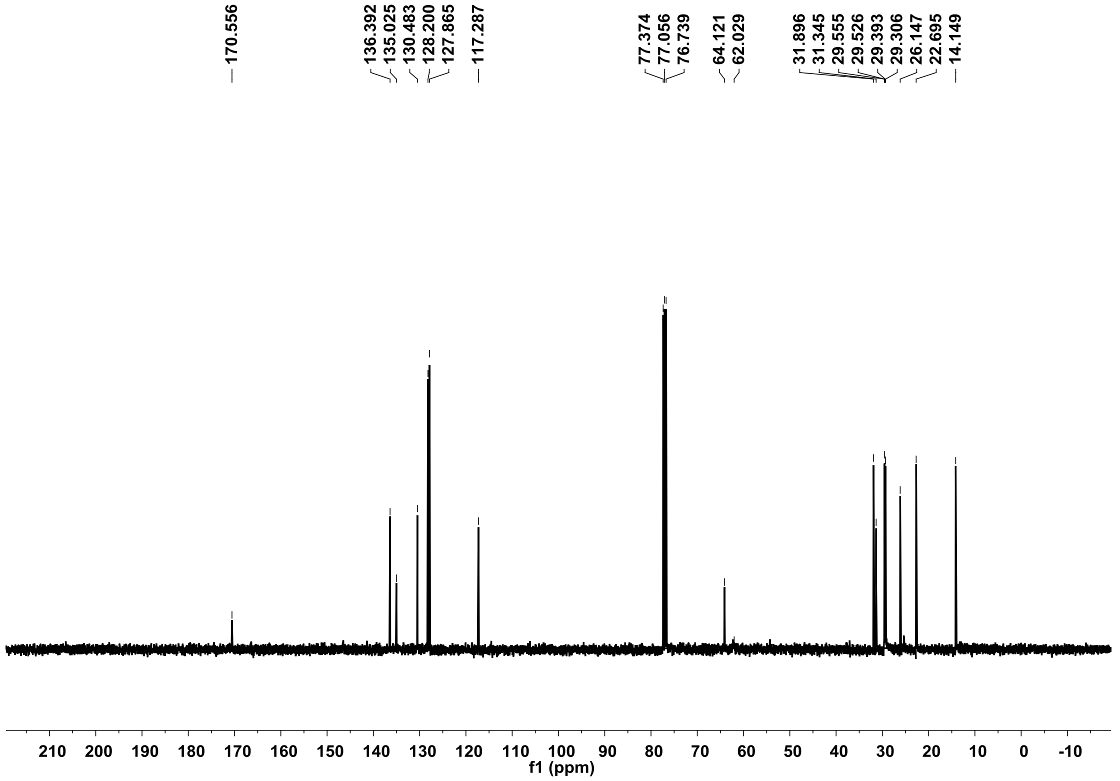
170.556
136.392 135.025 130.483 128.200 127.865 117.287
77.374 77.056 76.739 64.121 62.029
31.896 31.345 29.555 29.526 29.393 29.306 26.147 22.695 14.149
210 200 190 180 170 160 150 140 130 120 110 100 90 80 70 60 50 40 30 20 10 0 -10
f1 (ppm)

3ah

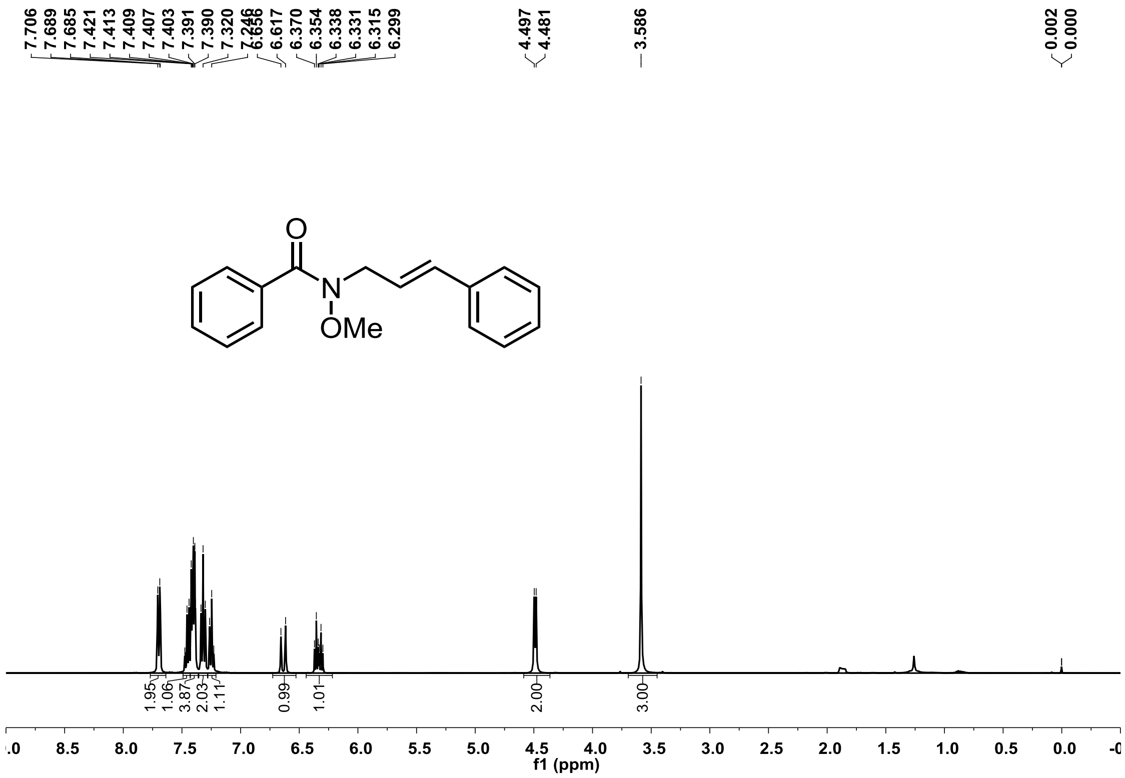
7.706
7.689
7.685
7.421
7.413
7.409
7.407
7.403
7.391
7.390
7.320
6.617
6.370
6.354
6.338
6.331
6.315
6.299
4.497
4.481
3.586
0.002
0.000
O
N
OMe
1.95
1.06
3.87
2.03
1.11
0.99
1.01
2.00
3.00
8.5
8.0
7.5
7.0
6.5
6.0
5.5
5.0
4.5
4.0
3.5
3.0
2.5
2.0
1.5
1.0
0.5
0.0
f1 (ppm)

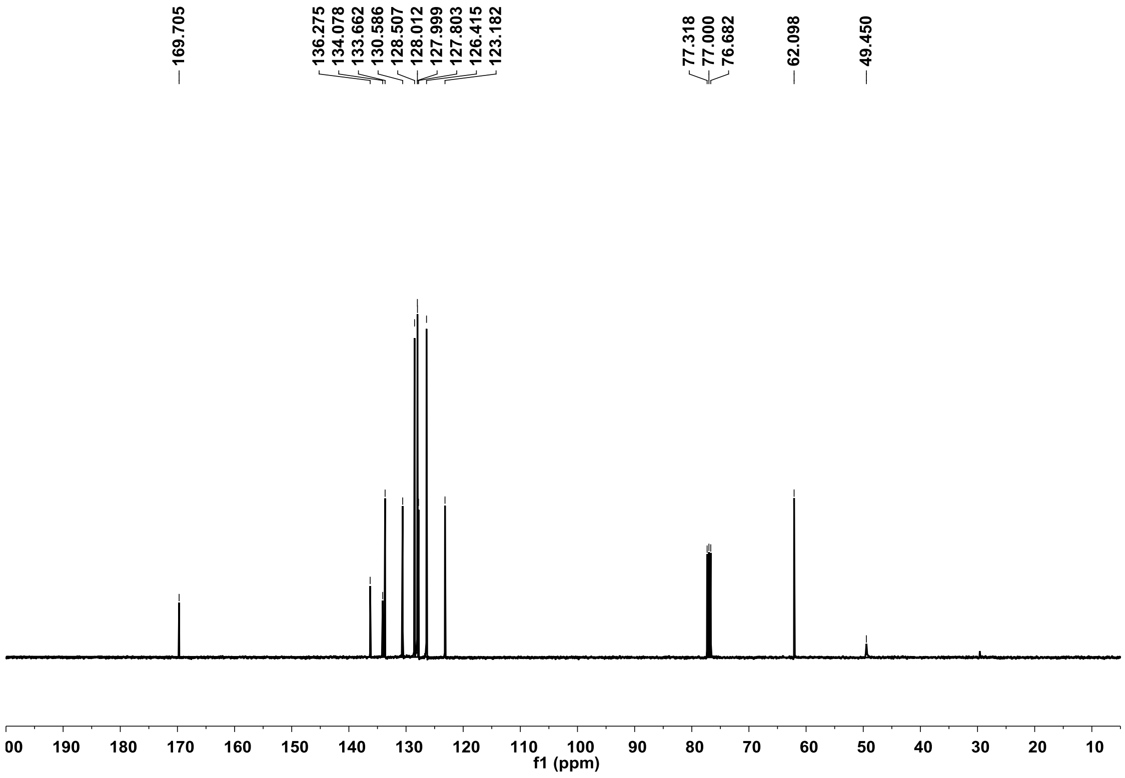
169.705
136.275
134.078
133.662
130.586
128.507
128.012
127.999
127.803
126.415
123.182
77.318
77.000
76.682
62.098
49.450
190
180
170
160
150
140
130
120
110
100
90
80
70
60
50
40
30
20
10
f1 (ppm)

3ai

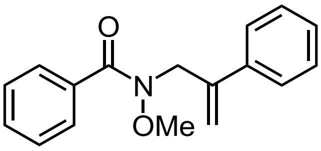
O
N
OMe

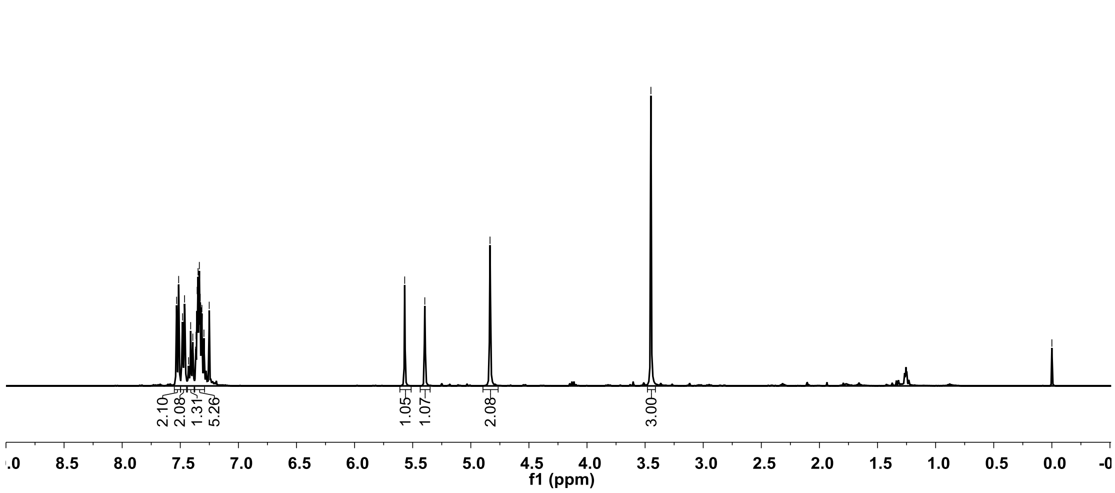
7.531
7.514
7.510
7.484
7.481
7.463
7.411
7.364
7.355
7.351
7.347
7.340
7.336
7.332
7.328
7.318
7.314
7.296
7.251
5.569
5.396
4.835
3.450
-0.000
2.10
2.08
1.31
5.26
1.05
1.07
2.08
3.00
f1 (ppm)

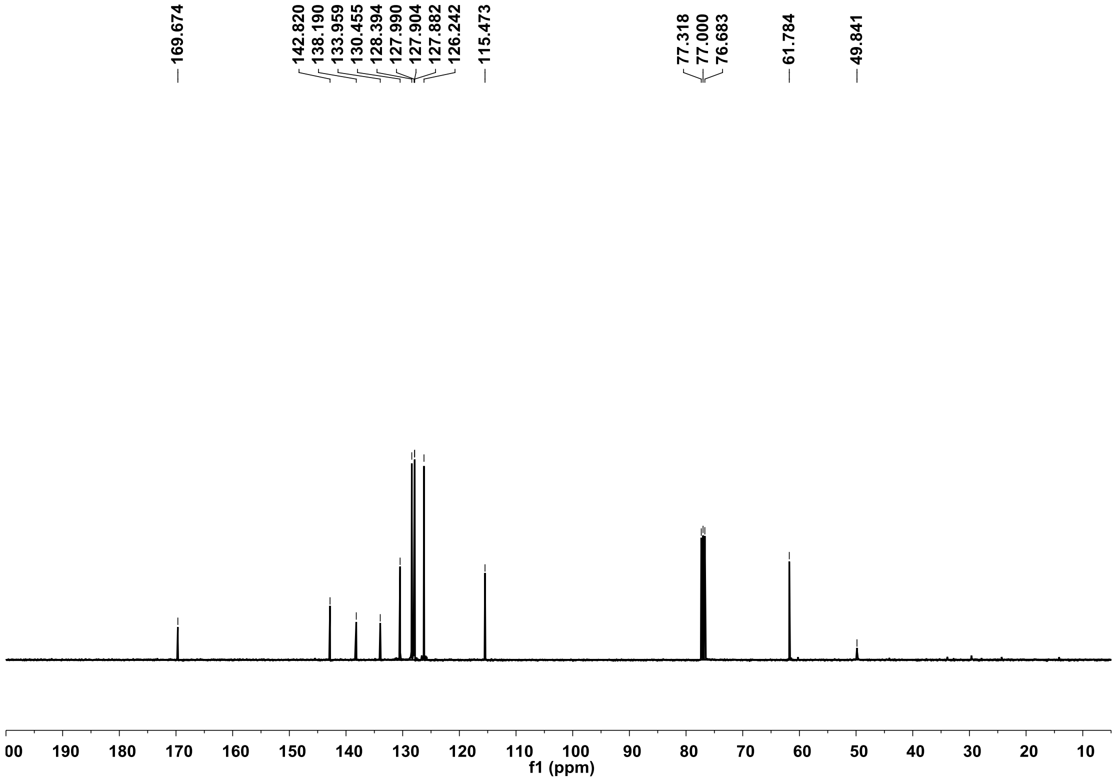
169.674
142.820
138.190
133.959
130.455
128.394
127.990
127.904
127.882
126.242
115.473
77.318
77.000
76.683
61.784
49.841
f1 (ppm)

Complete reference for Gaussian 09

Gaussian 09, Revision A.2, Frisch, M. J.; Trucks, G. W.; Schlegel, H. B.; Scuseria, G. E.; Robb, M. A.; Cheeseman, J. R.; Scalmani, G.; Barone, V.; Mennucci, B.; Petersson, G. A.; Nakatsuji, H.; Caricato, M.; Li, X.; Hratchian, H. P.; Izmaylov, A. F.; Bloino, J.; Zheng, G.; Sonnenberg, J. L.; Hada, M.; Ehara, M.; Toyota, K.; Fukuda, R.; Hasegawa, J.; Ishida, M.; Nakajima, T.; Honda, Y.; Kitao, O.; Nakai, H.; Vreven, T.; Montgomery, Jr., J. A.; Peralta, J. E.; Ogliaro, F.; Bearpark, M.; Heyd, J. J.; Brothers, E.; Kudin, K. N.; Staroverov, V. N.; Kobayashi, R.; Normand, J.; Raghavachari, K.; Rendell, A.; Burant, J. C.; Iyengar, S. S.; Tomasi, J.; Cossi, M.; Rega, N.; Millam, N. J.; Klene, M.; Knox, J. E.; Cross, J. B.; Bakken, V.; Adamo, C.; Jaramillo, J.; Gomperts, R.; Stratmann, R. E.; Yazyev, O.; Austin, A. J.; Cammi, R.; Pomelli, C.; Ochterski, J. W.; Martin, R. L.; Morokuma, K.; Zakrzewski, V. G.; Voth, G. A.; Salvador, P.; Dannenberg, J. J.; Dapprich, S.; Daniels, A. D.; Farkas, Ö.; Foresman, J. B.; Ortiz, J. V.; Cioslowski, J.; Fox, D. J. Gaussian, Inc., Wallingford CT, **2009**.

B3LYP absolute calculation energies, enthalpies, and free energies

All the density functional theory (DFT) calculations were carried out with the GAUSSIAN 09 series of programs. DFT method B3LYP with a standard 6-31G(d) basis set was used for geometry optimizations. Harmonic frequency calculations were performed for all stationary points to confirm them as a local minima or transition structures and to derive the thermo chemical corrections for the enthalpies and free energies.

Geometry	E(B3LYP)	H(B3LYP)	G(B3LYP)	IF*
**1a**	-515.400833	-515.229630	-515.277331	-
**2a**	-234.643975	-234.490514	-234.525590	-
**1a-ts**	-748.406891	-748.108626	-748.175013	-1055.56
**6**	-514.759509	-514.601589	-514.649816	-
**6a**	-2635.006712	-2634.867563	-2634.769995	-
**7**	-2634.974691	-2634.738428	-2634.835708	-
**8**	-2635.013756	-2634.777006	-2634.876681	-
**2a-ts**	-467.639850	-467.359604	-467.414731	-1255.76
**9**	-234.003753	-233.864264	-233.899875	-
**9a**	-2354.228925	-2354.010933	-2354.097454	-
**9b**	-2354.241708	-2354.021866	-2354.106086	-
**10**	-2869.095080	-2868.712344	-2868.822991	-

* IF: The imaginary frequencies for the transition states.

B3LYP geometries for all the optimized compounds and transition states

1a

N	2.07906100	0.55472800	-0.07560200
C	0.83831800	1.17229100	-0.17772300
O	0.83509200	2.39766400	-0.22845000
C	-0.43587000	0.38322200	-0.09898900
C	-0.59950000	-0.94954400	-0.50972100
C	-1.55185000	1.09327100	0.37558200
C	-1.85446800	-1.55615000	-0.42976000
H	0.24248400	-1.50005200	-0.90677100
C	-2.79701000	0.47861200	0.46887900
H	-1.41751400	2.13069800	0.66209900
C	-2.95160400	-0.85075500	0.06611800
H	-1.97323300	-2.58453900	-0.76053200
H	-3.64828400	1.03682700	0.84893400
H	-3.92422500	-1.33140500	0.13211900
O	2.28935800	-0.79362400	-0.39884200
C	2.68148600	-1.51514300	0.77388600
H	2.85597000	-2.53949100	0.43441300
H	3.60450400	-1.10226700	1.19819700
H	1.88904200	-1.49895400	1.53013900
H	2.84522200	1.13894000	-0.40068500

2a

C	0.66623700	1.30622500	0.05748300
C	-0.66618800	1.30624700	-0.05748400
C	-1.49914200	0.04781200	-0.11081500
C	-0.69823800	-1.19235400	0.31915200
C	0.69819800	-1.19236900	-0.31916600

C	1.49914100	0.04775900	0.11083300
H	-2.38655100	0.16403700	0.52699800
H	-0.59255900	-1.19173900	1.41307300
H	-1.24474900	-2.10619300	0.05544400
H	1.24467800	-2.10623400	-0.05548400
H	0.59251700	-1.19171900	-1.41308600
H	1.88937400	-0.09085000	1.13196100
H	2.38657800	0.16394700	-0.52694600
H	-1.88942000	-0.09077600	-1.13192800
H	1.19972500	2.25478800	0.11311500
H	-1.19964300	2.25482600	-0.11316800

1a-ts

C	0.68198700	1.51504800	0.32990700
C	1.73632600	0.50033900	0.01571000
C	1.67041300	-0.38694600	-1.07079800
C	2.85757800	0.47595200	0.85932800
C	2.71066200	-1.28794100	-1.29779800
H	0.81462500	-0.38278400	-1.73563500
C	3.89199800	-0.42681200	0.62991700
H	2.89545300	1.17683800	1.68660400
C	3.82032100	-1.31151300	-0.45003700
H	2.65490100	-1.97001300	-2.14165400
H	4.75458900	-0.44078300	1.29042200
H	4.62847300	-2.01505100	-0.63198100
O	0.83398300	2.40174100	1.15403500
N	-0.47620400	1.42735300	-0.47004000
H	-0.92215100	0.40826800	-0.67403300
O	-1.44821400	2.30645900	-0.05711900
C	-2.38391400	2.51084000	-1.12154300

H	-2.82260800	1.55445800	-1.42645700
H	-3.14869200	3.16894200	-0.70581000
H	-1.88992400	2.98580200	-1.97593000
O	-1.61631300	-0.74718300	-0.82154700
C	-2.11813900	-1.44083300	0.28862100
C	-3.19522700	-0.60472500	1.00961800
C	-2.76445200	-2.71203700	-0.32542100
C	-0.99234400	-1.84621100	1.25713400
H	-2.76873500	0.33580500	1.37434300
H	-4.01618100	-0.36902000	0.32377900
H	-3.60622400	-1.14852700	1.86809400
H	-2.00943300	-3.31026500	-0.84415700
H	-3.21219700	-3.31732700	0.47107400
H	-3.54339100	-2.43320000	-1.04121500
H	-1.37720800	-2.47310900	2.06982200
H	-0.21304200	-2.40103400	0.72524900
H	-0.53173600	-0.95955700	1.70699400

6

N	2.08266900	0.54837800	-0.21332000
C	0.79718200	1.13136200	-0.23022300
O	0.78948200	2.35157700	-0.37026200
C	-0.47730800	0.35358400	-0.09853900
C	-0.63842800	-0.97853300	-0.51100200
C	-1.58949100	1.05583300	0.39373200
C	-1.88697200	-1.59382000	-0.42077000
H	0.20254000	-1.53028900	-0.91223300
C	-2.82968400	0.43257300	0.50136100
H	-1.46006800	2.09345600	0.68321200
C	-2.98130500	-0.89533000	0.09293500

H	-2.00438500	-2.62170700	-0.75304800
H	-3.67933300	0.98283100	0.89616400
H	-3.95007000	-1.38195000	0.16959500
O	2.04473700	-0.69584600	0.32160100
C	3.37064300	-1.24800900	0.37514600
H	3.25461800	-2.24371000	0.80578200
H	3.79153200	-1.30575000	-0.63237800
H	4.00490600	-0.62334200	1.00959400

6a

S	0.38913800	2.82216600	-0.45141700
O	-0.08435600	2.67580300	0.94601400
O	0.05851600	4.01832400	-1.20994300
S	-3.35742400	-0.15446100	0.47578200
O	-4.44398000	0.31317900	1.31509700
O	-2.10985300	-0.70007600	1.18199300
C	2.24924600	2.79537300	-0.32649100
C	-3.99508100	-1.61470500	-0.49779300
F	2.63867600	1.74290300	0.42865400
F	2.67802900	3.91218800	0.26057500
F	2.80379100	2.68148000	-1.53082600
F	-3.02226300	-2.08684200	-1.27937600
F	-4.38773100	-2.56486200	0.34866000
F	-5.02121600	-1.22164000	-1.24650600
O	0.11822000	1.50404900	-1.20440900
O	-2.77443800	0.78261200	-0.56384900
Cu	-0.84416200	0.30902400	-0.01632100
N	0.81859500	-0.62096800	0.69860100
C	1.30011300	-1.55034200	-0.26672800
O	0.49407000	-1.72789400	-1.17563800

C	2.61482400	-2.21499300	-0.16808900
C	3.72117300	-1.63997100	0.48067300
C	2.75423200	-3.44634700	-0.83232900
C	4.94665500	-2.30236700	0.47084400
H	3.63603100	-0.67120900	0.95709000
C	3.97661100	-4.10961100	-0.82015100
H	1.89677300	-3.86564500	-1.34816700
C	5.07372000	-3.53823400	-0.16798200
H	5.80487500	-1.85076400	0.95940500
H	4.07747800	-5.06672400	-1.32302100
H	6.03032400	-4.05320800	-0.16462600
O	1.39620700	-0.67939300	1.89157000
C	0.76738100	0.19099000	2.87919000
H	1.39498000	0.08614300	3.76302000
H	0.75663100	1.21384200	2.49923900
H	-0.24589700	-0.17127600	3.06615600

7

S	-0.66655400	2.63381000	0.49851800
O	-1.41845800	3.62169500	1.25511500
O	-1.27829200	1.99369200	-0.70502400
S	-2.54813800	-1.74889100	-0.03762700
O	-2.81040200	-3.14573300	0.27530700
O	-1.34164000	-1.47512100	-0.94339400
C	0.85800200	3.50467000	-0.12854600
C	-4.00355100	-1.12593400	-1.02564200
F	1.55987000	3.98335800	0.89967800
F	0.49588400	4.50804700	-0.92771700
F	1.62213400	2.64826900	-0.81862700
F	-3.81402800	0.14961200	-1.35120300

F	-4.12344400	-1.85953300	-2.13175500
F	-5.10623200	-1.24285400	-0.28668300
O	-0.07707100	1.47530200	1.32526400
O	-2.38574100	-0.77864300	1.10413100
Cu	-0.55800300	-0.02091300	0.14754000
N	1.88381500	-1.76508400	1.41925200
C	2.17616400	-1.11763700	0.20931800
O	1.35548000	-0.43032200	-0.43089100
C	3.55572800	-1.31010100	-0.25991800
C	4.46896200	-2.12063000	0.44309500
C	3.95886300	-0.66088100	-1.44222200
C	5.76587900	-2.27574400	-0.03701000
H	4.15650000	-2.61927700	1.35283700
C	5.25635400	-0.82094400	-1.91248800
H	3.24499000	-0.03792100	-1.96998200
C	6.16051000	-1.62824300	-1.21133500
H	6.47002100	-2.90110000	0.50343200
H	5.56757700	-0.31898200	-2.82360900
H	7.17435000	-1.75152500	-1.58196100
O	0.61099100	-1.53416800	1.73557500
C	0.22126100	-2.14803100	2.98881900
H	-0.84341300	-1.93715500	3.07424100
H	0.41563600	-3.22055500	2.93284100
H	0.78916100	-1.68495600	3.79819000

8

S	0.22292500	2.15918100	0.94816200
O	0.25487000	2.92926500	2.18393300
O	-0.82024200	2.37397200	-0.06087800
S	-3.30083500	-0.97412300	-0.01404100

O	-4.28542000	-1.94409900	0.43207200
O	-2.11991400	-1.50089300	-0.83341400
C	1.84429400	2.48720500	0.08667200
C	-4.16871600	0.21739600	-1.16197900
F	2.86261600	2.08284200	0.84803700
F	1.95883600	3.79295800	-0.15333900
F	1.87689700	1.82295000	-1.07652500
F	-3.28724600	1.08830900	-1.64674600
F	-4.71536600	-0.47300800	-2.16098500
F	-5.11478700	0.85908700	-0.48276400
O	0.36332400	0.62686500	1.23703200
O	-2.62139200	-0.10741500	1.02879600
Cu	-0.75464400	-0.44312200	0.16474800
N	1.69556800	-2.12418100	1.21473900
C	1.76136400	-1.60933100	-0.03696100
O	0.71927600	-1.32598300	-0.72053700
C	3.11788600	-1.38336300	-0.57124100
C	4.26483100	-1.61184100	0.21281200
C	3.25749500	-0.94255700	-1.89807400
C	5.52913900	-1.40815000	-0.33190500
H	4.15637200	-1.94429300	1.23864600
C	4.52518100	-0.73931700	-2.43374200
H	2.36816500	-0.76959600	-2.49382700
C	5.66281500	-0.97152700	-1.65307900
H	6.41173700	-1.58445500	0.27588300
H	4.62883600	-0.40239200	-3.46097600
H	6.65145200	-0.81212400	-2.07443200
O	0.42135100	-2.23537600	1.60509900
C	0.31898700	-2.53868900	3.01322600
H	-0.72992300	-2.78671900	3.17309100

H	0.97182800	-3.38043800	3.25196400
H	0.60078100	-1.64900900	3.58252900

2a-ts

C	1.50157200	1.25826900	0.65626100
C	2.44970700	1.31895800	-0.30159400
C	3.08038400	0.09617700	-0.91566100
C	2.29445500	-1.18831200	-0.60226600
C	1.85898900	-1.23132400	0.86923300
C	0.99694500	-0.01575400	1.19866100
H	3.17027100	0.23276000	-2.00244900
H	1.39130200	-1.22450000	-1.22407100
H	2.89632800	-2.06943600	-0.85445400
H	1.29984700	-2.15032200	1.07753500
H	2.74371700	-1.23734400	1.52513900
H	-0.08384600	-0.29358700	0.66361500
H	0.70663200	0.04645400	2.25353300
H	4.11650400	0.00920600	-0.54798600
H	1.08218000	2.18234100	1.05204300
H	2.80471900	2.29023600	-0.64377300
O	-1.16160000	-0.91779400	0.13107600
C	-2.19697500	-0.00075700	-0.14168100
C	-2.59703700	0.77221400	1.12950500
C	-1.80082800	0.96186800	-1.27672100
C	-3.36997300	-0.90136700	-0.59642900
H	-2.83168000	0.07165300	1.93776600
H	-1.77610200	1.41768000	1.46392500
H	-3.47245300	1.40823800	0.95227900
H	-1.52878600	0.39338200	-2.17260700
H	-2.62247100	1.64209000	-1.53244700

H	-0.93553200	1.56776200	-0.98625900
H	-4.23878900	-0.28450400	-0.85480800
H	-3.08056500	-1.48655400	-1.47486100
H	-3.65191100	-1.59304100	0.20342200

9

C	1.21403800	0.82270500	0.00347300
C	-0.00780000	1.47878700	-0.09198000
C	-1.22264700	0.80986800	0.00342800
C	-1.27074300	-0.68146900	0.19657000
C	0.00715800	-1.35950600	-0.33126100
C	1.27787300	-0.66810600	0.19652000
H	-1.39484800	-0.91438900	1.26887000
H	-2.15485400	-1.10430800	-0.29927500
H	0.01271600	-2.42313800	-0.06448900
H	0.00684600	-1.30266100	-1.42754700
H	1.40451400	-0.89975900	1.26879300
H	2.16635700	-1.08154600	-0.29944100
H	-2.15610500	1.36533300	-0.03811800
H	2.14161600	1.38793600	-0.03797000
H	-0.01351400	2.55886200	-0.23131800

9a

S	-2.59467900	-0.21328000	0.00027000
O	-3.81328800	0.57462600	0.01918900
O	-1.66590400	-0.11978000	1.20776100
S	2.24537800	-1.39431800	-0.04017100
O	1.46285300	-0.77089400	-1.19216400
O	2.62037300	-2.79131300	-0.15064400
C	-3.09286800	-2.01372300	-0.06315600

C	3.83752700	-0.42086300	0.03592300
F	-3.80645800	-2.22873200	-1.16389200
F	-3.81579500	-2.30369900	1.01406700
F	-1.99341100	-2.77274100	-0.08460200
F	4.53293800	-0.80988600	1.09974200
F	4.53469000	-0.64352200	-1.07382400
F	3.56749500	0.88649700	0.13495300
O	-1.64556400	-0.03706600	-1.17804600
O	1.45320700	-0.95688100	1.18545000
Cu	-0.00650100	-0.08529300	0.03329500
C	0.81427500	2.58490600	-0.71167300
C	0.66868500	2.32797900	0.65674400
C	-0.35887800	3.06954600	1.47286100
C	-1.53506100	3.57280900	0.61830000
C	-1.06665600	4.26786700	-0.67315200
C	-0.00368600	3.48521100	-1.38741900
H	-0.72810700	2.43957000	2.29106300
H	-2.17344800	2.72453400	0.34983100
H	-2.15882300	4.25457700	1.20695700
H	-1.92047000	4.44550900	-1.33922400
H	-0.66563600	5.26932000	-0.43697900
H	0.16517700	3.67199400	-2.44472000
H	0.14300600	3.92498000	1.95708800
H	1.61573000	2.09260900	-1.25784700
H	1.45133500	1.78878600	1.18576300

9b

S	-1.88720500	-1.27342500	0.59479900
O	-2.01346200	-0.16977100	1.56698800
O	-1.68254000	-2.63549200	1.06653700

S	2.91498000	0.13466900	0.04919900
O	4.06466600	0.69148400	0.75568400
O	2.63767100	0.59101600	-1.33264200
C	-3.50576200	-1.29090200	-0.32952200
C	3.18680100	-1.70490800	-0.07110300
F	-3.74056500	-0.06964900	-0.86637700
F	-4.50367700	-1.57911800	0.50500000
F	-3.47671600	-2.18362400	-1.31668200
F	2.13847300	-2.27644500	-0.66782000
F	4.28480100	-1.93323900	-0.79618500
F	3.34238800	-2.22152100	1.14631900
O	-0.93306600	-0.86868900	-0.54820000
O	1.61549300	0.20034900	0.88452900
Cu	0.10821500	0.72761400	-0.21533700
C	-0.02297800	2.40747800	-1.40161200
C	0.76911000	2.85143600	-0.32787300
C	0.18236100	3.49484900	0.89203400
C	-1.22202600	2.97374700	1.25379600
C	-2.10871400	2.68621300	0.02559300
C	-1.36624500	2.09660200	-1.13967600
H	0.87006300	3.38068500	1.73580300
H	-1.12414700	2.04113300	1.82008800
H	-1.72492400	3.68621700	1.91427100
H	-2.93726400	2.02993000	0.30021200
H	-2.56068300	3.61531000	-0.36602000
H	-1.94471500	1.58360100	-1.90343000
H	0.15872300	4.57581700	0.66525600
H	0.45001300	2.10732100	-2.33360900
H	1.84260300	2.91338600	-0.48303300

10

S	-1.10251400	2.19061800	1.44666400
O	-1.39059600	2.50918600	2.83860900
O	-2.17932700	2.17912700	0.43891500
S	-3.16268100	-1.65841300	-0.39201700
O	-3.50420500	-3.07291900	-0.36393200
O	-2.14222700	-1.22113400	-1.44400100
C	0.09496300	3.50013900	0.87341900
C	-4.71910500	-0.72292400	-0.82807600
F	1.27305900	3.36177000	1.49112000
F	-0.39889500	4.71047000	1.13171700
F	0.29382500	3.39351700	-0.45413000
F	-4.44132400	0.56756600	-0.97651900
F	-5.20801100	-1.21215100	-1.96808500
F	-5.60681500	-0.88778300	0.14990600
O	-0.24615400	0.91313100	1.27594600
O	-2.64689100	-1.02461200	0.88169200
Cu	-1.03403000	-0.16675900	-0.13111300
N	1.85235200	-1.37840300	-0.30507100
C	1.64406600	-0.29928800	-1.11966300
O	0.49583600	0.13284700	-1.37900500
C	2.81986300	0.31239700	-1.78874200
C	3.86527700	-0.47657300	-2.29627700
C	2.83531100	1.70270600	-1.97877700
C	4.92319800	0.12428500	-2.97510700
H	3.83156700	-1.55611900	-2.18460900
C	3.90474000	2.29711200	-2.64488700
H	2.02090500	2.30323700	-1.59119200
C	4.94682200	1.51126700	-3.14403400
H	5.72331200	-0.48909500	-3.37892200

H	3.92201700	3.37459400	-2.77896500
H	5.77403300	1.97837600	-3.67107000
O	0.66751600	-1.98580000	0.15137700
C	0.33076800	-3.12325000	-0.68017000
H	-0.56172200	-3.54786100	-0.21837400
H	0.10210800	-2.80235000	-1.69950000
H	1.14764500	-3.84852600	-0.67155100
C	2.95760400	-1.47460500	0.69575400
C	2.51099800	-0.91882300	2.03265400
C	3.48526700	-2.90892300	0.82217200
H	3.74465000	-0.83727700	0.27919600
C	2.90444800	-1.44695300	3.19559300
H	1.85085600	-0.05543900	2.01010500
C	4.48871700	-2.99361900	1.98156900
H	2.64487600	-3.58299300	1.02531400
H	3.94686900	-3.22666300	-0.12078000
C	3.81824500	-2.63981400	3.31858100
H	2.54787000	-0.99717000	4.12072100
H	4.92464600	-3.99806400	2.02536200
H	5.31824000	-2.29801200	1.79401600
H	4.58130400	-2.44638300	4.08434400
H	3.23909500	-3.49860600	3.69239200

Cu(OTf)$_2$

S	2.42162800	0.84611900	-0.00037800
O	1.49675600	0.56785600	1.18781300
O	3.18824800	2.07439900	-0.00019900
S	-2.42183800	-0.84627300	-0.00025300
O	-1.49720600	-0.56870300	1.18813800
O	-3.18883900	-2.07431300	-0.00096700

C	3.62184300	-0.58796500	0.00040500
C	-3.62158500	0.58822400	0.00044100
F	2.92944500	-1.72983500	-0.00113800
F	4.37540000	-0.51968700	1.09129100
F	4.37854100	-0.51862000	-1.08822000
F	-4.37732400	0.51985400	-1.08891400
F	-4.37614800	0.51955200	1.09060200
F	-2.92875600	1.72985800	0.00023600
O	1.49734600	0.56749600	-1.18882800
O	-1.49724600	-0.56731600	-1.18850800
Cu	-0.00003800	-0.00015700	-0.00032100
